# Identification of Key Pathways and Establishment of a Seven-Gene Prognostic Signature in Cervical Cancer

**DOI:** 10.1155/2022/4748796

**Published:** 2022-02-04

**Authors:** Ran An, Silu Meng, Hua Qian

**Affiliations:** ^1^Department of Dermatology, Children's Hospital of Soochow University, Suzhou, Jiangsu, China; ^2^Tongji Medical College, Huazhong University of Science and Technology, Wuhan, Hubei, China

## Abstract

Cervical cancer (CC) remains high morbidity and mortality. We aimed to identify critical pathways underlying cervical carcinogenesis and establish a prognostic signature. Six datasets from the gene expression omnibus (GEO) database were used to screen the differentially expressed genes (DEGs) between CC and normal tissues. We used the unions of the DEGs to perform functional analysis. The 108 overlapped DEGs were analyzed to determine a prognostic signature by Cox regression and Lasso analysis based on The Cancer Genome Atlas (TCGA) database. Gene Set Enrichment Analysis (GSEA) and Immune Cell Abundance Identifier (ImmuCellAI) were used to determine the relationships between the signature and biological functions. The PI3K-Akt signaling pathway, the Ras signaling pathway, and the viral carcinogenesis pathway may be critical for CC development. We identified seven genes (PLOD2, DSG2, SPP1, CXCL8, MCM5, HLTF, and KLF4) to construct a risk score formula. Survival analysis showed that the high-risk group indicated a worse prognosis than the low-risk group (*p* < 0.0001). The AUC of the prognostic signature was 0.7449, 0.7641, and 0.8146 at 1, 3, and 5 years. We also identified that the signature is an independent prognostic factor. GSEA showed five pathways were relevant to the signature, such as the adherens junction pathway. The signature also affected the abundances of various types of immune cells, such as B cell, CD4+ T cell, and CD8+ T cell. Further, we found that SPP1 was co-expressed with HK3, CD163, CCL3, CLEC5A, MMP8, TREM1, OLR1, and TREM2. The results of Gene Ontology analysis showed that SPP1 and its co-expressed related proteins mainly affected metabolic process, multicellular organismal process, cell communication, cell proliferation, protein binding, and transporter activity. In conclusion, the present study explored the key pathways for CC development and the seven-gene signature can effectively make the prognosis evaluation of CC patients.

## 1. Introduction

Cervical cancer (CC) is the fourth most commonly diagnosed cancer and the fourth leading cause of cancer-associated mortality in women worldwide, with 570,000 new cases and 311,000 deaths estimated for 2018. The incidence of CC has tended to be younger in recent years [1–4]. The role of human papillomavirus (HPV) infection has been well established [5, 6]. With the application and development of high-throughput sequencing, many studies have reported many vital genes and pathways in CC carcinogenesis, such as PIK3CA, FBXW7, EP300, MAPK signaling pathway, etc. [7–9]. However, the detailed mechanism of carcinogenesis of CC is still unveiled and needs to be further studied. In clinical practice, early-stage CC patients are mainly treated with surgery, and late-stage patients are treated with chemoradiotherapy [10]. However, the rate of recurrence of CC is approximately 20%–25%, and the 5-year survival rate for late-stage CC is less than 50% [11]. The International Federation of Gynecology and Obstetrics (FIGO) stage system has been one of the CC's most important prognostic factors, while the significant differences in survival rate are reported in the same FIGO stage [12]. Therefore, it is crucial to identify sensitive and specific biomarkers that could predict the prognosis of CC patients and monitor patients' outcomes.

Based on the TCGA database and GEO database, we aim to understand the mechanism of CC carcinogenesis and predict the prognosis of CC patients more precisely. Differentially expressed genes (DEGs) were identified using six datasets in the GEO database, and these DEGs were processed to find the key pathways in CC development. Besides, we found out the genes related to the prognosis of patients from among the DEGs and established a model to predict patients' prognosis. To gain further insight into the biological pathways and immune cells changes involved in CC pathogenesis related to this prognostic model, Gene Set Enrichment Analysis (GSEA) and immune cells abundance analysis were performed (Supplementary Figure 1).

SPP1 is a secreted glycophosphoprotein of the SIBLING family [13]. Deregulation of SPP1 has been identified in various cancers [13–15]. Zhao et al. establish a five-gene prognostic model for CC, of which SPP1 is among the genes. The functions of SPP1 include bone metabolism, immune regulation, wound healing, cell survival and tumor progression. In this study, SPP1 may play an important role in the development of cervical cancer. However, its detailed function and mechanism are unclear. We thereforeexplored the function of SPP1 in this study. 

## 2. Methods

### 2.1. Quality Control and DEGs Identification

We recruited six gene expression profiles of CC (GSE6791, GSE63514, GSE7803, GSE9750, GSE39001, and GSE52903) from the gene expression omnibus (GEO) database (https://www.ncbi.nlm.nih.gov/geo) in this study. The information of datasets is shown in Table 1. We downloaded the normalized data of GSE6791, GSE63514, GSE7803, GSE39001, and GSE52903. However, the data of GSE9750 was not normalized, and we normalized its raw data through the R “affy” package with the RMA algorithm method [16]. R “limma” package was used to screen DEGs between normal and CC tissues [17]. Genes with |LogFC| >1 and adjust *p* value <0.05 were considered as DEGs in this study.

### 2.2. Functional Analysis Based on DEGs

The union of DEGs of the six datasets was processed to Kyoto Encyclopedia of Genes and Genomes (KEGG) and Gene Ontology (GO) analysis by R “clusterProfiler” package [18]. The *p* value cutoff and *q* value cutoff in this study were 0.01 and 0.05, respectively.

### 2.3. Download and Collation of CC Data in TCGA Database

The gene expression data of the 304 CC cases (Workflow Type: HTSeq – FPKM-UQ) and corresponding clinical information were downloaded from The Cancer Genome Atlas (TCGA) database (https://portal.gdc.cancer.gov/) by R “TCGAbiolinks” package [19]. The information of distant metastasis, tumor status, vital status, and overall survival (OS) was from the article of Liu et al. published in Cell in 2018 [20].

### 2.4. Conduction of the Risk Formula for Prognostic Prediction

The risk score formula was constructed using the data of CC of the TCGA database. First, we used the Venn diagram to find the overlapped DEGs of the six datasets and finally got 108 DEGs. By performing univariate Cox regression analysis with R “survival” package, the association between the expression of 108 DEGs and patient's OS was assessed. 26 DEGs with a *p* value of less than 0.05 were included in the subsequent analysis. Second, based on the above identification of prognosis‐related genes for CC, we further needed to narrow the gene range and establish a prognostic signature. Thus, we performed the Least absolute shrinkage and selection operator (Lasso) analysis by R “glmnet” package, which constructs a more refined model using a penalty function. This method can reduce the model's complexity and reduce the weight of some unimportant indicators to 0, and 13 DEGs were left in this step. Next, we used the 13 DEGs to perform a multivariate Cox regression analysis. In this step, we further optimized the model based on the Akaike's Information Criterion (AIC) value, and finally 7 genes (Procollagen-Lysine, 2-Oxoglutarate 5-Dioxygenase 2 (PLOD2), Desmoglein 2 (DSG2), Secreted Phosphoprotein 1 (SPP1), C-X-C Motif Chemokine Ligand 8 (CXCL8), Minichromosome Maintenance Complex Component 5 (MCM5), Helicase Like Transcription Factor (HLTF), Kruppel Like Factor 4 (KLF4)) were left. A risk formula (prognostic signature) was then established based on a linear combination of these DEGs' expression levels, weighted by their regression coefficients derived from the multivariate Cox regression model. The formula was as follows: risk score = [Expression level of PLOD2 *∗* (0.252751)] + [Expression level of DSG2 *∗* (0.381041)] + [Expression level of SPP1 *∗* (0.170055)] + [Expression level of CXCL8 *∗* (0.163175)] + [Expression level of MCM5 *∗* (−0.514752)] + [Expression level of HLTF *∗* (−0.245823)] + [Expression level of KLF4 *∗* (−0.184198)]. Finally, a risk score was computed for each patient with this formula and patients were classified into high-risk and low-risk group, by taking the median risk score as a cutoff point. To detect the seven genes function, COXPREdb (https://coxpresdb.jp/) was used to find the top 200 co-expressed genes of the seven genes and then these co-expressed were processed to KEGG analysis by R “clusterprofiler” package [18].

### 2.5. Assessment of the Signature's Effect

We ranked each patient's risk score and counted the number of deaths in the high-risk group and the low-risk group. Kaplan-Meier estimate based on the log-rank test was used to compare the survival difference between the high-risk group and the low-risk group by R “survminer” package. Receiver operating characteristic (ROC) curves were employed to compare the sensitivity and specificity of the survival prediction based on the risk score model by R “survivalROC” package. To test whether the prognostic signature was independent of clinical variables, we performed the univariate Cox regression analysis, and variables with *p* value <0.05 were then analyzed by multivariable Cox regression analysis. Then the independent prognostic factor was chosen to construct the nomogram by using R packages “rms” and “forestplot,” and the ROC curves of the predictive nomogram were also performed. All statistical analyses were finished with R version 3.6.3.

### 2.6. Gene Set Enrichment Analysis (GSEA)

GSEA (version 4.1.0) was used to explore the signaling pathways related to the risk score model [21]. The phenotypes were determined by the cutoff value of the risk score. The annotated gene set was selected (c2.cp.kegg.v7.1.symbols.gmt) as the reference gene set. Gene set permutations were performed 1000 times for analysis. The normalized enrichment score (NES), nominal *p* value, and false discovery rate (FDR) *q* value were used to sort the pathways enriched in each group. Pathways with NES >1, nominal *p* value <0.05 and FDR *q* value <0.25 were selected out.

### 2.7. Immune Cell Component Analysis

CXCL8 is an immune-related gene, and we therefore used the Immune Cell Abundance Identifier (ImmuCellAI), which estimates the abundance of 24 immune cells, to detect whether the prognostic signature will affect the immune microenvironment [22].

### 2.8. Linkedomics Database Analysis

We further analyzed the cancer-promoting mechanism of SPP1 by using LinkedOmics analysis software based on the TCGA database. The co-expression-related proteins of SPP1 were searched using LinkedOmics database. Cancer types to be studied were directly selected (38 cancers in total). Cervical cancer was selected as an example in this study. Analysis was carried out according to molecular typing, staging, and other data. The statistical analysis method was person correlation coefficient analysis. Then, GO analysis was performed on SPP1 and its co-expressed related proteins.

## 3. Results

### 3.1. Quality Control and DEGs Identification

**Figure 1:**
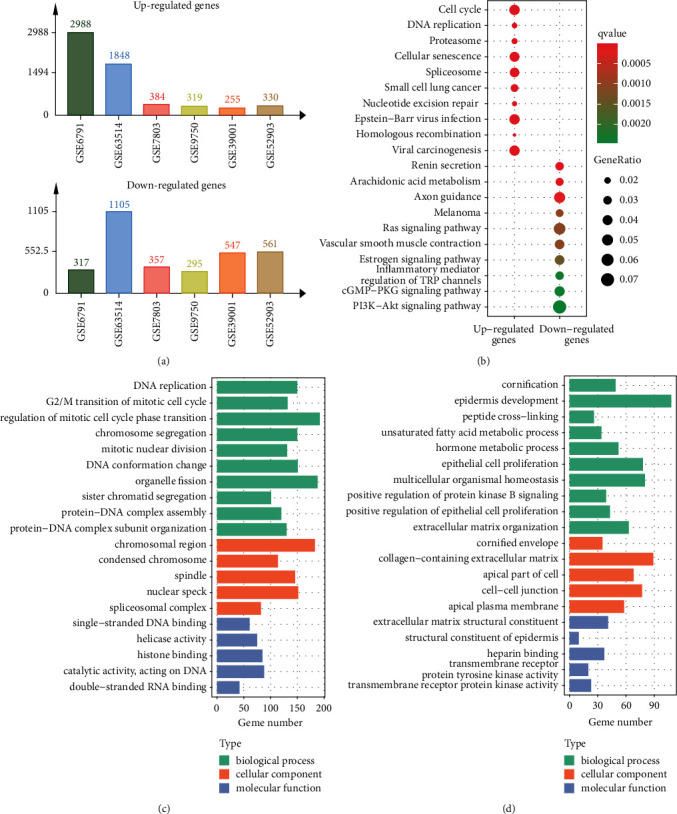
The number of DEGs in each dataset and KEGG and GO analysis of DEGs. (a) The number of DEGs in each dataset. (b) KEGG analysis. (c) GO analysis of upregulated genes. (d) GO analysis of downregulated genes. DEGs, differentially expressed genes; KEGG, Kyoto Encyclopedia of Genes and Genomes; GO, Gene Ontology.

We selected six gene expression datasets of CC (GSE6791, GSE63514, GSE7803, GSE9750, GSE39001, and GSE52903) to assess the DEGs between CC and normal tissues. The quality control results of the six datasets are shown in Supplementary Figure 2. The information of the datasets and the number of DEGs of each dataset are shown in Table 1 and Figure 1(a).

### 3.2. Functional Analysis Based on DEGs

The union of DEGs of the six datasets was processed to KEGG and GO analysis. In the KEGG analysis, the upregulated genes enriched in many well-known pathways, such as the cell cycle, the DNA replication, and the nucleotide excision repair (Figure 1(b)). And the downregulated genes are related to many cancer-related pathways, such as the Ras signaling pathway and the PI3K-Akt signaling pathway (Figure 1(b)). In the GO analysis, the upregulated genes mainly participate in the biological process (BP) related to cell cycle, such as the DNA replication, the G2/M transition of mitotic cell cycle, the regulation of mitotic cell cycle phase transition, etc. (Figure 1(c)). While the downregulated genes mainly enrich in BP of the cornification, the epidermis development, the peptide cross-linking, etc. (Figure 1(d)). The results of cellular component (CC) and molecular function (MF) are also shown in Figures 1(c) and 1(d).

### 3.3. Information of CC in the TCGA Database

We included and counted the CC's clinical information, including age, keratinization, FIGO stage, differentiation, and lymphovascular invasion. And the information of distant metastasis, tumor status, vital status, and OS information was from the article Liu et al. (Table 2) [20]. Also, the gene expression data and clinical information were matched, which were used for the subsequent survival analysis.

### 3.4. Establishment of a Seven-Genes Signature for Prognosis Prediction

**Figure 2:**
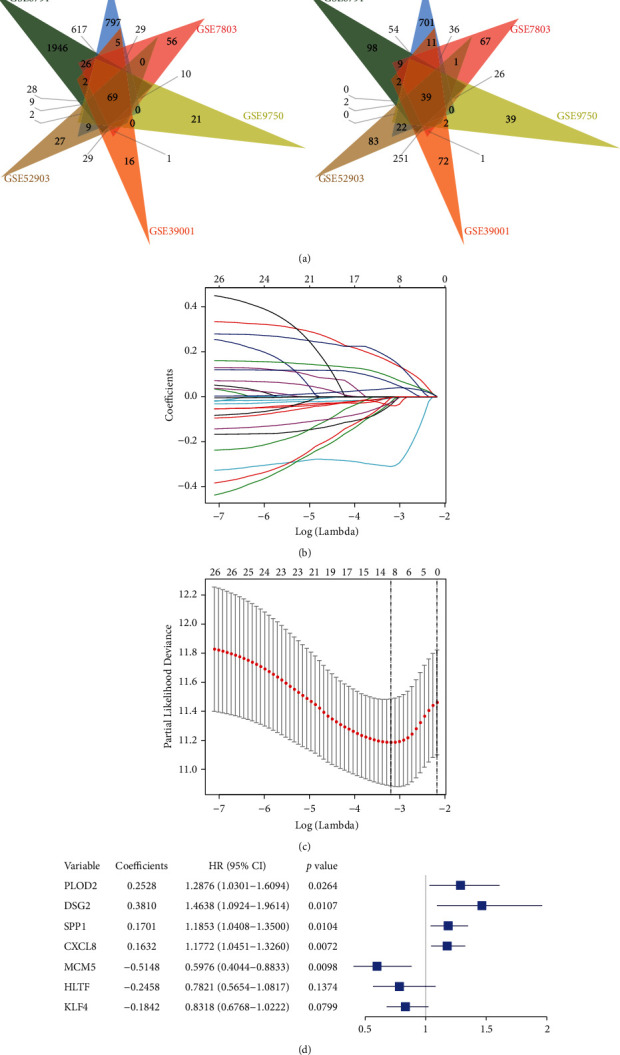
Construction of the seven-gene prognostic signature for cervical cancer. (a) The overlapped upregulated genes. (b) The overlapped downregulated genes. (c) The lasso analysis identified the most correlated genes with prognosis. (d) The distribution of each lambda and confidence interval. (e) Further narrowing of the gene range by multivariate Cox analysis and 7 genes was determined. Lasso, least absolute shrinkage and selection operator.

The Venn diagram shows the 108 overlapped DEGs of the six datasets, of which 69 were upregulated genes and 39 were downregulated genes (Figure 2(a)). The names of the 108 DEGs are shown in the Supplementary Table 1. The relationship between the 108 DEGs and the patient's OS was assessed by univariate Cox regression analysis, and 26 DEGs whose parameter *p* values were less than 0.05 were chosen for subsequent analysis (Supplementary Table 2). Considering the number of genes and collinearity, Lasso regression analysis was used to narrow the gene range, and only 13 genes were remained in this step (Figures 2(b) and 2(c)). Next, the 13 genes were processed to the multivariate Cox regression model, and we further optimized the model based on the AIC value. Finally, 7 genes (PLOD2, DSG2, SPP1, CXCL8, MCM5, HLTF, KLF4) were screened out as the predictor signature and their detailed information including coefficients, HR value, and *p* value is shown in Figure 2(d). Of these, positive coefficients for the PLOD2, DSG2, SPP1, and CXCL8 indicated that their upregulated levels of expression were associated with shorter survival. The negative coefficient of MCM5, HLTF, and KLF4 indicated that upregulated level of expression was associated with longer survival. A prognostic model based on the coefficients was established and the risk score formula was as follows: risk score = [Expression level of PLOD2 *∗* (0.252751)] + [Expression level of DSG2 *∗* (0.381041)] + [Expression level of SPP1 *∗* (0.170055)] + [Expression level of CXCL8 *∗* (0.163175)] + [Expression level of MCM5 *∗* (−0.514752)] + [Expression level of HLTF *∗* (−0.245823)] + [Expression level of KLF4 *∗* (−0.184198)]. We then calculated the seven-genes signature risk score of each patient in using the above formula. The median risk score (0.3865) was used as the cutoff point to divide the patients into two groups, the high-risk group (*N* = 152), and the low-risk group (*N* = 152) (Supplementary Table 3). In addition, to understand the function of the seven genes, the co-expressed genes were processed to KEGG analysis and they enriched in many cancer-related pathways, such as the PI3K-Akt signaling pathway, the Toll-like receptor signaling pathway, and the P53 signaling pathway. And the co-expression genes of PLOD2 and DSG2 also enriched in the Human papillomavirus infection which is inseparable with CC development (Supplementary Figure 3).

### 3.5. Assessment of the Signature's Effect

**Figure 3:**
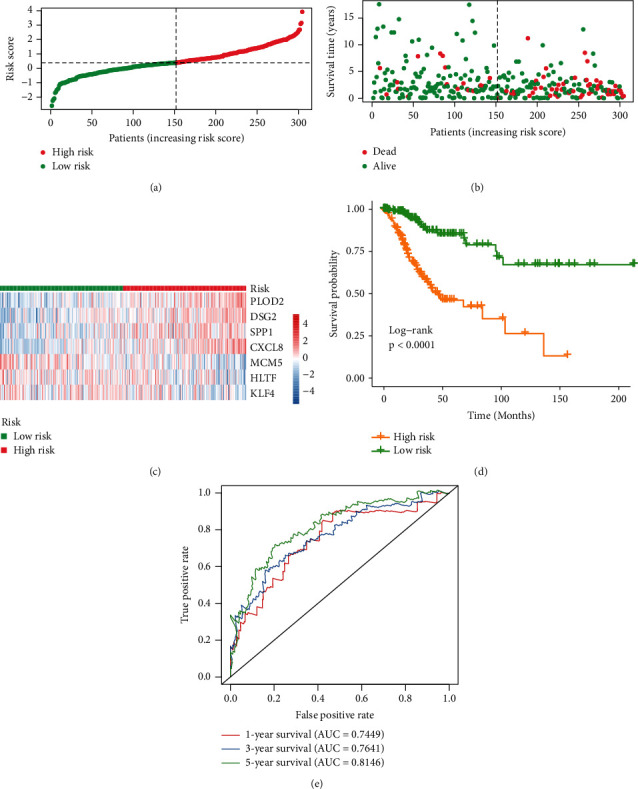
Assessment of the prognostic signature. (a) Distribution of risk score in the high-risk group and the low-risk group. (b) Survival status between the high-risk group and the low-risk group. (c) The heatmap of expression profile of 7 genes. Red parts represent upregulation, blue parts represent downregulation, and white parts represent no differential expression. (d) Survival analysis showed that the patients in the high-risk group had statistically significantly worse overall survival than those in low-risk group based on TCGA database. (e) ROC analysis was performed to calculate the AUC of 1-, 3-, and 5-year survival for this prognostic signature. TCGA, The Cancer Genome Atlas; ROC, receiver operating characteristic; AUC, area under the curve.

**Figure 4:**
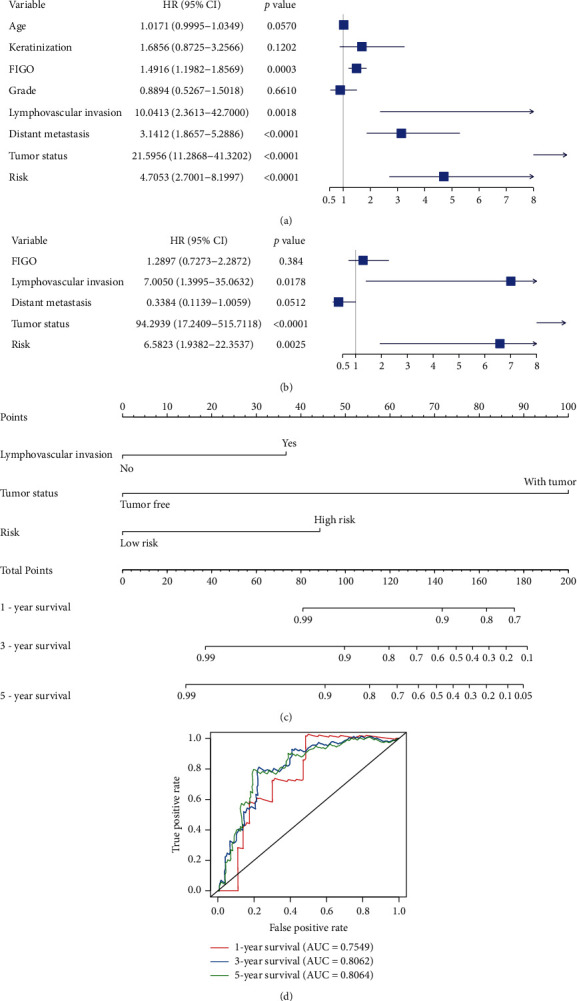
The Cox regression analysis for evaluating the independent prognostic value of the risk score. (a) The univariate Cox analysis. (b) The multivariate Cox analysis. (c) The nomogram to predict the probabilities 1‐year, 3‐year, and 5‐year OS in patients. (d) ROC curves according to the nomogram. Age, continuous variable; keratinization, yes vs. no; FIGO, continuous variable at I, II, III, and IV stages; grade, ≥G3 vs. <G3; lymphovascular invasion, yes vs. no; distant metastasis, yes vs. no; tumor status, with tumor vs. tumor free; ROC, receiver operating characteristic.

The samples were ranked according to their risk scores (Figure 3(a)) and the number of deaths increased significantly with the increase of the risk score (Figure 3(b)). A heatmap was visualized to demonstrate the expression profiles of the 7 genes (Figure 3(c)). We identified that the OS of the high-risk group is significantly shorter than the low-risk group by Kaplan-Meier method (Figure 3(d)). To further investigate the discrimination power of the signature, ROC curves were further performed. The area under the curve (AUC) of the signature was 0.7449, 0.7641, and 0.8146 at 1, 3, and 5 years (Figure 3(e)). To test whether the signature was independent of clinical variables, univariate and multivariate Cox regression analysis were performed, showing that lymphovascular invasion (HR, 7.0050; 95%CI, 1.3995–35.0632; *p*=0.0178), tumor status (HR, 94.2939; 95%CI, 17.2409–515.7118; *p* < 0.0001), and the signature (HR, 6.5823; 95%CI, 1.9382–22.3537; *p*=0.0025) are the independent predictor of poor overall survival (Figures 4(a) and 4(b)). Next, we established a nomogram combining the three independent factor (Figure 4(c)). The AUC for 1-, 3-, and 5-year survival using the predictive nomogram reached 0.7549, 0.8062, and 0.8064, respectively (Figure 4(d)).

### 3.6. Identification of the Seven-Gene Signature Altered Pathways

**Figure 5:**
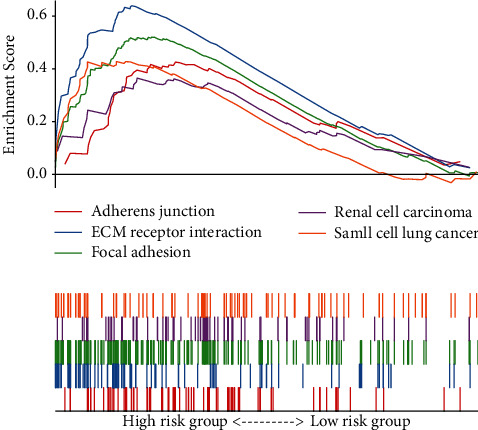
A merged enrichment plot from gene set enrichment analysis (GSEA) including enrichment score and gene sets.

To identify potentially altered signaling pathways related to the signature, we performed GSEA analysis based on the risk score classification. We selected out 5 significantly enriched signaling pathways based on the standard NES >1, nominal *p* value <0.05, and FDR *q* value <0.25, including adherent junction, ECM-receptor interaction and focal adhesion, etc. (Figure 5 and Supplementary Table 4).

### 3.7. Immune Cell Component Analysis

**Figure 6:**
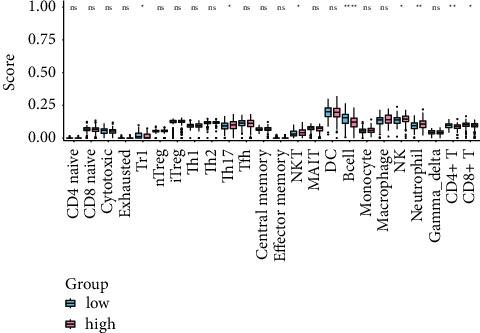
Immune cell abundance analysis between the high-risk and the low-risk group. ^*∗*^*P* < 0.05, ^*∗∗*^*P* < 0.01, ^*∗∗∗*^*P* < 0.001, and ^*∗∗∗∗*^*P* < 0.0001.

ImmuCellAI was used to analyze the difference of immune cells between the high-risk and the low-risk group (Supplementary Table 5). The abundance of Tr1 cells, B cells, CD4+ T cells, and CD8+ T cells were significantly increased in the low-risk group, while the Th17 cells, NKT cells, NK cells, and Neutrophil were significantly increased in the high-risk group (Figure 6).

### 3.8. Analysis of SPP1 Co-Expressed Related Proteins and Gene Ontology

**Figure 7:**
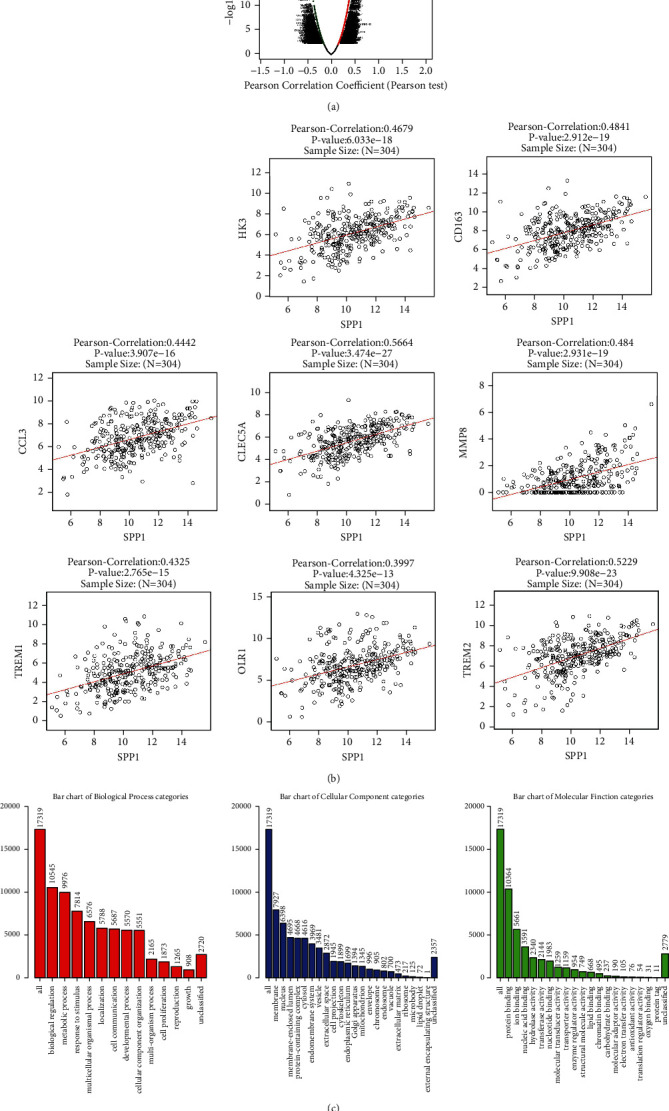
Analysis of SPP1 co-expressed related proteins and Gene Ontology. (a) SPP1 co-expressed related proteins. (b) SPP1 was positively correlated with HK3, CD163, CCL3, CLEC5A, MMP8, TREM1, OLR1, and TREM2. (c) Results of functional enrichment analysis of SPP1 and its co-expressed proteins.

We further analyzed the cancer-promoting mechanism of SPP1 by using LinkedOmics (https://www.linkedomics.org/login.php) analysis software based on the TCGA database (Figure 7(a)). Through analysis, we found that SPP1 was co-expressed with HK3, CD163, CCL3, CLEC5A, MMP8, TREM1, OLR1, and TREM2 (Figure 7(b)). The results of GO analysis showed that SPP1 and its co-expressed related proteins mainly affected metabolic process, multicellular organismal process, cell communication, cell proliferation, protein binding, and transporter activity (Figure 7(c)). These results further elucidate the carcinogenic mechanism of SPP1, which increased the understanding of SPP1.

## 4. Discussion

The morbidity and mortality of CC remain high. Although the role of HPV in CC has been well established and many researches have been done in uncovering the potential molecular mechanism of CC development, the underlying mechanism remains unclear. The surgical and chemoradiotherapy of CC are very mature, while many patients still suffered from recurrence and metastasis. The present research performed an integrated bioinformatical analysis based upon six mRNA expression profile datasets of the GEO database and 304 CC patients of the TCGA database to further uncover the mechanism of CC and discover more promising and valuable prognosis-related biomarkers in CC.

We used the union of the DEGs of the six datasets to perform KEGG and GO analysis. In the KEGG analysis, the upregulated genes enriched in the cell cycle, the DNA replication, the nucleotide excision repair, etc., and the DEGs may promote cell proliferation by being involved in these pathways. These pathways are common in various kinds of cancer, such as colorectal cancer and gastric cancer [23–25]. In addition, the upregulated genes also enriched in the viral carcinogenesis, which may also prove the role of HPV in the development of CC. The downregulated genes enriched in the PI3K-Akt signaling pathway and the Ras signaling pathway, which have been reported to play critical roles in CC [9]. In the GO analysis, the upregulated genes were mainly related to cell cycle related terms, such as the DNA replication, the G2/M transition of mitotic cell cycle, and the regulation of mitotic cell cycle phase transition. This result is similar to that of KEGG to a large extent. In contrast, the downregulated genes enriched in many epidermis-related pathways, such as epidermis development, epithelial cell proliferation, positive regulation of epithelial cell proliferation, and structural constituent of epidermis. In summary, the enriched GO terms and KEGG pathways explained the specific molecular mechanisms of CC to some extent.

We identified 108 common DEGs between the CC tissues and normal tissues of the six datasets, including 69 upregulated genes and 39 downregulated genes. We further analyzed the relationship between the prognosis of CC patients and the expression levels of the 108 DEGs by univariate Cox analysis, of which 26 DEGs indicated the significant correlation with OS (*p* < 0.05). Next, we performed both lasso and multivariate Cox analysis to narrow the gene range, and finally we got 7 genes (PLOD2, DSG2, SPP1, CXCL8, MCM5, HLTF, KLF4) to establish a prognostic model which was able to distinguish CC patients into the high-risk group and low-risk group. PLOD2 encodes the key enzyme mediating the formation of the stabilized collagen cross-link, which sometimes can be considered as the “highway” for cancer cell migration and invasion [26]. PLOD2 overexpressed in many cancers, including hepatocellular carcinoma, breast cancer, and sarcoma [26]. Overexpression of PLOD2 can also induce cell migration and invasion in CC and PLOD2 is correlated with the prognosis of CC patients [27, 28]. DSG2 is a cell adhesion protein of the cadherin superfamily, which can regulate cell-cell contact. And DSG2 has been reported to play key roles in tumorigenesis [29, 30]. Qin et al. have reported that DSG2 can promote tumor proliferation and metastasis and is correlated with poor prognosis in early-stage CC [31]. CXCL8 is an important cytokine that can modulate proliferation, invasion, and migration of tumor cells and can induce tumor immunosuppression. It has been reported that the CXCL8-CXCR1/2 axis has the potential to be applied as a cancer therapeutic target [32]. Yan et al. found that CXCL8 high expression was a poor independent prognostic parameter for CC patients [33]. MCM5 is a member of the MCM family of chromatin-binding proteins and participates in cell cycle regulation [34]. MCM5 has been reported as a predictive biomarker for both cervical preinvasive neoplasia and CC [35]. HLTF belongs to the SWI/SNF family of proteins involved in chromatin remodeling and DNA repair, suggesting that it acts as a tumor suppressor gene [36]. HLTF expression is altered in cancer through two mechanisms: gene silencing by promoter hypermethylation or expression of truncated proteins that lack functional domains [36]. These mechanisms have been widely proved in digestive tract cancers [37, 38]. In CC, Cho et al. showed overexpression of HLTF might confer patients with resistance to radiation [39]. KLF4 functions both as a tumor suppressor and an oncogene, which is involved in cell differentiation and cell-cycle arrest [40]. It has shown that KLF4 can regulate cell proliferation, migration, and invasion in multiple cancers, including breast cancer, gastrointestinal cancer, and esophageal cancer [41–43]. Yang and Zheng identified the tumor suppressor role of KLF4 in CC and found that promoter hypermethylation of KLF4 can inactive its tumor suppressor function in CC [44, 45]. To further understand the function of the 7 genes we selected, the top 200 co-expressed genes of the 7 genes were processed to KEGG analysis, which shows many critical pathways for CC development, including the PI3K-Akt signaling pathway, the Huam papillomavirus infection, and the p53 signaling pathway.

Based on the prognostic signature, the number of deaths increased significantly with risk score increase. Kaplan–Meier survival analysis showed that the high-risk group indicated a worse prognosis than the low-risk group (*p* < 0.0001). The AUC of the risk model was 0.7449, 0.7641, and 0.8146 at 1, 3, and 5 years. Next, the univariate and multivariate Cox regression analyses were performed, identifying that lymphovascular invasion, the tumor status, and the signature are independent prognostic factors in CC. The AUC of the predictive nomogram combining the three independent factors for 1, 3, and 5-year survival reached 0.7549, 0.8062, and 0.8064.

To further investigate the model's underlying mechanism, we performed the GSEA to explore the signaling pathways related to the risk score model, and 5 pathways were selected out. The adherens junction pathway is an element of cell-cell junction, essential for maintaining tissue architecture and cell polarity and can regulate cell movement and proliferation. E-cadherin, and *α*- and *β*-catenin are the main components of AJ [46]. Loss or downregulation of E-cadherin expression is frequently observed in cancers and correlates with the malignancy of the tumors [47]. Fujimoto et al. have reported that decreased expression of main adhesion molecules may result in invasiveness and metastasis of CC [48]. Li et al. identified that the genes at breakpoints of HPV integration in CC also enrich in the adherens junction pathway [49]. The ECM-receptor interaction pathway controls many cellular activities such as adhesion, migration, differentiation, proliferation, and apoptosis [50]. This pathway participates in the progression of various cancers such as breast cancer, prostate cancer, and gastric cancer [51–53]. Using two datasets of the GEO database, Wu et al. found that the ECM-receptor interaction pathway is the key pathway during CC development [54]. Focal adhesion refers to the specialized structures at cell-extracellular matrix contact points, which play essential roles in important biological processes, including cell motility, cell proliferation, and cell differentiation [55]. Increased expression and amplification of the focal adhesion kinase gene in human cancer cells are common [56]. Xu et al. showed that PLOD2 could improve the migration and invasion of CC cells by focal adhesion formation [27]. We also found the other two pathways in this study, the renal cell carcinoma and small cell lung cancer pathways, which may indicate that the underlying molecular mechanism of CC development may coincide with the two kinds of cancer. In addition, we analyzed whether the risk model affects the immune microenvironment. The abundance of Tr1 cells, B cells, CD4+ T cells, and CD8+ T cells are significantly increased in the low-risk group, while the Th17 cells, NKT cells, NK cells, and Neutrophil are significantly increased in the high-risk group. Wang et al. found that a higher level of activated memory CD4+ T cells was independently associated with favorable OS in CC [57]. Although we don't have the result of activated memory CD4+ T cells, the abundance of CD4+ T cells is higher in the low-risk group. It has shown that both the CC patients and cervical precancerous lesion patients have a higher proportion of Th17 cells. Increased Th17 cells were associated with clinical stage, lymph node metastasis, and invasion, and therefore accelerate the disease's progression [58]. And this may partially explain the higher level of Th17 cells in the high-risk group.

It has been shown [59] that SPP1 can bind to CD44v6 and promote tumor cell proliferation and survival through JNK pathway. SPP1 also promotes the expression of cancer stem cell markers such as OCT4 and SOX2. This can not only improve the survival rate of cancer cells, but also enhance the resistance to oxaliplatin and other chemotherapy drugs [60]. In colorectal cancer patients, SPP1 induces high expression of CD44v6 through the Wnt-*β*-catenin pathway to stimulate cancer progression [61]. This study found that SPP1 is an abnormally expressed gene in the development of cervical cancer. Studies have reported that the expression level of SPP1 is closely related to the occurrence, development, invasion, and metastasis of malignant tumors [62]. In order to further understand the exact mechanism of SPP1 promoting tumor development, this study conducted in-depth bioinformatics analysis on the cancer-promoting mechanism of SPP1. Through the analysis, we found that SPP1 was co-expressed with HK3, CD163, CCL3, CLEC5A, MMP8, TREM1, OLR1, and TREM2.

In colorectal cancer, HK3 overexpression was associated with epithelial-mesenchymal transition [63]. It has been reported that OLR1 promotes pancreatic cancer metastasis via increased c-Myc expression and transcription of HMGA2 [64]. In human osteosarcoma cells, CCL3 promotes angiogenesis by dysregulation of VEGFa [65]. These results further elucidate the carcinogenic mechanism of SPP1 and increased the understanding of SPP1, that is, SPP1 may be a potential key target for the treatment of cervical cancer. However, the molecular mechanism of how it regulates cells in cervical cancer needs to be further studied.

In conclusion, we utilized the public online database to find the related pathways underlying CC development and establish a CC's prognostic model. First, the PI3K-Akt signaling pathway, the Ras signaling pathway, and the viral carcinogenesis pathway may be the critical pathways for CC development. Second, we established a seven-gene prognostic signature for CC and validated the effects of the signature. Finally, we explored the possible mechanism underlying the prognostic signature p53 signaling pathway and further elucidate the carcinogenic mechanism of SPP1.

## Figures and Tables

**Table 1:** Information of the datasets used in this study.

Datasets	Year	Platform	Participants	
			Normal	Cervical cancer
GSE6791	2007	GPL570	8	20
GSE63514	2015	GPL570	24	28
GSE7803	2007	GPL96	10	21
GSE9750	2008	GPL96	24	33
GSE39001	2013	GPL6244	5	19
GSE52903	2015	GPL6244	17	55

**Table 2:** Characteristics of the cervical cancer patients in the TCGA database.

Clinical characteristics subgroup		Frequency	Percentage
Total		304	

Age	Range: 20–88 (average: 48.2, median: 46)		

Keratinization	No	119	68.4
	Yes	55	31.6
	N1	60	31.1

FIGO	I	162	54.5
	II	69	23.2
	III	45	15.2
	IV	21	7.1

Differentiation grade	≤G2	153	56.3
	≥G3	119	43.7

Lymphovascular invasion	Absent	71	47.3
	Present	79	52.7

Distant metastasis	No	273	89.8
	Yes	31	10.2

Tumor status	Tumor free	197	71.1
	With tumor	80	28.9

Vital status	Alive	233	76.6
	Dead	71	23.4

## Data Availability

Publicly available datasets were analyzed in this study. These can be found in The Cancer Genome Atlas (https://portal.gdc.cancer.gov/) and the NCBI Gene Expression Omnibus (GSE6791, GSE63514, GSE7803, GSE9750, GSE39001, and GSE52903).

## Supplementary Materials

Supplementary Materials Supplementary Figure 1: workflow chart of this study. Supplementary Figure 2: quality control of the six datasets. Supplementary Figure 3: KEGG analysis of the top 200 coexpressed genes of the 7 genes of the prognostic signature. KEGG, Kyoto Encyclopedia of Genes and Genomes. Supplementary Table 1: 108 Common DEGs of the six datasets. Supplementary Table 2: the associations between overall survival and 108 common DEGs using univariate cox analysis. Supplementary Table 3: the risk score and risk group of each patient of the TCGA database. Supplementary Table 4: gene sets enriched in the high-risk group. Supplementary Table 5: immune cells abundance analysis of the high-risk group and the low-risk group.
