# Supplementary material for: Identification of Key Pathways and Establishment of a Seven-Gene Prognostic Signature in Cervical Cancer
Source: J Oncol. 2022 Feb 4;2022:4748796. doi: 10.1155/2022/4748796 (PMC8837458; doi:10.1155/2022/4748796)
Supplement: Supplementary Materials — Supplementary Figure 1: workflow chart of this study. Supplementary Figure 2: quality control of the six datasets. Supplementary Figure 3: KEGG analysis of the top 200 coexpressed genes of the 7 genes of the prognostic signature. KEGG, Kyoto Encyclopedia of Genes and Genomes. Supplementary Table 1: 108 Common DEGs of the six datasets. Supplementary Table 2: the associations between overall survival and 108 common DEGs using univariate cox analysis. Supplementary Table 3: the risk score and risk group of each patient of the TCGA database. Supplementary Table 4: gene sets enriched in the high-risk group. Supplementary Table 5: immune cells abundance analysis of the high-risk group and the low-risk group. [file 4748796.f1.zip › 4748796.f1/Supplementary Table 1.docx]

Supplementary Table 1. 108 Common DEGs of the six datasets.

| **Gene** | **Gene names** |
| --- | --- |
| Up-regulated genes | TPX2, CCNB1, GINS1, KNTC1, FOXM1, CDK1, MLF1, KIF14, KIF4A, SPP1, MCM10, TYMS, MCM5, MELK, ZWINT, NDC80, OIP5, PLOD2, STAT1, PTTG1, MMP12, CKS2, ECT2, LMNB1, PCLAF, TACC3, MCM7, PRC1, CEP55, MOCOS, MCM2, MCM4, TIMELESS, DLGAP5, RAD51AP1, NEK2, GINS2, RPL39L, E2F8, PLAU, HLTF, TOPBP1, EZH2, KIF11, DSG2, SMC4, CXCL8, PCNA, KIF2C, CDC20, RFC4, CDC7, ASPM, DNMT1, RRM2, TOP2A, HELLS, FANCI, SYCP2, DTL, MCM6, GMNN, KIF20A, CDKN2A, TTK, CDKN3, NCAPG, CENPF, NUSAP1 |
| Down-regulated genes | BBOX1, UPK1A, ENDOU, KRT1, CRYAB, MAL, SPINK5, KLF4, EMP1, PPL, PDGFD, ESR1, EREG, KLK12, DSG1, HPGD, CWH43, SCEL, CRISP3, IGFBP5, ALOX12, KRT4, GYS2, KLK13, APOD, CRNN, TGFBR3, SPRR3, SOSTDC1, GPX3, PPP1R3C, THSD4, ZNF91, KLK11, AR, IVL, CFD, HOPX, CRCT1 |
