# Supplementary material for: Identification of Key Pathways and Establishment of a Seven-Gene Prognostic Signature in Cervical Cancer
Source: J Oncol. 2022 Feb 4;2022:4748796. doi: 10.1155/2022/4748796 (PMC8837458; doi:10.1155/2022/4748796)
Supplement: Supplementary Materials — Supplementary Figure 1: workflow chart of this study. Supplementary Figure 2: quality control of the six datasets. Supplementary Figure 3: KEGG analysis of the top 200 coexpressed genes of the 7 genes of the prognostic signature. KEGG, Kyoto Encyclopedia of Genes and Genomes. Supplementary Table 1: 108 Common DEGs of the six datasets. Supplementary Table 2: the associations between overall survival and 108 common DEGs using univariate cox analysis. Supplementary Table 3: the risk score and risk group of each patient of the TCGA database. Supplementary Table 4: gene sets enriched in the high-risk group. Supplementary Table 5: immune cells abundance analysis of the high-risk group and the low-risk group. [file 4748796.f1.zip › 4748796.f1/Supplementary Table 2.docx]

Supplementary Table 2. The associations between overall survival and 108 common DEGs using univariate cox analysis.

|  | **beta** | **HR (95% CI for HR)** | **wald.test** | p-value |
| --- | --- | --- | --- | --- |
| EREG | 0.16 | 1.17239 (1.08776-1.26361) | 17 | 3.18E-05 |
| CXCL8 | 0.24 | 1.27262 (1.13474-1.42724) | 17 | 3.78E-05 |
| PLOD2 | 0.39 | 1.47143 (1.20345-1.79907) | 14 | 0.00016623 |
| MCM5 | -0.66 | 0.517876 (0.367367-0.730047) | 14 | 0.000172684 |
| DSG2 | 0.5 | 1.64351 (1.24488-2.16979) | 12 | 0.000455922 |
| SPP1 | 0.18 | 1.19624 (1.06157-1.34801) | 8.6 | 0.00327767 |
| PCNA | -0.57 | 0.568143 (0.382464-0.843967) | 7.8 | 0.00510798 |
| HELLS | -0.54 | 0.585137 (0.395509-0.865683) | 7.2 | 0.0073241 |
| SYCP2 | -0.12 | 0.883454 (0.804117-0.970618) | 6.7 | 0.00984708 |
| MLF1 | -0.17 | 0.847849 (0.745656-0.964047) | 6.3 | 0.0117787 |
| PCLAF | -0.32 | 0.723483 (0.556252-0.940989) | 5.8 | 0.0158003 |
| GINS2 | -0.42 | 0.658477 (0.463425-0.935624) | 5.4 | 0.0197415 |
| HLTF | -0.36 | 0.698553 (0.514175-0.949047) | 5.3 | 0.021765 |
| MCM2 | -0.35 | 0.701779 (0.518017-0.950728) | 5.2 | 0.0222456 |
| EZH2 | -0.41 | 0.661205 (0.462025-0.946253) | 5.1 | 0.0236948 |
| RFC4 | -0.39 | 0.674412 (0.47803-0.951471) | 5 | 0.0248801 |
| TYMS | -0.4 | 0.669993 (0.470975-0.95311) | 5 | 0.0259461 |
| PLAU | 0.19 | 1.20878 (1.02262-1.42882) | 4.9 | 0.0262739 |
| APOD | -0.13 | 0.880249 (0.784746-0.987374) | 4.7 | 0.0294943 |
| GMNN | -0.26 | 0.773439 (0.612538-0.976606) | 4.7 | 0.0308591 |
| ZWINT | -0.27 | 0.762498 (0.591515-0.982905) | 4.4 | 0.0363434 |
| KRT4 | -0.056 | 0.94545 (0.896352-0.997238) | 4.2 | 0.0392439 |
| SPRR3 | -0.046 | 0.955076 (0.913914-0.998092) | 4.2 | 0.0408629 |
| PTTG1 | -0.32 | 0.727148 (0.534847-0.988591) | 4.1 | 0.0420337 |
| KIF14 | 0.34 | 1.39872 (1.00828-1.94035) | 4 | 0.0444996 |
| KLF4 | -0.19 | 0.827063 (0.684298-0.999615) | 3.9 | 0.0495358 |
| LMNB1 | -0.31 | 0.735309 (0.516153-1.04752) | 2.9 | 0.0885971 |
| MAL | -0.063 | 0.939072 (0.872626-1.01058) | 2.8 | 0.0931605 |
| CRCT1 | 0.035 | 1.03554 (0.993645-1.0792) | 2.8 | 0.0974361 |
| MCM6 | -0.4 | 0.668165 (0.41105-1.08611) | 2.6 | 0.103795 |
| KLK11 | -0.048 | 0.953581 (0.899055-1.01141) | 2.5 | 0.113612 |
| FANCI | -0.34 | 0.71266 (0.464969-1.0923) | 2.4 | 0.120003 |
| GYS2 | 0.056 | 1.05783 (0.985126-1.13589) | 2.4 | 0.121752 |
| CRNN | -0.026 | 0.974449 (0.942291-1.0077) | 2.3 | 0.130612 |
| BBOX1 | -0.051 | 0.950695 (0.889735-1.01583) | 2.2 | 0.134815 |
| ASPM | 0.26 | 1.30242 (0.914732-1.85443) | 2.2 | 0.142755 |
| THSD4 | 0.1 | 1.10819 (0.963876-1.27412) | 2.1 | 0.148988 |
| MCM7 | -0.3 | 0.743956 (0.497106-1.11338) | 2.1 | 0.15048 |
| KNTC1 | -0.29 | 0.744591 (0.492319-1.12613) | 2 | 0.162355 |
| CENPF | 0.28 | 1.31713 (0.890548-1.94804) | 1.9 | 0.167756 |
| NUSAP1 | -0.24 | 0.785247 (0.553842-1.11334) | 1.8 | 0.174709 |
| KRT1 | -0.032 | 0.968434 (0.924024-1.01498) | 1.8 | 0.180513 |
| CWH43 | 0.025 | 1.02518 (0.987021-1.06481) | 1.6 | 0.198833 |
| DTL | -0.27 | 0.762675 (0.501392-1.16011) | 1.6 | 0.205525 |
| CFD | -0.099 | 0.905499 (0.773205-1.06043) | 1.5 | 0.218002 |
| EMP1 | 0.097 | 1.10199 (0.942221-1.28885) | 1.5 | 0.224272 |
| AR | -0.071 | 0.931446 (0.830105-1.04516) | 1.5 | 0.226892 |
| RPL39L | -0.094 | 0.910438 (0.778648-1.06453) | 1.4 | 0.239556 |
| ECT2 | 0.19 | 1.21055 (0.877068-1.67083) | 1.4 | 0.245173 |
| HOPX | -0.089 | 0.914876 (0.784089-1.06748) | 1.3 | 0.258338 |
| MMP12 | 0.054 | 1.05544 (0.957353-1.16357) | 1.2 | 0.278281 |
| TACC3 | -0.21 | 0.807858 (0.546445-1.19433) | 1.1 | 0.284761 |
| PPP1R3C | 0.05 | 1.05111 (0.9579-1.15338) | 1.1 | 0.292756 |
| DNMT1 | -0.2 | 0.818958 (0.562444-1.19246) | 1.1 | 0.297504 |
| CCNB1 | -0.2 | 0.818675 (0.558676-1.19967) | 1 | 0.3048 |
| TTK | 0.2 | 1.21998 (0.817678-1.82021) | 0.95 | 0.330069 |
| FOXM1 | 0.17 | 1.18873 (0.837563-1.68713) | 0.94 | 0.333174 |
| CRISP3 | 0.02 | 1.02007 (0.979251-1.0626) | 0.91 | 0.340198 |
| KIF4A | -0.2 | 0.820182 (0.544782-1.2348) | 0.9 | 0.342313 |
| KIF11 | -0.18 | 0.834955 (0.573893-1.21477) | 0.89 | 0.345721 |
| NDC80 | -0.2 | 0.817057 (0.53437-1.24929) | 0.87 | 0.351022 |
| NCAPG | 0.19 | 1.21266 (0.797788-1.84327) | 0.81 | 0.366781 |
| KIF20A | 0.14 | 1.14492 (0.848936-1.54409) | 0.79 | 0.375189 |
| RAD51AP1 | -0.16 | 0.854423 (0.601873-1.21294) | 0.77 | 0.378822 |
| DSG1 | -0.018 | 0.98226 (0.943222-1.02291) | 0.75 | 0.387012 |
| RRM2 | 0.15 | 1.15629 (0.813188-1.64415) | 0.65 | 0.418775 |
| ENDOU | 0.032 | 1.03301 (0.954744-1.11768) | 0.65 | 0.419178 |
| MOCOS | 0.075 | 1.07825 (0.893555-1.30111) | 0.62 | 0.431928 |
| TIMELESS | -0.17 | 0.845605 (0.555582-1.28702) | 0.61 | 0.433899 |
| DLGAP5 | 0.15 | 1.16332 (0.795956-1.70024) | 0.61 | 0.434616 |
| SMC4 | 0.096 | 1.10088 (0.863722-1.40315) | 0.6 | 0.4375 |
| KLK12 | -0.016 | 0.984358 (0.945406-1.02491) | 0.59 | 0.444077 |
| GPX3 | 0.046 | 1.04737 (0.925055-1.18587) | 0.53 | 0.465087 |
| PRC1 | -0.12 | 0.8841 (0.634587-1.23172) | 0.53 | 0.466547 |
| ALOX12 | -0.05 | 0.951542 (0.830076-1.09078) | 0.51 | 0.475929 |
| SPINK5 | -0.028 | 0.972271 (0.89798-1.05271) | 0.48 | 0.488074 |
| ZNF91 | 0.043 | 1.04358 (0.922149-1.181) | 0.46 | 0.499145 |
| CDC7 | -0.12 | 0.889298 (0.617864-1.27997) | 0.4 | 0.52775 |
| CEP55 | -0.11 | 0.89682 (0.636165-1.26427) | 0.39 | 0.534236 |
| STAT1 | -0.066 | 0.936345 (0.759622-1.15418) | 0.38 | 0.537689 |
| TOPBP1 | -0.11 | 0.892862 (0.619663-1.28651) | 0.37 | 0.543128 |
| PDGFD | 0.052 | 1.05317 (0.890323-1.2458) | 0.37 | 0.545532 |
| SCEL | 0.026 | 1.02648 (0.940922-1.11982) | 0.35 | 0.556148 |
| KIF2C | -0.12 | 0.883478 (0.579782-1.34626) | 0.33 | 0.5643 |
| MCM4 | -0.14 | 0.873436 (0.54769-1.39292) | 0.32 | 0.569854 |
| UPK1A | -0.019 | 0.980811 (0.916224-1.04995) | 0.31 | 0.577202 |
| MELK | 0.1 | 1.10657 (0.756711-1.61818) | 0.27 | 0.601494 |
| CDK1 | -0.11 | 0.895451 (0.575365-1.3936) | 0.24 | 0.624618 |
| CDKN2A | -0.037 | 0.963503 (0.827954-1.12124) | 0.23 | 0.630788 |
| PPL | -0.038 | 0.962576 (0.791565-1.17053) | 0.15 | 0.70232 |
| OIP5 | -0.062 | 0.940184 (0.652885-1.35391) | 0.11 | 0.740269 |
| CKS2 | -0.06 | 0.941619 (0.655269-1.3531) | 0.11 | 0.745032 |
| IVL | 0.0061 | 1.00616 (0.96379-1.05038) | 0.08 | 0.779789 |
| SOSTDC1 | 0.0059 | 1.00589 (0.965065-1.04844) | 0.08 | 0.781244 |
| TPX2 | 0.054 | 1.05572 (0.694444-1.60495) | 0.06 | 0.799711 |
| KLK13 | -0.0065 | 0.99356 (0.944828-1.0448) | 0.06 | 0.801192 |
| E2F8 | 0.029 | 1.02902 (0.81203-1.304) | 0.06 | 0.81283 |
| HPGD | -0.011 | 0.989481 (0.902633-1.08469) | 0.05 | 0.821504 |
| CDKN3 | 0.034 | 1.03508 (0.733528-1.4606) | 0.04 | 0.844431 |
| ESR1 | -0.011 | 0.988855 (0.88312-1.10725) | 0.04 | 0.845986 |
| MCM10 | 0.035 | 1.0356 (0.726143-1.47695) | 0.04 | 0.846836 |
| GINS1 | -0.03 | 0.970731 (0.663463-1.4203) | 0.02 | 0.87841 |
| NEK2 | 0.025 | 1.02491 (0.691536-1.51899) | 0.02 | 0.902454 |
| TOP2A | 0.022 | 1.0222 (0.70242-1.48756) | 0.01 | 0.908686 |
| CRYAB | -0.0068 | 0.993218 (0.881733-1.1188) | 0.01 | 0.910806 |
| IGFBP5 | 0.0063 | 1.00633 (0.894495-1.13215) | 0.01 | 0.916357 |
| CDC20 | 0.011 | 1.01066 (0.716989-1.42461) | 0 | 0.95173 |
| TGFBR3 | -0.0042 | 0.995788 (0.843794-1.17516) | 0 | 0.960165 |
