# Supplementary material for: Identification of Key Pathways and Establishment of a Seven-Gene Prognostic Signature in Cervical Cancer
Source: J Oncol. 2022 Feb 4;2022:4748796. doi: 10.1155/2022/4748796 (PMC8837458; doi:10.1155/2022/4748796)
Supplement: Supplementary Materials — Supplementary Figure 1: workflow chart of this study. Supplementary Figure 2: quality control of the six datasets. Supplementary Figure 3: KEGG analysis of the top 200 coexpressed genes of the 7 genes of the prognostic signature. KEGG, Kyoto Encyclopedia of Genes and Genomes. Supplementary Table 1: 108 Common DEGs of the six datasets. Supplementary Table 2: the associations between overall survival and 108 common DEGs using univariate cox analysis. Supplementary Table 3: the risk score and risk group of each patient of the TCGA database. Supplementary Table 4: gene sets enriched in the high-risk group. Supplementary Table 5: immune cells abundance analysis of the high-risk group and the low-risk group. [file 4748796.f1.zip › 4748796.f1/Supplementary Table 3.docx]

Supplementary Table 3. The risk score and risk group of each patient of the TCGA database.

| **submitter_id** | **OS status*** | **OS (days)** | **OS (months)** | **OS (years)** | **PLOD2** | **MCM5** | **DSG2** | **SPP1** | **CXCL8** | **HLTF** | **KLF4** | **risk score** | **risk_group** |
| --- | --- | --- | --- | --- | --- | --- | --- | --- | --- | --- | --- | --- | --- |
| TCGA-VS-A94Y | 1 | 144 | 4.8000 | 0.3945 | 20.6198 | 18.0047 | 21.0915 | 20.0880 | 24.3494 | 18.1509 | 16.2453 | 3.9155 | high |
| TCGA-VS-A9V4 | 1 | 132 | 4.4000 | 0.3616 | 19.2321 | 17.5269 | 20.7801 | 16.6036 | 21.2634 | 14.8584 | 17.6509 | 3.1464 | high |
| TCGA-EK-A2RJ | 0 | 53 | 1.7667 | 0.1452 | 19.1558 | 17.5894 | 20.3500 | 20.3880 | 21.5773 | 16.6344 | 18.2821 | 3.0730 | high |
| TCGA-C5-A0TN | 1 | 348 | 11.6000 | 0.9534 | 20.9414 | 19.8713 | 21.2498 | 19.7120 | 20.4625 | 17.0089 | 16.2663 | 2.6749 | high |
| TCGA-VS-A94X | 1 | 506 | 16.8667 | 1.3863 | 19.7040 | 18.1597 | 20.9838 | 20.7557 | 19.7391 | 17.9757 | 17.9393 | 2.6555 | high |
| TCGA-EK-A2H1 | 0 | 799 | 26.6333 | 2.1890 | 21.7284 | 18.9270 | 19.3492 | 20.9271 | 21.4959 | 17.0248 | 18.8747 | 2.5266 | high |
| TCGA-C5-A1BF | 1 | 570 | 19.0000 | 1.5616 | 20.3243 | 17.8851 | 20.1327 | 19.1477 | 19.9474 | 17.5482 | 18.1650 | 2.4533 | high |
| TCGA-ZJ-AAXN | 0 | 0 | 0.0000 | 0.0000 | 19.6003 | 18.8993 | 20.2192 | 20.5792 | 22.5186 | 17.4041 | 18.5436 | 2.4099 | high |
| TCGA-EK-A2R8 | 0 | 44 | 1.4667 | 0.1205 | 21.0137 | 19.3572 | 20.5828 | 18.2278 | 22.8488 | 17.4982 | 18.1997 | 2.3642 | high |
| TCGA-Q1-A73O | 0 | 428 | 14.2667 | 1.1726 | 20.3714 | 19.0962 | 19.8753 | 20.8382 | 21.9865 | 18.4942 | 17.2519 | 2.2996 | high |
| TCGA-C5-A8YR | 1 | 837 | 27.9000 | 2.2932 | 20.3659 | 19.1331 | 19.4557 | 22.9114 | 19.1995 | 18.0754 | 16.3716 | 2.2822 | high |
| TCGA-VS-AA62 | 1 | 469 | 15.6333 | 1.2849 | 20.2133 | 19.2787 | 20.5353 | 20.2403 | 20.8143 | 17.5311 | 17.9698 | 2.2288 | high |
| TCGA-C5-A7X3 | 1 | 284 | 9.4667 | 0.7781 | 18.9457 | 18.8219 | 19.8613 | 20.5035 | 18.6424 | 16.1739 | 16.3430 | 2.2104 | high |
| TCGA-C5-A7CL | 1 | 471 | 15.7000 | 1.2904 | 20.1596 | 19.0238 | 20.4920 | 21.3058 | 21.0917 | 18.7555 | 18.3635 | 2.1829 | high |
| TCGA-FU-A57G | 0 | 1078 | 35.9333 | 2.9534 | 18.0412 | 17.3907 | 18.9327 | 20.3674 | 21.6586 | 17.1753 | 18.6448 | 2.1634 | high |
| TCGA-EX-A3L1 | 0 | 463 | 15.4333 | 1.2685 | 19.9804 | 19.5220 | 20.7858 | 18.4257 | 23.3984 | 17.1517 | 18.9790 | 2.1606 | high |
| TCGA-Q1-A6DT | 1 | 275 | 9.1667 | 0.7534 | 20.4793 | 18.6877 | 20.0072 | 19.4785 | 22.2230 | 18.4632 | 18.7935 | 2.1184 | high |
| TCGA-FU-A3HZ | 0 | 1103 | 36.7667 | 3.0219 | 20.4857 | 18.4277 | 19.8622 | 22.1843 | 16.6210 | 17.0592 | 18.9478 | 2.0614 | high |
| TCGA-VS-A9UY | 1 | 555 | 18.5000 | 1.5205 | 18.3539 | 19.0027 | 20.8034 | 22.0424 | 21.4275 | 18.1078 | 19.1646 | 2.0477 | high |
| TCGA-DS-A1O9 | 1 | 266 | 8.8667 | 0.7288 | 19.5917 | 19.4243 | 19.8028 | 23.4124 | 18.6738 | 17.2434 | 17.9526 | 1.9817 | high |
| TCGA-EK-A2GZ | 0 | 383 | 12.7667 | 1.0493 | 20.0979 | 18.7051 | 20.7692 | 22.2106 | 20.3981 | 19.1069 | 20.5977 | 1.9797 | high |
| TCGA-VS-A9V0 | 0 | 573 | 19.1000 | 1.5699 | 19.2276 | 18.3460 | 19.1674 | 18.7029 | 18.5644 | 17.5092 | 14.3781 | 1.9769 | high |
| TCGA-C5-A7UC | 1 | 523 | 17.4333 | 1.4329 | 18.7261 | 19.7206 | 20.5843 | 17.8649 | 23.5858 | 15.8610 | 18.6587 | 1.9760 | high |
| TCGA-GH-A9DA | 0 | 540 | 18.0000 | 1.4795 | 19.4181 | 20.1201 | 21.1277 | 21.6839 | 19.9731 | 16.2333 | 19.4681 | 1.9717 | high |
| TCGA-ZJ-AAXJ | 0 | 0 | 0.0000 | 0.0000 | 19.8494 | 18.4333 | 20.7835 | 19.9216 | 20.2746 | 18.6507 | 19.6139 | 1.9462 | high |
| TCGA-MY-A913 | 0 | 524 | 17.4667 | 1.4356 | 19.0906 | 19.0517 | 20.4948 | 20.9248 | 20.2954 | 17.4387 | 18.9394 | 1.9222 | high |
| TCGA-VS-A9UJ | 1 | 52 | 1.7333 | 0.1425 | 19.4718 | 18.0457 | 20.1495 | 19.8189 | 18.3217 | 17.5322 | 18.7331 | 1.9098 | high |
| TCGA-EA-A4BA | 0 | 755 | 25.1667 | 2.0685 | 18.5072 | 18.0700 | 19.0134 | 19.7979 | 16.9840 | 16.8189 | 14.7833 | 1.9016 | high |
| TCGA-C5-A1M9 | 1 | 1065 | 35.5000 | 2.9178 | 19.2418 | 19.2824 | 21.5565 | 20.8745 | 18.7039 | 18.4665 | 18.0934 | 1.8812 | high |
| TCGA-HG-A2PA | 1 | 773 | 25.7667 | 2.1178 | 18.5156 | 18.4803 | 19.6009 | 22.4668 | 21.2365 | 17.6061 | 20.2519 | 1.8633 | high |
| TCGA-EK-A3GN | 0 | 27 | 0.9000 | 0.0740 | 19.5337 | 18.8273 | 20.0012 | 18.2204 | 21.3433 | 19.0816 | 15.8039 | 1.8465 | high |
| TCGA-VS-A9UM | 1 | 829 | 27.6333 | 2.2712 | 18.8032 | 18.6392 | 19.9087 | 21.7473 | 19.2349 | 18.2521 | 17.6924 | 1.8352 | high |
| TCGA-JX-A3PZ | 1 | 642 | 21.4000 | 1.7589 | 21.2193 | 19.9192 | 18.6274 | 21.4686 | 20.9873 | 17.1175 | 17.6322 | 1.8273 | high |
| TCGA-VS-A8QH | 1 | 1210 | 40.3333 | 3.3151 | 17.4813 | 16.9378 | 20.1717 | 19.1928 | 15.5424 | 15.9219 | 18.9363 | 1.7839 | high |
| TCGA-MA-AA43 | 0 | 346 | 11.5333 | 0.9479 | 18.9894 | 19.0187 | 19.7458 | 18.9713 | 21.4322 | 17.1675 | 17.7039 | 1.7758 | high |
| TCGA-C5-A2LT | 0 | 2226 | 74.2000 | 6.0986 | 18.5064 | 17.8413 | 20.3405 | 16.9896 | 19.7732 | 17.1526 | 18.3110 | 1.7705 | high |
| TCGA-JW-A5VG | 0 | 834 | 27.8000 | 2.2849 | 18.8384 | 18.6614 | 20.0987 | 20.2200 | 18.6427 | 16.9884 | 18.3802 | 1.7327 | high |
| TCGA-C5-A8XK | 0 | 3039 | 101.3000 | 8.3260 | 19.1581 | 19.3714 | 20.0291 | 19.9058 | 21.5084 | 18.3871 | 17.1391 | 1.7205 | high |
| TCGA-4J-AA1J | 0 | 542 | 18.0667 | 1.4849 | 17.9916 | 18.8975 | 20.5199 | 18.5303 | 21.6147 | 17.3532 | 18.2883 | 1.6825 | high |
| TCGA-VS-A9UL | 1 | 442 | 14.7333 | 1.2110 | 20.6441 | 18.6406 | 19.7494 | 18.2631 | 19.7377 | 19.6984 | 16.0843 | 1.6693 | high |
| TCGA-C5-A7CM | 0 | 619 | 20.6333 | 1.6959 | 18.3962 | 19.1787 | 21.0175 | 18.5094 | 18.8439 | 17.5400 | 16.4427 | 1.6679 | high |
| TCGA-C5-A1M6 | 1 | 955 | 31.8333 | 2.6164 | 20.6371 | 18.9208 | 18.8637 | 18.6718 | 21.7579 | 18.7690 | 16.9636 | 1.6515 | high |
| TCGA-VS-A8EB | 1 | 305 | 10.1667 | 0.8356 | 20.4404 | 20.2213 | 20.1699 | 19.6711 | 21.2230 | 18.6415 | 16.5632 | 1.6178 | high |
| TCGA-DS-A1OB | 1 | 861 | 28.7000 | 2.3589 | 17.9438 | 18.8823 | 20.3821 | 22.6737 | 18.1498 | 18.2292 | 18.1291 | 1.5789 | high |
| TCGA-C5-A1BM | 1 | 2520 | 84.0000 | 6.9041 | 17.8196 | 19.1085 | 20.1758 | 23.1220 | 19.1768 | 17.2220 | 19.7020 | 1.5541 | high |
| TCGA-C5-A1MN | 1 | 1245 | 41.5000 | 3.4110 | 18.9735 | 19.2319 | 20.3623 | 19.8535 | 20.8189 | 17.6974 | 19.1566 | 1.5491 | high |
| TCGA-VS-A950 | 0 | 1221 | 40.7000 | 3.3452 | 20.3670 | 19.4009 | 19.8877 | 19.7661 | 21.1476 | 19.3627 | 17.7442 | 1.5230 | high |
| TCGA-C5-A7CJ | 1 | 3097 | 103.2333 | 8.4849 | 17.4239 | 18.8369 | 20.1077 | 20.5220 | 20.7280 | 17.8858 | 18.2138 | 1.4899 | high |
| TCGA-HM-A4S6 | 0 | 454 | 15.1333 | 1.2438 | 20.0789 | 19.6451 | 20.6429 | 22.4916 | 16.6675 | 19.2402 | 17.1400 | 1.4861 | high |
| TCGA-C5-A7CH | 0 | 4694 | 156.4667 | 12.8603 | 19.0674 | 19.2421 | 19.5726 | 20.7473 | 20.4067 | 17.6305 | 18.6332 | 1.4642 | high |
| TCGA-EA-A50E | 1 | 227 | 7.5667 | 0.6219 | 18.3335 | 18.7841 | 19.0689 | 22.6707 | 20.7085 | 18.5512 | 18.6797 | 1.4640 | high |
| TCGA-2W-A8YY | 0 | 533 | 17.7667 | 1.4603 | 16.3840 | 17.7487 | 19.7029 | 21.9244 | 16.7273 | 17.5004 | 17.5176 | 1.4417 | high |
| TCGA-C5-A1MK | 1 | 74 | 2.4667 | 0.2027 | 18.6449 | 19.0569 | 19.8191 | 21.4349 | 20.5062 | 18.0791 | 19.3809 | 1.4319 | high |
| TCGA-C5-A3HE | 0 | 548 | 18.2667 | 1.5014 | 18.3184 | 18.6338 | 18.8094 | 19.8921 | 20.0296 | 17.9279 | 16.4262 | 1.4237 | high |
| TCGA-EA-A3HT | 0 | 954 | 31.8000 | 2.6137 | 19.8971 | 18.9770 | 19.2950 | 22.1386 | 20.2837 | 18.8902 | 19.7724 | 1.4016 | high |
| TCGA-EX-A69L | 0 | 602 | 20.0667 | 1.6493 | 20.1082 | 19.6590 | 19.7142 | 21.4361 | 19.2163 | 18.8634 | 17.5417 | 1.3875 | high |
| TCGA-ZJ-AB0I | 0 | 0 | 0.0000 | 0.0000 | 20.3040 | 19.7660 | 20.2808 | 21.5513 | 19.9511 | 18.3568 | 20.2061 | 1.3711 | high |
| TCGA-EK-A3GM | 0 | 0 | 0.0000 | 0.0000 | 18.5338 | 18.5296 | 19.0979 | 18.9108 | 18.3487 | 17.6011 | 15.9923 | 1.3608 | high |
| TCGA-EK-A2RM | 0 | 50 | 1.6667 | 0.1370 | 19.5427 | 19.7504 | 18.1054 | 23.0233 | 22.9315 | 19.0028 | 17.9096 | 1.3586 | high |
| TCGA-C5-A8XH | 1 | 1394 | 46.4667 | 3.8192 | 19.1831 | 19.5954 | 20.4855 | 19.8993 | 17.6652 | 17.4120 | 17.3575 | 1.3566 | high |
| TCGA-VS-A9UB | 0 | 911 | 30.3667 | 2.4959 | 20.2309 | 19.1412 | 19.7187 | 17.6785 | 19.4726 | 17.5306 | 17.9597 | 1.3403 | high |
| TCGA-VS-A953 | 1 | 477 | 15.9000 | 1.3068 | 19.5917 | 18.9790 | 20.3150 | 20.8089 | 20.0843 | 19.4771 | 19.6514 | 1.3314 | high |
| TCGA-EK-A2RN | 0 | 71 | 2.3667 | 0.1945 | 19.5003 | 18.4876 | 18.6014 | 22.9642 | 18.8233 | 17.7518 | 20.6899 | 1.3019 | high |
| TCGA-DG-A2KL | 0 | 1367 | 45.5667 | 3.7452 | 19.2312 | 18.3832 | 19.1165 | 20.8952 | 19.8358 | 18.2993 | 19.9429 | 1.3003 | high |
| TCGA-MA-AA3Y | 0 | 542 | 18.0667 | 1.4849 | 18.5903 | 19.6844 | 20.0256 | 18.8222 | 21.0054 | 16.0795 | 19.4070 | 1.2977 | high |
| TCGA-EA-A556 | 0 | 453 | 15.1000 | 1.2411 | 17.6813 | 17.7636 | 18.3648 | 20.2620 | 17.2604 | 17.9020 | 15.8624 | 1.2624 | high |
| TCGA-UC-A7PD | 1 | 355 | 11.8333 | 0.9726 | 18.9030 | 19.0453 | 19.3426 | 17.6099 | 21.0666 | 16.5773 | 18.7352 | 1.2506 | high |
| TCGA-C5-A907 | 0 | 448 | 14.9333 | 1.2274 | 19.3912 | 19.5461 | 19.8867 | 18.8631 | 19.8594 | 17.2663 | 18.5336 | 1.2074 | high |
| TCGA-LP-A4AX | 0 | 380 | 12.6667 | 1.0411 | 20.2701 | 19.1434 | 19.5388 | 17.7718 | 18.6968 | 18.0988 | 17.0231 | 1.2026 | high |
| TCGA-EA-A3Y4 | 0 | 1122 | 37.4000 | 3.0740 | 19.6548 | 19.7695 | 19.5724 | 17.7810 | 20.2878 | 17.0050 | 17.4081 | 1.1967 | high |
| TCGA-DS-A0VK | 1 | 1118 | 37.2667 | 3.0630 | 18.7694 | 19.8591 | 19.9835 | 23.2625 | 18.3469 | 18.6500 | 18.0690 | 1.1728 | high |
| TCGA-DS-A1OC | 0 | 376 | 12.5333 | 1.0301 | 19.3849 | 19.2866 | 21.0345 | 17.9715 | 19.8563 | 18.8081 | 18.9607 | 1.1669 | high |
| TCGA-JW-A5VJ | 0 | 652 | 21.7333 | 1.7863 | 17.2024 | 19.1364 | 20.6130 | 20.3439 | 19.5188 | 18.0282 | 18.4597 | 1.1643 | high |
| TCGA-VS-A8Q8 | 1 | 978 | 32.6000 | 2.6795 | 18.4859 | 19.1406 | 19.9889 | 20.2856 | 20.9415 | 18.1199 | 20.0199 | 1.1612 | high |
| TCGA-C5-A1ML | 1 | 636 | 21.2000 | 1.7425 | 19.1364 | 19.1569 | 20.3913 | 20.5915 | 17.3546 | 18.2445 | 18.6446 | 1.1599 | high |
| TCGA-ZJ-A8QR | 1 | 582 | 19.4000 | 1.5945 | 17.6419 | 19.7823 | 19.8110 | 19.1191 | 21.4376 | 17.2108 | 17.4004 | 1.1383 | high |
| TCGA-IR-A3LH | 0 | 2394 | 79.8000 | 6.5589 | 19.9547 | 19.6216 | 20.2442 | 22.4559 | 15.1942 | 18.2189 | 18.1246 | 1.1381 | high |
| TCGA-VS-A9U6 | 0 | 1320 | 44.0000 | 3.6164 | 20.3820 | 19.5784 | 20.7595 | 20.7551 | 17.2341 | 19.3968 | 18.5729 | 1.1362 | high |
| TCGA-EK-A2IP | 0 | 202 | 6.7333 | 0.5534 | 18.3837 | 19.6711 | 19.6146 | 16.5792 | 20.9589 | 17.1837 | 15.7825 | 1.1028 | high |
| TCGA-IR-A3LK | 1 | 908 | 30.2667 | 2.4877 | 17.5036 | 19.3801 | 20.0527 | 22.0437 | 18.9951 | 17.9820 | 18.7171 | 1.0691 | high |
| TCGA-JW-A5VH | 1 | 100 | 3.3333 | 0.2740 | 20.4290 | 18.4618 | 19.1336 | 18.5680 | 19.1814 | 18.1516 | 20.1623 | 1.0625 | high |
| TCGA-VS-A8EJ | 1 | 607 | 20.2333 | 1.6630 | 18.4849 | 19.0153 | 18.6912 | 21.3729 | 16.3076 | 17.9886 | 15.3285 | 1.0561 | high |
| TCGA-EA-A3QD | 0 | 397 | 13.2333 | 1.0877 | 17.9257 | 19.0133 | 19.1335 | 23.1227 | 15.8365 | 17.6027 | 17.2638 | 1.0434 | high |
| TCGA-DS-A7WI | 1 | 252 | 8.4000 | 0.6904 | 19.5387 | 20.2965 | 19.2360 | 20.4338 | 20.8766 | 17.5603 | 18.2012 | 1.0326 | high |
| TCGA-FU-A3HY | 0 | 954 | 31.8000 | 2.6137 | 17.7867 | 19.2688 | 20.6308 | 17.9740 | 20.8424 | 18.0699 | 18.5908 | 1.0293 | high |
| TCGA-DR-A0ZM | 0 | 1791 | 59.7000 | 4.9068 | 17.7841 | 18.1566 | 18.5481 | 20.7429 | 15.0495 | 16.3510 | 17.1489 | 1.0213 | high |
| TCGA-C5-A1MQ | 0 | 1031 | 34.3667 | 2.8247 | 17.8366 | 19.3367 | 17.9579 | 23.5842 | 18.9111 | 16.2811 | 18.8826 | 1.0133 | high |
| TCGA-C5-A1BQ | 1 | 604 | 20.1333 | 1.6548 | 19.8484 | 19.4955 | 20.6546 | 21.6434 | 14.4379 | 17.9150 | 18.9610 | 0.9916 | high |
| TCGA-C5-A2LV | 0 | 2234 | 74.4667 | 6.1205 | 20.9687 | 19.7134 | 20.1435 | 18.8379 | 17.2206 | 18.4567 | 18.0096 | 0.9869 | high |
| TCGA-C5-A1BN | 1 | 166 | 5.5333 | 0.4548 | 17.7744 | 19.3204 | 21.4566 | 15.3055 | 18.8936 | 16.9339 | 17.8855 | 0.9516 | high |
| TCGA-JW-A69B | 0 | 863 | 28.7667 | 2.3644 | 18.3963 | 19.6004 | 18.8611 | 23.5104 | 17.7268 | 18.1469 | 17.5688 | 0.9408 | high |
| TCGA-C5-A3HD | 0 | 1582 | 52.7333 | 4.3342 | 18.1611 | 19.4162 | 21.5560 | 17.2075 | 17.6535 | 18.0934 | 17.5602 | 0.9340 | high |
| TCGA-ZJ-AB0H | 0 | 0 | 0.0000 | 0.0000 | 17.0227 | 18.3624 | 19.4480 | 21.7090 | 18.2259 | 17.4765 | 20.1482 | 0.9193 | high |
| TCGA-FU-A5XV | 0 | 321 | 10.7000 | 0.8795 | 18.6599 | 19.0651 | 20.3922 | 17.2579 | 21.2228 | 17.7449 | 20.5935 | 0.9152 | high |
| TCGA-IR-A3LB | 1 | 2032 | 67.7333 | 5.5671 | 18.2360 | 18.1759 | 18.9530 | 19.5822 | 17.9646 | 17.4971 | 19.1312 | 0.9113 | high |
| TCGA-MA-AA3X | 0 | 617 | 20.5667 | 1.6904 | 18.0575 | 18.5054 | 18.3051 | 24.5616 | 18.1192 | 18.4675 | 20.0688 | 0.9104 | high |
| TCGA-LP-A4AW | 0 | 27 | 0.9000 | 0.0740 | 18.0068 | 18.2872 | 19.4268 | 17.9726 | 18.7566 | 17.7714 | 18.8313 | 0.8199 | high |
| TCGA-EK-A2RL | 1 | 1453 | 48.4333 | 3.9808 | 19.6911 | 19.1310 | 20.0121 | 18.8841 | 18.1802 | 18.9959 | 18.7034 | 0.8178 | high |
| TCGA-DS-A0VN | 0 | 3609 | 120.3000 | 9.8877 | 19.1456 | 18.9573 | 18.1714 | 20.6334 | 18.8411 | 17.5111 | 18.8165 | 0.8174 | high |
| TCGA-EK-A2PL | 0 | 13 | 0.4333 | 0.0356 | 18.3017 | 19.3009 | 20.5107 | 17.6705 | 17.9808 | 17.4384 | 18.2475 | 0.7971 | high |
| TCGA-C5-A8YQ | 1 | 715 | 23.8333 | 1.9589 | 17.8508 | 18.8088 | 20.0108 | 21.9611 | 14.3622 | 18.8711 | 16.8883 | 0.7833 | high |
| TCGA-EK-A2PK | 0 | 12 | 0.4000 | 0.0329 | 17.0625 | 18.5799 | 18.9615 | 19.2203 | 19.5337 | 16.7165 | 19.2079 | 0.7822 | high |
| TCGA-ZJ-AAX8 | 0 | 0 | 0.0000 | 0.0000 | 18.8621 | 19.9869 | 19.3973 | 20.6186 | 20.8087 | 18.5145 | 18.7628 | 0.7647 | high |
| TCGA-C5-A1MF | 0 | 1617 | 53.9000 | 4.4301 | 19.8238 | 19.8954 | 18.7959 | 20.9468 | 16.7021 | 18.5590 | 15.7308 | 0.7590 | high |
| TCGA-VS-A8EH | 0 | 986 | 32.8667 | 2.7014 | 19.1485 | 19.2886 | 19.9591 | 22.3835 | 16.1011 | 18.5117 | 19.8127 | 0.7499 | high |
| TCGA-EK-A2RE | 0 | 57 | 1.9000 | 0.1562 | 17.6339 | 19.2508 | 19.6289 | 20.7503 | 20.2409 | 17.2856 | 20.9595 | 0.7486 | high |
| TCGA-C5-A1MH | 1 | 1186 | 39.5333 | 3.2493 | 18.6389 | 19.3485 | 18.4768 | 23.0319 | 19.4512 | 18.0234 | 20.1281 | 0.7442 | high |
| TCGA-HM-A3JJ | 1 | 659 | 21.9667 | 1.8055 | 17.9118 | 18.7500 | 19.1583 | 18.0414 | 17.4307 | 17.7232 | 16.2382 | 0.7402 | high |
| TCGA-EA-A439 | 0 | 965 | 32.1667 | 2.6438 | 20.1997 | 18.8597 | 19.2930 | 19.5561 | 17.0528 | 19.0664 | 18.6424 | 0.7362 | high |
| TCGA-ZJ-AAXU | 0 | 5 | 0.1667 | 0.0137 | 19.1013 | 19.8132 | 19.6733 | 19.9236 | 20.5146 | 18.5490 | 19.3749 | 0.7323 | high |
| TCGA-UC-A7PG | 1 | 370 | 12.3333 | 1.0137 | 18.0875 | 18.4050 | 18.6714 | 21.1715 | 18.9963 | 18.2415 | 20.2315 | 0.7014 | high |
| TCGA-VS-A8QA | 0 | 1099 | 36.6333 | 3.0110 | 20.7977 | 19.5779 | 19.0294 | 18.5033 | 20.2809 | 20.2532 | 17.4284 | 0.6968 | high |
| TCGA-EA-A6QX | 0 | 730 | 24.3333 | 2.0000 | 17.6399 | 19.8397 | 19.3159 | 21.6293 | 18.6755 | 18.1063 | 17.3349 | 0.6876 | high |
| TCGA-EA-A3HR | 0 | 940 | 31.3333 | 2.5753 | 18.5793 | 19.8740 | 20.2237 | 19.7791 | 19.9823 | 18.2068 | 19.7465 | 0.6831 | high |
| TCGA-VS-A9V1 | 1 | 157 | 5.2333 | 0.4301 | 19.1256 | 18.7331 | 17.7217 | 18.4340 | 21.3056 | 18.1199 | 18.5864 | 0.6773 | high |
| TCGA-EA-A411 | 0 | 747 | 24.9000 | 2.0466 | 18.0872 | 18.8903 | 20.0331 | 19.2050 | 16.5184 | 17.9678 | 18.2186 | 0.6698 | high |
| TCGA-C5-A7CK | 1 | 4086 | 136.2000 | 11.1945 | 17.6140 | 18.7958 | 20.1113 | 20.8274 | 17.8499 | 18.5404 | 19.9321 | 0.6653 | high |
| TCGA-EA-A3QE | 0 | 761 | 25.3667 | 2.0849 | 18.3448 | 18.9098 | 19.3223 | 19.4416 | 18.9907 | 17.5480 | 20.0714 | 0.6595 | high |
| TCGA-EA-A1QS | 0 | 1203 | 40.1000 | 3.2959 | 20.1688 | 19.1988 | 19.9705 | 17.6804 | 15.7967 | 18.3414 | 17.6400 | 0.6509 | high |
| TCGA-ZJ-AAXT | 0 | 0 | 0.0000 | 0.0000 | 17.3411 | 19.8809 | 19.9203 | 19.7843 | 20.1406 | 17.3749 | 18.8469 | 0.6478 | high |
| TCGA-EK-A3GK | 0 | 15 | 0.5000 | 0.0411 | 17.7616 | 18.9650 | 20.2254 | 18.6847 | 17.6014 | 17.2428 | 19.5400 | 0.6453 | high |
| TCGA-EA-A3HQ | 0 | 1136 | 37.8667 | 3.1123 | 18.3123 | 19.5834 | 19.5142 | 22.7701 | 17.2198 | 19.0999 | 18.0897 | 0.6383 | high |
| TCGA-DS-A5RQ | 0 | 512 | 17.0667 | 1.4027 | 18.5865 | 19.2476 | 19.2780 | 21.0413 | 18.0048 | 18.2611 | 19.1563 | 0.6342 | high |
| TCGA-VS-A957 | 0 | 1688 | 56.2667 | 4.6247 | 19.2348 | 19.5127 | 20.0159 | 20.8452 | 17.3204 | 18.8732 | 19.3128 | 0.6186 | high |
| TCGA-DS-A7WH | 0 | 533 | 17.7667 | 1.4603 | 18.7802 | 18.9258 | 19.1398 | 17.2336 | 21.5674 | 18.6220 | 19.3027 | 0.6144 | high |
| TCGA-JW-A5VL | 0 | 474 | 15.8000 | 1.2986 | 18.8121 | 20.0876 | 19.1326 | 20.4679 | 19.1537 | 17.9050 | 17.9233 | 0.6081 | high |
| TCGA-EK-A2PI | 0 | 586 | 19.5333 | 1.6055 | 19.1717 | 19.7242 | 19.8384 | 21.3370 | 17.2419 | 18.7398 | 18.9149 | 0.6030 | high |
| TCGA-DG-A2KK | 0 | 2496 | 83.2000 | 6.8384 | 18.6302 | 19.8647 | 18.8658 | 20.9908 | 21.3056 | 18.4981 | 19.4136 | 0.5950 | high |
| TCGA-Q1-A73Q | 0 | 284 | 9.4667 | 0.7781 | 18.3036 | 19.1513 | 20.4436 | 19.0720 | 19.1250 | 18.1613 | 21.0450 | 0.5810 | high |
| TCGA-EX-A8YF | 0 | 473 | 15.7667 | 1.2959 | 15.2474 | 18.3872 | 20.5701 | 17.3481 | 17.1997 | 17.7615 | 16.5412 | 0.5707 | high |
| TCGA-IR-A3LI | 0 | 2493 | 83.1000 | 6.8301 | 20.7055 | 19.2767 | 19.6792 | 16.7243 | 18.8851 | 18.4542 | 19.7340 | 0.5634 | high |
| TCGA-FU-A770 | 0 | 34 | 1.1333 | 0.0932 | 17.6245 | 19.3803 | 20.1632 | 17.7369 | 22.0083 | 18.3567 | 20.0509 | 0.5632 | high |
| TCGA-EX-A69M | 1 | 253 | 8.4333 | 0.6932 | 18.9245 | 19.4399 | 19.0958 | 20.0519 | 18.5857 | 17.7855 | 19.3373 | 0.5614 | high |
| TCGA-C5-A7X5 | 1 | 414 | 13.8000 | 1.1342 | 19.0240 | 19.3009 | 20.2490 | 20.5890 | 15.0980 | 18.8031 | 18.3232 | 0.5564 | high |
| TCGA-VS-A9UH | 0 | 1427 | 47.5667 | 3.9096 | 18.9881 | 19.8425 | 17.8923 | 23.2974 | 16.8646 | 17.1752 | 18.1962 | 0.5430 | high |
| TCGA-C5-A901 | 0 | 518 | 17.2667 | 1.4192 | 18.3848 | 19.0975 | 18.4483 | 22.0614 | 19.9560 | 18.3601 | 20.6255 | 0.5413 | high |
| TCGA-VS-A8QC | 1 | 350 | 11.6667 | 0.9589 | 18.7762 | 19.2073 | 19.5162 | 20.2784 | 17.4364 | 18.4205 | 19.1295 | 0.5370 | high |
| TCGA-C5-A1MP | 0 | 109 | 3.6333 | 0.2986 | 17.5543 | 19.7384 | 19.3598 | 22.3226 | 18.8880 | 18.7045 | 18.4549 | 0.5341 | high |
| TCGA-ZJ-AAXI | 0 | 0 | 0.0000 | 0.0000 | 20.0591 | 18.4555 | 19.0852 | 16.7393 | 15.3481 | 18.9272 | 16.5061 | 0.5001 | high |
| TCGA-BI-A0VS | 0 | 1735 | 57.8333 | 4.7534 | 18.4058 | 19.5117 | 19.7582 | 21.3337 | 15.6965 | 18.3819 | 17.9895 | 0.4940 | high |
| TCGA-VS-A94W | 0 | 1243 | 41.4333 | 3.4055 | 19.4070 | 19.7439 | 19.4970 | 21.0993 | 16.7424 | 19.4421 | 17.4930 | 0.4896 | high |
| TCGA-EA-A5O9 | 0 | 788 | 26.2667 | 2.1589 | 19.1999 | 20.0047 | 19.4303 | 19.7133 | 18.3644 | 18.0841 | 18.3527 | 0.4820 | high |
| TCGA-DS-A7WF | 1 | 492 | 16.4000 | 1.3479 | 19.2828 | 19.8380 | 19.4050 | 18.6202 | 18.0319 | 18.0548 | 17.7070 | 0.4652 | high |
| TCGA-LP-A4AU | 0 | 343 | 11.4333 | 0.9397 | 17.6223 | 18.8545 | 19.2850 | 16.6885 | 19.7566 | 16.6364 | 19.5737 | 0.4637 | high |
| TCGA-VS-A8EC | 0 | 1415 | 47.1667 | 3.8767 | 18.6337 | 19.2592 | 20.2852 | 17.9537 | 18.1199 | 19.2445 | 18.2406 | 0.4447 | high |
| TCGA-C5-A7XC | 0 | 1551 | 51.7000 | 4.2493 | 18.2940 | 18.7015 | 18.5340 | 19.2379 | 17.2946 | 17.9951 | 17.8543 | 0.4406 | high |
| TCGA-BI-A0VR | 0 | 1505 | 50.1667 | 4.1233 | 18.6872 | 19.2696 | 18.8283 | 21.7253 | 18.9951 | 18.9988 | 19.9043 | 0.4358 | high |
| TCGA-VS-A9UT | 0 | 482 | 16.0667 | 1.3205 | 17.3352 | 17.6644 | 17.6908 | 19.1446 | 15.8679 | 18.2257 | 16.1057 | 0.4275 | high |
| TCGA-R2-A69V | 0 | 596 | 19.8667 | 1.6329 | 17.9381 | 18.5492 | 18.9616 | 19.1442 | 16.9382 | 17.1435 | 19.5860 | 0.4083 | high |
| TCGA-LP-A5U3 | 0 | 25 | 0.8333 | 0.0685 | 18.7080 | 19.6051 | 19.7133 | 21.3281 | 16.2344 | 18.7430 | 18.5460 | 0.4007 | high |
| TCGA-C5-A1M7 | 0 | 1409 | 46.9667 | 3.8603 | 18.6475 | 19.1018 | 19.2475 | 15.7456 | 21.5807 | 17.9603 | 19.5736 | 0.3932 | high |
| TCGA-C5-A1MJ | 1 | 14 | 0.4667 | 0.0384 | 17.9033 | 18.8122 | 18.9599 | 20.3655 | 17.0773 | 18.0651 | 18.9269 | 0.3887 | high |
| TCGA-EA-A5ZF | 0 | 828 | 27.6000 | 2.2685 | 19.4042 | 19.7799 | 18.5304 | 20.9090 | 18.3584 | 19.1654 | 17.5854 | 0.3843 | low |
| TCGA-C5-A8XI | 0 | 254 | 8.4667 | 0.6959 | 18.9825 | 19.7077 | 19.4103 | 18.2577 | 20.3148 | 18.0062 | 19.8635 | 0.3839 | low |
| TCGA-ZJ-AAX4 | 0 | 21 | 0.7000 | 0.0575 | 16.4301 | 19.0868 | 18.9861 | 23.4087 | 15.9411 | 17.5685 | 18.7377 | 0.3740 | low |
| TCGA-FU-A3TQ | 0 | 795 | 26.5000 | 2.1781 | 18.4157 | 19.5552 | 19.5354 | 17.5339 | 20.8764 | 17.4525 | 20.3950 | 0.3736 | low |
| TCGA-EA-A97N | 0 | 11 | 0.3667 | 0.0301 | 18.5519 | 18.7985 | 20.2142 | 18.4047 | 16.5873 | 18.3198 | 19.9525 | 0.3727 | low |
| TCGA-DS-A0VM | 0 | 3589 | 119.6333 | 9.8329 | 18.3347 | 19.5591 | 19.6368 | 19.7589 | 16.6701 | 17.9561 | 18.1503 | 0.3714 | low |
| TCGA-WL-A834 | 0 | 791 | 26.3667 | 2.1671 | 18.4893 | 19.0518 | 20.3798 | 17.7449 | 16.7471 | 18.5105 | 18.8001 | 0.3689 | low |
| TCGA-JX-A5QV | 0 | 636 | 21.2000 | 1.7425 | 18.5662 | 19.3770 | 19.2714 | 21.1230 | 18.4386 | 18.7212 | 20.0627 | 0.3646 | low |
| TCGA-EK-A2PM | 0 | 18 | 0.6000 | 0.0493 | 18.0366 | 18.3467 | 19.4688 | 18.2129 | 16.1674 | 18.0002 | 18.8963 | 0.3630 | low |
| TCGA-MY-A5BF | 0 | 634 | 21.1333 | 1.7370 | 18.9871 | 19.6153 | 19.4856 | 21.6654 | 17.3665 | 18.4835 | 20.3509 | 0.3527 | low |
| TCGA-VS-A9UI | 1 | 1372 | 45.7333 | 3.7589 | 17.8112 | 19.3264 | 18.4498 | 21.8839 | 17.4046 | 17.7785 | 18.6526 | 0.3389 | low |
| TCGA-C5-A1BI | 0 | 1112 | 37.0667 | 3.0466 | 18.1947 | 19.0945 | 18.8504 | 16.9425 | 20.5045 | 18.1498 | 18.3868 | 0.3311 | low |
| TCGA-DS-A0VL | 0 | 81 | 2.7000 | 0.2219 | 18.9165 | 19.8552 | 20.1141 | 18.9402 | 19.7003 | 18.7099 | 20.2659 | 0.3281 | low |
| TCGA-VS-A8QF | 0 | 1800 | 60.0000 | 4.9315 | 17.4239 | 19.7196 | 18.4843 | 22.3206 | 18.2813 | 17.6593 | 18.4946 | 0.3275 | low |
| TCGA-ZJ-A8QQ | 0 | 2056 | 68.5333 | 5.6329 | 17.1522 | 19.8314 | 20.3683 | 20.0122 | 17.8571 | 17.4433 | 19.5088 | 0.3237 | low |
| TCGA-EA-A5ZD | 0 | 830 | 27.6667 | 2.2740 | 18.6244 | 19.6667 | 19.5823 | 19.0237 | 20.2793 | 19.1638 | 19.3384 | 0.3167 | low |
| TCGA-MA-AA3W | 0 | 685 | 22.8333 | 1.8767 | 17.4132 | 18.7932 | 20.5566 | 16.6499 | 17.3164 | 17.8723 | 19.0542 | 0.3141 | low |
| TCGA-VS-A8EK | 1 | 829 | 27.6333 | 2.2712 | 17.7518 | 19.0704 | 18.4902 | 18.6464 | 20.4528 | 18.4690 | 18.2992 | 0.3133 | low |
| TCGA-VS-A9V3 | 0 | 540 | 18.0000 | 1.4795 | 17.6379 | 19.0115 | 19.2331 | 21.3027 | 15.5947 | 18.4634 | 18.0050 | 0.3125 | low |
| TCGA-ZJ-AAXD | 0 | 0 | 0.0000 | 0.0000 | 18.9392 | 18.8011 | 19.1124 | 18.5426 | 19.6741 | 18.9531 | 20.6024 | 0.3012 | low |
| TCGA-FU-A2QG | 0 | 579 | 19.3000 | 1.5863 | 17.8754 | 19.1567 | 19.6488 | 17.4196 | 18.6364 | 17.4368 | 19.3628 | 0.2944 | low |
| TCGA-DS-A1OA | 1 | 879 | 29.3000 | 2.4082 | 17.9594 | 19.3030 | 19.6697 | 20.2240 | 16.0802 | 18.5061 | 18.0339 | 0.2900 | low |
| TCGA-VS-A9UC | 0 | 825 | 27.5000 | 2.2603 | 17.5634 | 19.1042 | 20.0228 | 19.7888 | 15.4301 | 17.5554 | 19.0797 | 0.2878 | low |
| TCGA-Q1-A73P | 0 | 483 | 16.1000 | 1.3233 | 17.4778 | 18.9845 | 19.9059 | 18.5311 | 18.3180 | 18.1088 | 19.7166 | 0.2872 | low |
| TCGA-VS-A8EG | 0 | 1386 | 46.2000 | 3.7973 | 19.5776 | 19.6587 | 20.0538 | 17.2478 | 19.6607 | 18.6660 | 20.3310 | 0.2780 | low |
| TCGA-EK-A2RA | 0 | 1246 | 41.5333 | 3.4137 | 18.8635 | 19.6245 | 19.1516 | 20.2082 | 17.7312 | 18.8136 | 18.4541 | 0.2694 | low |
| TCGA-FU-A40J | 0 | 426 | 14.2000 | 1.1671 | 17.1670 | 19.1355 | 19.7763 | 18.0720 | 18.6291 | 18.4662 | 18.1184 | 0.2607 | low |
| TCGA-Q1-A73R | 0 | 567 | 18.9000 | 1.5534 | 18.9453 | 18.9846 | 19.5230 | 16.6264 | 18.3005 | 18.4511 | 18.8713 | 0.2570 | low |
| TCGA-C5-A8XJ | 0 | 4467 | 148.9000 | 12.2384 | 18.3476 | 19.0843 | 20.4271 | 19.1654 | 14.5218 | 18.5199 | 18.6138 | 0.2448 | low |
| TCGA-EK-A3GJ | 0 | 3 | 0.1000 | 0.0082 | 15.6404 | 19.8702 | 18.7184 | 22.3957 | 19.3876 | 15.7715 | 20.1655 | 0.2380 | low |
| TCGA-VS-A9V5 | 1 | 494 | 16.4667 | 1.3534 | 17.3810 | 19.1908 | 19.6124 | 18.4616 | 17.9397 | 18.0814 | 18.3310 | 0.2331 | low |
| TCGA-C5-A1BL | 0 | 5271 | 175.7000 | 14.4411 | 18.7268 | 18.8010 | 19.8529 | 18.0957 | 17.2232 | 18.4462 | 20.3247 | 0.2295 | low |
| TCGA-EK-A2H0 | 0 | 1847 | 61.5667 | 5.0603 | 17.0292 | 19.5302 | 20.3705 | 20.0083 | 16.6867 | 17.7169 | 19.2936 | 0.2293 | low |
| TCGA-Q1-A6DW | 0 | 534 | 17.8000 | 1.4630 | 19.1199 | 19.3180 | 19.0905 | 19.2379 | 19.5743 | 19.2099 | 19.9670 | 0.2283 | low |
| TCGA-VS-A9UQ | 0 | 1263 | 42.1000 | 3.4603 | 18.0666 | 19.2559 | 20.6619 | 17.3565 | 16.3573 | 18.1595 | 18.7739 | 0.2259 | low |
| TCGA-JX-A3Q0 | 0 | 6375 | 212.5000 | 17.4658 | 18.9123 | 19.6466 | 18.7461 | 22.3496 | 16.0368 | 18.1423 | 19.3188 | 0.2092 | low |
| TCGA-C5-A3HF | 1 | 543 | 18.1000 | 1.4877 | 17.2323 | 19.2109 | 19.9016 | 17.6560 | 18.3027 | 17.8716 | 18.6970 | 0.2018 | low |
| TCGA-C5-A3HL | 0 | 621 | 20.7000 | 1.7014 | 20.8773 | 19.8901 | 19.8073 | 18.3932 | 19.4319 | 19.4563 | 21.1785 | 0.2005 | low |
| TCGA-MU-A8JM | 0 | 607 | 20.2333 | 1.6630 | 20.0465 | 20.0564 | 19.7959 | 18.1186 | 19.3513 | 19.1367 | 19.6746 | 0.1963 | low |
| TCGA-DR-A0ZL | 0 | 2669 | 88.9667 | 7.3123 | 17.2260 | 19.3243 | 19.9109 | 18.7169 | 19.2575 | 17.9680 | 20.1794 | 0.1848 | low |
| TCGA-VS-A959 | 0 | 1561 | 52.0333 | 4.2767 | 17.1686 | 18.9376 | 19.1660 | 18.7923 | 18.1514 | 18.7240 | 17.7446 | 0.1805 | low |
| TCGA-ZJ-A8QO | 0 | 0 | 0.0000 | 0.0000 | 17.0581 | 19.3937 | 19.4381 | 21.1356 | 18.9946 | 18.8248 | 19.6643 | 0.1792 | low |
| TCGA-C5-A2M2 | 1 | 1011 | 33.7000 | 2.7699 | 18.9456 | 19.0194 | 18.8426 | 18.1970 | 18.9825 | 18.2754 | 20.1219 | 0.1711 | low |
| TCGA-IR-A3LC | 0 | 3935 | 131.1667 | 10.7808 | 18.4867 | 19.1770 | 19.2512 | 19.2568 | 17.1389 | 19.0218 | 18.3188 | 0.1577 | low |
| TCGA-FU-A23L | 0 | 725 | 24.1667 | 1.9863 | 17.1176 | 19.1447 | 20.3797 | 17.4509 | 17.0021 | 18.3274 | 18.0419 | 0.1506 | low |
| TCGA-C5-A2LS | 0 | 1345 | 44.8333 | 3.6849 | 17.9448 | 19.0363 | 19.5946 | 15.7683 | 20.9587 | 19.0373 | 18.9983 | 0.1251 | low |
| TCGA-PN-A8MA | 0 | 90 | 3.0000 | 0.2466 | 18.6338 | 19.1748 | 19.5988 | 18.6586 | 15.7166 | 19.0704 | 17.5644 | 0.1217 | low |
| TCGA-EK-A2RC | 0 | 129 | 4.3000 | 0.3534 | 17.6675 | 19.3254 | 18.7806 | 20.9761 | 16.6645 | 17.8890 | 18.6928 | 0.1195 | low |
| TCGA-MA-AA41 | 0 | 279 | 9.3000 | 0.7644 | 17.2586 | 19.5917 | 19.5811 | 19.4422 | 16.8578 | 18.0189 | 17.6323 | 0.1182 | low |
| TCGA-C5-A8YT | 1 | 633 | 21.1000 | 1.7342 | 16.6371 | 17.9640 | 18.7862 | 17.1746 | 14.1151 | 17.1318 | 16.3747 | 0.1126 | low |
| TCGA-C5-A1BJ | 0 | 4385 | 146.1667 | 12.0137 | 18.4720 | 19.4972 | 19.8371 | 16.4235 | 21.5108 | 18.5250 | 20.7906 | 0.1108 | low |
| TCGA-VS-A8Q9 | 0 | 1630 | 54.3333 | 4.4658 | 20.4727 | 19.9963 | 19.2592 | 17.0401 | 16.6299 | 18.5438 | 17.2051 | 0.1036 | low |
| TCGA-VS-A9UR | 0 | 793 | 26.4333 | 2.1726 | 19.3065 | 19.4840 | 20.1304 | 15.8821 | 18.2356 | 18.3272 | 19.4848 | 0.1029 | low |
| TCGA-DG-A2KH | 0 | 34 | 1.1333 | 0.0932 | 18.4284 | 19.2144 | 19.7649 | 17.7372 | 17.7932 | 18.5148 | 19.3483 | 0.1028 | low |
| TCGA-EA-A78R | 0 | 410 | 13.6667 | 1.1233 | 19.1317 | 19.8961 | 18.4175 | 19.2290 | 19.4689 | 17.7940 | 19.7420 | 0.0480 | low |
| TCGA-EK-A2RO | 0 | 2 | 0.0667 | 0.0055 | 19.7153 | 20.4907 | 19.1166 | 20.2906 | 19.5257 | 18.0976 | 20.9727 | 0.0444 | low |
| TCGA-Q1-A5R3 | 0 | 485 | 16.1667 | 1.3288 | 16.7133 | 19.6927 | 19.6254 | 17.3362 | 17.2746 | 15.8393 | 18.4301 | 0.0440 | low |
| TCGA-Q1-A5R1 | 0 | 474 | 15.8000 | 1.2986 | 16.9102 | 18.2186 | 18.3430 | 18.0365 | 19.9151 | 18.2519 | 20.0719 | 0.0183 | low |
| TCGA-VS-A958 | 0 | 1525 | 50.8333 | 4.1781 | 17.8484 | 19.6285 | 19.2437 | 17.3820 | 19.1606 | 17.1179 | 19.5235 | 0.0183 | low |
| TCGA-ZX-AA5X | 0 | 119 | 3.9667 | 0.3260 | 16.7646 | 18.8949 | 19.1925 | 18.1903 | 17.4749 | 17.6093 | 18.5989 | 0.0144 | low |
| TCGA-UC-A7PI | 0 | 2114 | 70.4667 | 5.7918 | 17.2474 | 19.4969 | 19.6558 | 19.0295 | 17.3366 | 17.6039 | 19.2840 | -0.0017 | low |
| TCGA-EA-A410 | 0 | 803 | 26.7667 | 2.2000 | 16.9555 | 18.7922 | 19.2506 | 19.5340 | 15.3650 | 17.9536 | 18.3216 | -0.0117 | low |
| TCGA-VS-A8QM | 1 | 951 | 31.7000 | 2.6055 | 18.0941 | 18.7667 | 18.1852 | 18.4035 | 18.0400 | 17.8254 | 19.2940 | -0.0201 | low |
| TCGA-FU-A3YQ | 0 | 861 | 28.7000 | 2.3589 | 17.9651 | 19.4126 | 19.0797 | 18.0087 | 19.1974 | 18.4611 | 19.0072 | -0.0261 | low |
| TCGA-C5-A1BE | 1 | 2094 | 69.8000 | 5.7370 | 18.1041 | 19.4469 | 19.5594 | 20.3487 | 17.1166 | 17.6701 | 21.4983 | -0.0318 | low |
| TCGA-EA-A43B | 0 | 791 | 26.3667 | 2.1671 | 18.8473 | 19.9799 | 18.8283 | 16.5266 | 17.2807 | 16.7356 | 17.3832 | -0.0324 | low |
| TCGA-JX-A3Q8 | 0 | 1357 | 45.2333 | 3.7178 | 18.8361 | 19.1415 | 18.4599 | 17.3032 | 18.7796 | 18.3471 | 18.8578 | -0.0351 | low |
| TCGA-C5-A7UI | 1 | 2888 | 96.2667 | 7.9123 | 18.6308 | 19.8019 | 17.5688 | 19.9284 | 18.7357 | 18.0042 | 17.7867 | -0.0457 | low |
| TCGA-LP-A4AV | 0 | 0 | 0.0000 | 0.0000 | 18.0788 | 20.7764 | 18.6526 | 20.3972 | 17.1878 | 16.5383 | 17.6187 | -0.0554 | low |
| TCGA-EA-A3HU | 0 | 1013 | 33.7667 | 2.7753 | 18.6863 | 19.7244 | 18.6738 | 20.5645 | 14.6923 | 17.9866 | 17.5149 | -0.0680 | low |
| TCGA-C5-A2LZ | 1 | 3046 | 101.5333 | 8.3452 | 18.7295 | 19.5562 | 18.9161 | 19.4482 | 18.1279 | 18.7542 | 19.5618 | -0.0731 | low |
| TCGA-Q1-A6DV | 0 | 491 | 16.3667 | 1.3452 | 16.9069 | 19.3953 | 19.6226 | 15.7999 | 22.9869 | 18.4124 | 20.4159 | -0.0825 | low |
| TCGA-VS-A9U5 | 0 | 1535 | 51.1667 | 4.2055 | 17.6907 | 19.4375 | 19.7521 | 17.8060 | 18.8744 | 18.5149 | 19.7156 | -0.0829 | low |
| TCGA-EA-A3HS | 0 | 959 | 31.9667 | 2.6274 | 18.6003 | 19.3760 | 19.1673 | 21.8693 | 14.8272 | 19.1818 | 19.2858 | -0.0984 | low |
| TCGA-C5-A902 | 0 | 149 | 4.9667 | 0.4082 | 19.1663 | 19.8553 | 19.5083 | 18.4380 | 16.8566 | 18.5963 | 18.8628 | -0.1026 | low |
| TCGA-EA-A5ZE | 0 | 829 | 27.6333 | 2.2712 | 19.3760 | 19.7792 | 19.0963 | 18.5146 | 15.0486 | 18.6318 | 16.9824 | -0.1118 | low |
| TCGA-Q1-A73S | 0 | 688 | 22.9333 | 1.8849 | 15.9387 | 19.7132 | 19.2091 | 20.7050 | 16.4262 | 17.2621 | 17.7773 | -0.1161 | low |
| TCGA-EA-A44S | 0 | 369 | 12.3000 | 1.0110 | 17.6758 | 18.8697 | 18.2562 | 20.7545 | 15.4646 | 17.9703 | 18.8299 | -0.1224 | low |
| TCGA-VS-A8EI | 0 | 729 | 24.3000 | 1.9973 | 16.7416 | 18.4782 | 18.9141 | 18.1049 | 18.6628 | 18.4828 | 19.7236 | -0.1256 | low |
| TCGA-MA-AA3Z | 0 | 595 | 19.8333 | 1.6301 | 17.7644 | 19.7493 | 19.8696 | 20.6790 | 16.3373 | 17.9864 | 20.6582 | -0.1491 | low |
| TCGA-EK-A2IR | 0 | 3442 | 114.7333 | 9.4301 | 18.0292 | 20.3251 | 20.5185 | 19.0046 | 16.2909 | 18.4678 | 18.5472 | -0.1532 | low |
| TCGA-Q1-A5R2 | 0 | 499 | 16.6333 | 1.3671 | 17.1166 | 19.6184 | 17.6654 | 22.6080 | 17.3548 | 18.1079 | 18.1663 | -0.1622 | low |
| TCGA-VS-A9UD | 0 | 739 | 24.6333 | 2.0247 | 19.0975 | 20.1228 | 17.0072 | 21.3159 | 17.8371 | 17.4439 | 18.2783 | -0.1704 | low |
| TCGA-FU-A3WB | 0 | 491 | 16.3667 | 1.3452 | 18.5490 | 20.1438 | 19.8905 | 17.6506 | 16.9620 | 18.9207 | 17.3520 | -0.1797 | low |
| TCGA-VS-A94Z | 0 | 1015 | 33.8333 | 2.7808 | 18.0821 | 19.7228 | 19.7046 | 18.4318 | 17.1996 | 18.6692 | 18.9909 | -0.2202 | low |
| TCGA-EA-A5FO | 0 | 815 | 27.1667 | 2.2329 | 19.7242 | 19.5977 | 20.2301 | 14.7337 | 19.1264 | 19.4022 | 20.0084 | -0.2227 | low |
| TCGA-IR-A3LF | 0 | 2949 | 98.3000 | 8.0795 | 16.9096 | 19.1219 | 19.0450 | 17.9239 | 17.2852 | 18.0776 | 18.1788 | -0.2360 | low |
| TCGA-FU-A23K | 0 | 372 | 12.4000 | 1.0192 | 17.6967 | 19.0202 | 17.1926 | 17.6877 | 16.1716 | 17.4210 | 15.3985 | -0.2389 | low |
| TCGA-EK-A2R7 | 0 | 27 | 0.9000 | 0.0740 | 18.0114 | 19.0567 | 19.3334 | 16.4934 | 17.0887 | 19.0505 | 17.7841 | -0.2558 | low |
| TCGA-EX-A449 | 0 | 447 | 14.9000 | 1.2247 | 17.8398 | 19.0376 | 18.3909 | 18.3564 | 17.2659 | 18.2584 | 18.5910 | -0.2567 | low |
| TCGA-VS-A8EL | 0 | 1992 | 66.4000 | 5.4575 | 17.6104 | 19.5853 | 17.8204 | 20.6460 | 16.9283 | 17.4255 | 18.5435 | -0.2662 | low |
| TCGA-FU-A3TX | 0 | 45 | 1.5000 | 0.1233 | 17.4627 | 20.1840 | 18.9219 | 20.2398 | 17.5075 | 18.7127 | 17.3913 | -0.2708 | low |
| TCGA-ZJ-AAXB | 0 | 0 | 0.0000 | 0.0000 | 18.5730 | 20.1544 | 18.6093 | 18.4408 | 20.1433 | 18.6331 | 19.1699 | -0.2779 | low |
| TCGA-C5-A1ME | 0 | 1756 | 58.5333 | 4.8110 | 16.0931 | 19.2865 | 17.9793 | 19.1179 | 18.6787 | 16.8863 | 18.5825 | -0.2842 | low |
| TCGA-EK-A2PG | 0 | 46 | 1.5333 | 0.1260 | 15.4733 | 19.3511 | 18.8899 | 20.5803 | 18.9607 | 18.8412 | 18.5447 | -0.3061 | low |
| TCGA-VS-A9UO | 0 | 1456 | 48.5333 | 3.9890 | 17.5112 | 19.0563 | 19.0780 | 15.0066 | 17.3547 | 17.8174 | 17.3971 | -0.3144 | low |
| TCGA-C5-A7UH | 0 | 3988 | 132.9333 | 10.9260 | 15.1200 | 19.7647 | 19.0430 | 18.0745 | 19.9240 | 17.0449 | 18.3124 | -0.3345 | low |
| TCGA-UC-A7PF | 1 | 2859 | 95.3000 | 7.8329 | 18.2140 | 19.0286 | 20.1142 | 19.0577 | 13.0631 | 18.7381 | 19.5539 | -0.3627 | low |
| TCGA-VS-A9U7 | 0 | 1472 | 49.0667 | 4.0329 | 17.8921 | 19.8858 | 17.4246 | 20.6716 | 18.4872 | 17.7036 | 18.8544 | -0.3675 | low |
| TCGA-C5-A8ZZ | 0 | 636 | 21.2000 | 1.7425 | 18.5118 | 19.5716 | 20.2149 | 19.6459 | 14.5534 | 18.5962 | 20.8137 | -0.3825 | low |
| TCGA-MY-A5BD | 0 | 1667 | 55.5667 | 4.5671 | 19.9381 | 19.9759 | 19.7318 | 18.3706 | 14.5780 | 19.2552 | 18.6389 | -0.3885 | low |
| TCGA-BI-A20A | 0 | 720 | 24.0000 | 1.9726 | 16.9844 | 18.8620 | 20.1689 | 14.1624 | 16.6297 | 18.6547 | 17.4212 | -0.4040 | low |
| TCGA-JW-A5VI | 0 | 747 | 24.9000 | 2.0466 | 17.3644 | 20.1108 | 18.4702 | 19.3779 | 17.5487 | 16.8509 | 19.0706 | -0.4216 | low |
| TCGA-IR-A3LL | 0 | 1106 | 36.8667 | 3.0301 | 18.3187 | 19.6636 | 17.2194 | 21.4708 | 15.2859 | 18.1613 | 17.2867 | -0.4336 | low |
| TCGA-DS-A1OD | 0 | 3874 | 129.1333 | 10.6137 | 17.7281 | 19.2686 | 19.1680 | 17.4186 | 15.4595 | 18.1829 | 17.9956 | -0.4338 | low |
| TCGA-DS-A3LQ | 0 | 699 | 23.3000 | 1.9151 | 18.1605 | 19.2760 | 18.7291 | 23.2967 | 14.6514 | 19.5048 | 20.6173 | -0.4356 | low |
| TCGA-HM-A6W2 | 0 | 287 | 9.5667 | 0.7863 | 18.7786 | 18.0284 | 14.2693 | 19.7922 | 15.6103 | 18.0134 | 15.3892 | -0.4464 | low |
| TCGA-EX-A1H6 | 0 | 241 | 8.0333 | 0.6603 | 16.9988 | 19.1530 | 18.8173 | 16.0713 | 16.7662 | 17.4233 | 17.6110 | -0.4505 | low |
| TCGA-EK-A2R9 | 0 | 4 | 0.1333 | 0.0110 | 18.4863 | 20.1416 | 19.0607 | 19.3031 | 13.1823 | 17.7463 | 16.9222 | -0.4784 | low |
| TCGA-VS-A9UV | 1 | 104 | 3.4667 | 0.2849 | 18.9374 | 19.7279 | 18.8948 | 19.2004 | 17.0188 | 19.0234 | 20.0339 | -0.4933 | low |
| TCGA-VS-A9V2 | 0 | 555 | 18.5000 | 1.5205 | 17.3827 | 19.2078 | 18.2862 | 17.2299 | 17.0108 | 17.9454 | 17.7680 | -0.5044 | low |
| TCGA-C5-A2LX | 0 | 2526 | 84.2000 | 6.9205 | 17.6156 | 19.6649 | 18.8510 | 19.6029 | 15.7047 | 17.8955 | 19.1139 | -0.5109 | low |
| TCGA-DG-A2KJ | 0 | 2893 | 96.4333 | 7.9260 | 17.0128 | 20.3795 | 19.1520 | 17.1878 | 14.4870 | 16.5646 | 15.3857 | -0.5118 | low |
| TCGA-JW-A5VK | 0 | 623 | 20.7667 | 1.7068 | 18.9538 | 20.2679 | 18.2949 | 19.3933 | 19.2368 | 18.4433 | 20.3621 | -0.5188 | low |
| TCGA-VS-A9UZ | 0 | 2044 | 68.1333 | 5.6000 | 16.8502 | 18.7616 | 18.2051 | 18.0210 | 16.9888 | 18.6021 | 18.1282 | -0.5371 | low |
| TCGA-C5-A7X8 | 0 | 83 | 2.7667 | 0.2274 | 17.5490 | 19.4298 | 17.7012 | 18.8903 | 16.5789 | 17.7583 | 17.7738 | -0.5428 | low |
| TCGA-MA-AA42 | 0 | 259 | 8.6333 | 0.7096 | 18.2491 | 19.3586 | 18.4173 | 18.7871 | 15.0697 | 18.2479 | 18.3655 | -0.5494 | low |
| TCGA-MU-A5YI | 0 | 1053 | 35.1000 | 2.8849 | 17.2634 | 18.8043 | 18.7231 | 17.9208 | 14.8562 | 18.3165 | 18.1646 | -0.5588 | low |
| TCGA-HM-A3JK | 0 | 632 | 21.0667 | 1.7315 | 17.9073 | 20.1881 | 18.3773 | 21.1233 | 15.6271 | 18.1563 | 18.4428 | -0.5816 | low |
| TCGA-LP-A5U2 | 0 | 9 | 0.3000 | 0.0247 | 17.9763 | 20.2300 | 19.8605 | 18.5247 | 15.3440 | 18.6860 | 18.2655 | -0.6062 | low |
| TCGA-C5-A1BK | 0 | 5385 | 179.5000 | 14.7534 | 18.0102 | 19.6614 | 18.9011 | 19.0812 | 15.2034 | 18.0048 | 19.3595 | -0.6329 | low |
| TCGA-FU-A3NI | 1 | 638 | 21.2667 | 1.7479 | 17.5327 | 19.9428 | 20.3459 | 17.2812 | 14.4879 | 18.4737 | 18.0839 | -0.6510 | low |
| TCGA-C5-A1MI | 1 | 1083 | 36.1000 | 2.9671 | 17.8309 | 19.3480 | 19.7936 | 16.7599 | 15.0588 | 18.5488 | 19.5644 | -0.7666 | low |
| TCGA-VS-A9UU | 0 | 442 | 14.7333 | 1.2110 | 16.8321 | 18.8252 | 18.5353 | 17.1159 | 19.2503 | 18.3274 | 21.3944 | -0.7675 | low |
| TCGA-RA-A741 | 0 | 444 | 14.8000 | 1.2164 | 17.4220 | 19.0652 | 17.6995 | 17.1964 | 16.2034 | 17.6953 | 18.0655 | -0.7754 | low |
| TCGA-C5-A2M1 | 0 | 1169 | 38.9667 | 3.2027 | 17.2547 | 19.3241 | 18.3084 | 17.3392 | 17.4804 | 18.5093 | 18.6320 | -0.7908 | low |
| TCGA-IR-A3L7 | 0 | 4483 | 149.4333 | 12.2822 | 17.3478 | 19.5014 | 18.8182 | 16.0493 | 16.9808 | 17.6376 | 18.8781 | -0.7962 | low |
| TCGA-XS-A8TJ | 0 | 890 | 29.6667 | 2.4384 | 17.2540 | 19.1697 | 19.7786 | 16.1429 | 16.7586 | 18.6293 | 20.3778 | -0.8235 | low |
| TCGA-VS-A952 | 0 | 1778 | 59.2667 | 4.8712 | 12.8151 | 17.9228 | 19.1157 | 17.6437 | 17.2945 | 18.3500 | 18.6822 | -0.8325 | low |
| TCGA-C5-A7CO | 0 | 4482 | 149.4000 | 12.2795 | 17.5544 | 19.5507 | 18.9054 | 18.1339 | 15.3838 | 18.0894 | 19.3291 | -0.8363 | low |
| TCGA-C5-A1M8 | 0 | 919 | 30.6333 | 2.5178 | 18.7307 | 20.3284 | 17.7710 | 21.8098 | 15.3290 | 18.7792 | 18.8653 | -0.8395 | low |
| TCGA-MU-A51Y | 0 | 854 | 28.4667 | 2.3397 | 17.8416 | 20.1307 | 19.3974 | 18.9157 | 15.1255 | 18.1415 | 19.9197 | -0.9056 | low |
| TCGA-JW-AAVH | 0 | 552 | 18.4000 | 1.5123 | 17.9081 | 19.5593 | 20.0006 | 15.8474 | 17.0486 | 18.9304 | 20.8286 | -0.9341 | low |
| TCGA-ZJ-AAXF | 0 | 0 | 0.0000 | 0.0000 | 16.5463 | 19.2141 | 18.4543 | 18.1099 | 14.5744 | 17.0537 | 19.1931 | -0.9462 | low |
| TCGA-DG-A2KM | 0 | 1946 | 64.8667 | 5.3315 | 17.8561 | 18.9568 | 17.1865 | 17.8923 | 16.1128 | 17.6415 | 19.5948 | -0.9703 | low |
| TCGA-JW-A852 | 1 | 252 | 8.4000 | 0.6904 | 17.9350 | 20.7888 | 17.5786 | 18.7208 | 17.3867 | 16.8141 | 18.6101 | -1.0104 | low |
| TCGA-EA-A1QT | 0 | 1243 | 41.4333 | 3.4055 | 18.0393 | 20.0366 | 18.1113 | 16.7088 | 20.1951 | 18.1902 | 20.7671 | -1.0133 | low |
| TCGA-VS-A954 | 0 | 1714 | 57.1333 | 4.6959 | 17.7483 | 19.2902 | 17.2486 | 21.5267 | 14.0845 | 19.2019 | 18.3584 | -1.0142 | low |
| TCGA-EX-A1H5 | 0 | 619 | 20.6333 | 1.6959 | 18.5071 | 20.5613 | 20.0578 | 14.4491 | 18.8809 | 18.8080 | 19.9318 | -1.0203 | low |
| TCGA-C5-A905 | 0 | 4879 | 162.6333 | 13.3671 | 15.1722 | 20.1289 | 20.2661 | 20.2024 | 16.8201 | 18.6682 | 20.8950 | -1.0621 | low |
| TCGA-EK-A2RB | 0 | 9 | 0.3000 | 0.0247 | 16.5266 | 20.0195 | 17.0412 | 19.5042 | 17.0211 | 17.6519 | 17.4105 | -1.0866 | low |
| TCGA-ZJ-AAXA | 0 | 43 | 1.4333 | 0.1178 | 17.1917 | 19.4338 | 18.1862 | 21.1268 | 13.2377 | 18.7219 | 19.1192 | -1.0999 | low |
| TCGA-FU-A3EO | 0 | 490 | 16.3333 | 1.3425 | 14.2354 | 19.4348 | 17.2967 | 20.2778 | 17.0821 | 16.4929 | 19.3657 | -1.2011 | low |
| TCGA-C5-A1M5 | 1 | 2052 | 68.4000 | 5.6219 | 18.0925 | 20.2257 | 18.5938 | 17.7088 | 16.3601 | 18.9662 | 19.8913 | -1.3986 | low |
| TCGA-C5-A7CG | 0 | 6408 | 213.6000 | 17.5562 | 16.3169 | 19.8709 | 18.0760 | 18.4310 | 14.5604 | 17.4444 | 18.8261 | -1.4625 | low |
| TCGA-C5-A2LY | 0 | 2383 | 79.4333 | 6.5288 | 18.8246 | 20.7396 | 17.5046 | 18.1294 | 14.8854 | 18.3965 | 17.6848 | -1.5157 | low |
| TCGA-C5-A7UE | 0 | 4738 | 157.9333 | 12.9808 | 18.0351 | 20.0420 | 16.9855 | 18.6354 | 18.1294 | 18.1473 | 21.8262 | -1.6402 | low |
| TCGA-VS-A9UP | 0 | 1444 | 48.1333 | 3.9562 | 18.9677 | 20.0532 | 18.3164 | 14.5447 | 15.9278 | 19.7559 | 18.3303 | -1.7095 | low |
| TCGA-IR-A3LA | 0 | 4172 | 139.0667 | 11.4301 | 13.7341 | 19.4399 | 17.7373 | 16.3704 | 17.1363 | 19.2631 | 17.2240 | -2.1046 | low |
| TCGA-LP-A7HU | 0 | 406 | 13.5333 | 1.1123 | 17.8944 | 19.9887 | 17.3634 | 17.1174 | 12.9298 | 18.5004 | 18.9134 | -2.1612 | low |
| TCGA-MY-A5BE | 0 | 1066 | 35.5333 | 2.9205 | 16.9155 | 20.2572 | 17.8814 | 14.7294 | 14.1383 | 18.2024 | 17.7113 | -2.2636 | low |
| TCGA-EK-A2RK | 0 | 13 | 0.4333 | 0.0356 | 17.0210 | 20.6923 | 18.5978 | 15.3116 | 13.9699 | 18.4038 | 20.0991 | -2.6058 | low |

*OS Status: 0 means alive, 1 means dead.
