# Supplementary material for: Identification of Key Pathways and Establishment of a Seven-Gene Prognostic Signature in Cervical Cancer
Source: J Oncol. 2022 Feb 4;2022:4748796. doi: 10.1155/2022/4748796 (PMC8837458; doi:10.1155/2022/4748796)
Supplement: Supplementary Materials — Supplementary Figure 1: workflow chart of this study. Supplementary Figure 2: quality control of the six datasets. Supplementary Figure 3: KEGG analysis of the top 200 coexpressed genes of the 7 genes of the prognostic signature. KEGG, Kyoto Encyclopedia of Genes and Genomes. Supplementary Table 1: 108 Common DEGs of the six datasets. Supplementary Table 2: the associations between overall survival and 108 common DEGs using univariate cox analysis. Supplementary Table 3: the risk score and risk group of each patient of the TCGA database. Supplementary Table 4: gene sets enriched in the high-risk group. Supplementary Table 5: immune cells abundance analysis of the high-risk group and the low-risk group. [file 4748796.f1.zip › 4748796.f1/Supplementary Table 4.docx]

Supplementary Table 4. Gene sets enriched in the high-risk group.

| **Gene set** | **NES** | **NOM p-val** | **FDR q-val** |
| --- | --- | --- | --- |
| KEGG_ADHERENS_JUNCTION | 1.97 | 0.008 | 0.131 |
| KEGG_FOCAL_ADHESION | 1.8 | 0.013 | 0.193 |
| KEGG_ECM_RECEPTOR_INTERACTION | 1.75 | 0.011 | 0.216 |
| KEGG_RENAL_CELL_CARCINOMA | 1.69 | 0.041 | 0.237 |
| KEGG_SMALL_CELL_LUNG_CANCER | 1.68 | 0.021 | 0.22 |
