# Supplementary material for: Identification of Key Pathways and Establishment of a Seven-Gene Prognostic Signature in Cervical Cancer
Source: J Oncol. 2022 Feb 4;2022:4748796. doi: 10.1155/2022/4748796 (PMC8837458; doi:10.1155/2022/4748796)
Supplement: Supplementary Materials — Supplementary Figure 1: workflow chart of this study. Supplementary Figure 2: quality control of the six datasets. Supplementary Figure 3: KEGG analysis of the top 200 coexpressed genes of the 7 genes of the prognostic signature. KEGG, Kyoto Encyclopedia of Genes and Genomes. Supplementary Table 1: 108 Common DEGs of the six datasets. Supplementary Table 2: the associations between overall survival and 108 common DEGs using univariate cox analysis. Supplementary Table 3: the risk score and risk group of each patient of the TCGA database. Supplementary Table 4: gene sets enriched in the high-risk group. Supplementary Table 5: immune cells abundance analysis of the high-risk group and the low-risk group. [file 4748796.f1.zip › 4748796.f1/Supplementary Table 5.docx]

Supplementary Table 5. Immune cells abundance analysis of the high-risk group and the low-risk group.

| **submitter_id** | **group** | **CD4_naive** | **CD8_naive** | **Cytotoxic** | **Exhausted** | **Tr1** | **nTreg** | **iTreg** | **Th1** | **Th2** | **Th17** | **Tfh** | **Central_memory** | **Effector_memory** | **NKT** | **MAIT** | **DC** | **Bcell** | **Monocyte** | **Macrophage** | **NK** | **Neutrophil** | **Gamma_delta** | **CD4_T** | **CD8_T** |
| --- | --- | --- | --- | --- | --- | --- | --- | --- | --- | --- | --- | --- | --- | --- | --- | --- | --- | --- | --- | --- | --- | --- | --- | --- | --- |
| TCGA-DS-A7WH | high | 0 | 0.095 | 0 | 0 | 0.001 | 0.069 | 0.127 | 0.078 | 0.091 | 0.142 | 0.167 | 0.068 | 0 | 0.076 | 0.079 | 0.065 | 0.081 | 0.057 | 0.107 | 0.176 | 0.122 | 0.056 | 0.113 | 0.026 |
| TCGA-ZJ-A8QR | high | 0 | 0.07 | 0.07 | 0 | 0 | 0.074 | 0.144 | 0.101 | 0.126 | 0.079 | 0.116 | 0.073 | 0 | 0.083 | 0.016 | 0.232 | 0.087 | 0.092 | 0.13 | 0.147 | 0.148 | 0.036 | 0.025 | 0.084 |
| TCGA-LP-A4AX | high | 0 | 0.057 | 0.057 | 0 | 0.051 | 0.056 | 0.137 | 0.092 | 0.107 | 0.088 | 0.115 | 0.068 | 0 | 0.039 | 0.01 | 0.188 | 0.147 | 0.045 | 0.168 | 0.159 | 0.092 | 0.04 | 0.107 | 0.106 |
| TCGA-FU-A3HZ | high | 0 | 0.062 | 0.119 | 0 | 0 | 0.048 | 0.107 | 0.104 | 0.123 | 0.1 | 0.075 | 0.04 | 0 | 0.032 | 0.092 | 0.126 | 0.158 | 0.007 | 0.138 | 0.132 | 0.12 | 0.038 | 0.07 | 0.138 |
| TCGA-VS-A9UT | high | 0 | 0.064 | 0.069 | 0.003 | 0.071 | 0.048 | 0.13 | 0.082 | 0.108 | 0 | 0.124 | 0.029 | 0 | 0.023 | 0.096 | 0.209 | 0.182 | 0.035 | 0.19 | 0.112 | 0.101 | 0.036 | 0.114 | 0.096 |
| TCGA-EK-A2IP | high | 0 | 0.072 | 0.051 | 0 | 0 | 0.046 | 0.118 | 0.097 | 0.122 | 0.133 | 0.108 | 0.074 | 0 | 0.06 | 0.067 | 0.131 | 0.102 | 0.037 | 0.077 | 0.169 | 0.123 | 0.061 | 0.088 | 0.111 |
| TCGA-EA-A50E | high | 0 | 0.064 | 0.056 | 0 | 0.01 | 0.045 | 0.123 | 0.086 | 0.118 | 0.062 | 0.116 | 0.077 | 0 | 0.05 | 0.081 | 0.227 | 0.142 | 0.061 | 0.192 | 0.174 | 0.106 | 0.042 | 0.04 | 0.091 |
| TCGA-ZJ-AAXT | high | 0 | 0.067 | 0.022 | 0 | 0.012 | 0.071 | 0.124 | 0.09 | 0.118 | 0.07 | 0.159 | 0.088 | 0 | 0.043 | 0.077 | 0.137 | 0.097 | 0.066 | 0.143 | 0.147 | 0.139 | 0.037 | 0.086 | 0.097 |
| TCGA-VS-A9V4 | high | 0 | 0.093 | 0.058 | 0 | 0.016 | 0 | 0.046 | 0.098 | 0.116 | 0.119 | 0.06 | 0.102 | 0 | 0.122 | 0.099 | 0.043 | 0.069 | 0.05 | 0.063 | 0.166 | 0.165 | 0.055 | 0.091 | 0.005 |
| TCGA-EA-A5O9 | high | 0 | 0.067 | 0.026 | 0 | 0 | 0.047 | 0.126 | 0.116 | 0.112 | 0.13 | 0.101 | 0.079 | 0 | 0.024 | 0.085 | 0.257 | 0.063 | 0.101 | 0.179 | 0.119 | 0.103 | 0.051 | 0 | 0.137 |
| TCGA-FU-A57G | high | 0 | 0.091 | 0.032 | 0 | 0 | 0.041 | 0.047 | 0.076 | 0.125 | 0.09 | 0.155 | 0.074 | 0 | 0.069 | 0.106 | 0.188 | 0.091 | 0.045 | 0.136 | 0.12 | 0.139 | 0.066 | 0.094 | 0.035 |
| TCGA-EX-A8YF | high | 0 | 0.049 | 0.048 | 0 | 0.056 | 0.05 | 0.132 | 0.073 | 0.109 | 0.108 | 0.117 | 0.067 | 0 | 0 | 0.073 | 0.109 | 0.205 | 0.031 | 0.136 | 0.138 | 0.136 | 0.06 | 0.079 | 0.11 |
| TCGA-MA-AA43 | high | 0 | 0.074 | 0.009 | 0 | 0.016 | 0.046 | 0.131 | 0.107 | 0.118 | 0.105 | 0.079 | 0.061 | 0 | 0.074 | 0.106 | 0.158 | 0.052 | 0.057 | 0.13 | 0.153 | 0.152 | 0.034 | 0.087 | 0.099 |
| TCGA-JW-A5VG | high | 0 | 0.058 | 0.026 | 0 | 0 | 0.051 | 0.136 | 0.107 | 0.129 | 0.079 | 0.139 | 0.071 | 0 | 0.028 | 0.09 | 0.219 | 0.084 | 0.081 | 0.178 | 0.128 | 0.081 | 0.055 | 0.091 | 0.085 |
| TCGA-2W-A8YY | high | 0 | 0.074 | 0.037 | 0 | 0.011 | 0.049 | 0.121 | 0.086 | 0.113 | 0.151 | 0.071 | 0.04 | 0 | 0.048 | 0.081 | 0.175 | 0.144 | 0.027 | 0.16 | 0.127 | 0.133 | 0.04 | 0.075 | 0.089 |
| TCGA-LP-A4AW | high | 0 | 0.069 | 0.049 | 0 | 0.019 | 0.046 | 0.064 | 0.076 | 0.123 | 0.03 | 0.157 | 0.061 | 0 | 0.072 | 0.094 | 0.204 | 0.186 | 0.04 | 0.11 | 0.139 | 0.119 | 0.058 | 0.112 | 0.077 |
| TCGA-EA-A3HQ | high | 0 | 0.063 | 0.035 | 0 | 0.041 | 0.06 | 0.127 | 0.101 | 0.12 | 0.052 | 0.089 | 0.088 | 0 | 0.048 | 0.062 | 0.29 | 0.11 | 0.087 | 0.206 | 0.093 | 0.081 | 0.031 | 0.076 | 0.095 |
| TCGA-BI-A0VR | high | 0 | 0.059 | 0.065 | 0 | 0.046 | 0.057 | 0.141 | 0.083 | 0.114 | 0.112 | 0.168 | 0.073 | 0 | 0.011 | 0.001 | 0.23 | 0.173 | 0.043 | 0.14 | 0.153 | 0.092 | 0.03 | 0.087 | 0.118 |
| TCGA-VS-A8QH | high | 0 | 0 | 0.04 | 0 | 0 | 0.063 | 0.027 | 0.1 | 0.119 | 0.14 | 0.078 | 0.098 | 0.01 | 0.122 | 0.097 | 0.187 | 0.104 | 0.069 | 0.079 | 0.119 | 0.145 | 0.051 | 0.061 | 0.097 |
| TCGA-HM-A3JJ | high | 0 | 0.048 | 0.066 | 0 | 0.024 | 0.059 | 0.111 | 0.098 | 0.13 | 0.036 | 0.109 | 0.062 | 0 | 0.055 | 0.083 | 0.233 | 0.161 | 0.056 | 0.145 | 0.134 | 0.086 | 0.044 | 0.098 | 0.092 |
| TCGA-C5-A1M6 | high | 0 | 0.122 | 0.01 | 0.001 | 0 | 0 | 0.066 | 0.105 | 0.116 | 0.148 | 0.066 | 0.08 | 0 | 0.1 | 0.109 | 0.069 | 0.096 | 0.082 | 0.068 | 0.122 | 0.11 | 0.068 | 0.095 | 0.013 |
| TCGA-C5-A7CH | high | 0 | 0.093 | 0.027 | 0 | 0.003 | 0.05 | 0.134 | 0.098 | 0.135 | 0.122 | 0.104 | 0.084 | 0 | 0.035 | 0 | 0.231 | 0.096 | 0.059 | 0.126 | 0.168 | 0.154 | 0.037 | 0.078 | 0.089 |
| TCGA-DG-A2KL | high | 0 | 0.075 | 0.057 | 0 | 0.009 | 0.069 | 0.135 | 0.096 | 0.125 | 0.092 | 0.079 | 0.085 | 0 | 0.043 | 0.011 | 0.32 | 0.089 | 0.083 | 0.159 | 0.103 | 0.117 | 0.026 | 0.096 | 0.103 |
| TCGA-EX-A69L | high | 0 | 0.067 | 0.057 | 0 | 0.03 | 0.057 | 0.117 | 0.094 | 0.125 | 0.113 | 0.098 | 0.05 | 0 | 0 | 0.083 | 0.185 | 0.209 | 0.033 | 0.182 | 0.147 | 0.092 | 0.018 | 0.095 | 0.094 |
| TCGA-VS-A9U6 | high | 0 | 0.066 | 0.068 | 0 | 0.032 | 0.055 | 0.119 | 0.087 | 0.119 | 0.096 | 0.129 | 0.048 | 0 | 0.044 | 0.032 | 0.208 | 0.168 | 0.068 | 0.125 | 0.179 | 0.107 | 0.035 | 0.104 | 0.083 |
| TCGA-C5-A1MP | high | 0 | 0.07 | 0.05 | 0 | 0.061 | 0.051 | 0.127 | 0.062 | 0.109 | 0.065 | 0.134 | 0.058 | 0 | 0.02 | 0.089 | 0.256 | 0.115 | 0.044 | 0.168 | 0.153 | 0.061 | 0.019 | 0.102 | 0.12 |
| TCGA-VS-A9UJ | high | 0 | 0.1 | 0.065 | 0 | 0 | 0 | 0 | 0.116 | 0.123 | 0.175 | 0 | 0 | 0 | 0.118 | 0.008 | 0.12 | 0.046 | 0.142 | 0.069 | 0 | 0.174 | 0.073 | 0.067 | 0.099 |
| TCGA-C5-A3HD | high | 0 | 0.09 | 0.021 | 0 | 0 | 0.074 | 0.138 | 0.129 | 0.118 | 0.116 | 0.118 | 0.039 | 0 | 0.084 | 0.065 | 0.185 | 0.07 | 0.083 | 0.068 | 0.087 | 0.109 | 0.055 | 0.084 | 0.095 |
| TCGA-C5-A907 | high | 0 | 0.039 | 0.043 | 0 | 0 | 0.06 | 0.129 | 0.11 | 0.118 | 0.17 | 0.007 | 0.085 | 0 | 0.067 | 0.067 | 0.143 | 0.141 | 0.06 | 0.129 | 0.168 | 0.12 | 0 | 0.09 | 0.099 |
| TCGA-IR-A3LI | high | 0.017 | 0.03 | 0 | 0.001 | 0 | 0.082 | 0 | 0 | 0 | 0.165 | 0.127 | 0.102 | 0.01 | 0.046 | 0 | 0.14 | 0.109 | 0.108 | 0.063 | 0.026 | 0.102 | 0.081 | 0.145 | 0.051 |
| TCGA-C5-A1MQ | high | 0 | 0.039 | 0.046 | 0 | 0.03 | 0.037 | 0.122 | 0.075 | 0.121 | 0.122 | 0.091 | 0.045 | 0 | 0.046 | 0.086 | 0.259 | 0.182 | 0.06 | 0.224 | 0.17 | 0.025 | 0.056 | 0.077 | 0.071 |
| TCGA-EK-A2PL | high | 0 | 0.066 | 0.051 | 0 | 0 | 0.055 | 0.133 | 0.114 | 0.116 | 0.119 | 0.108 | 0.091 | 0 | 0.056 | 0.036 | 0.209 | 0.074 | 0.068 | 0.102 | 0.133 | 0.081 | 0.047 | 0.067 | 0.137 |
| TCGA-MY-A913 | high | 0 | 0.043 | 0.058 | 0 | 0.045 | 0.058 | 0.134 | 0.12 | 0.135 | 0.065 | 0.087 | 0.073 | 0 | 0.002 | 0.063 | 0.199 | 0.153 | 0.025 | 0.145 | 0.132 | 0.154 | 0.03 | 0.085 | 0.092 |
| TCGA-C5-A2LT | high | 0 | 0.051 | 0.073 | 0 | 0.027 | 0.052 | 0.125 | 0.089 | 0.135 | 0.045 | 0.146 | 0.068 | 0 | 0.048 | 0.094 | 0.173 | 0.123 | 0.048 | 0.162 | 0.169 | 0.1 | 0.028 | 0.065 | 0.105 |
| TCGA-C5-A1ML | high | 0 | 0.069 | 0.067 | 0 | 0.002 | 0.061 | 0.137 | 0.094 | 0.116 | 0.054 | 0.116 | 0.057 | 0 | 0 | 0.104 | 0.229 | 0.085 | 0.074 | 0.141 | 0.126 | 0.096 | 0.06 | 0.112 | 0.078 |
| TCGA-JX-A3PZ | high | 0 | 0.066 | 0.06 | 0 | 0 | 0.067 | 0.119 | 0.105 | 0.138 | 0.114 | 0.023 | 0.079 | 0 | 0.045 | 0.055 | 0.283 | 0.06 | 0.084 | 0.205 | 0.154 | 0.056 | 0.015 | 0.074 | 0.087 |
| TCGA-EK-A2PI | high | 0 | 0.095 | 0.004 | 0 | 0 | 0.054 | 0.131 | 0.089 | 0.123 | 0.14 | 0.12 | 0.069 | 0 | 0.048 | 0.059 | 0.22 | 0.088 | 0.086 | 0.097 | 0.14 | 0.1 | 0.039 | 0.072 | 0.107 |
| TCGA-EK-A2GZ | high | 0 | 0.07 | 0.074 | 0 | 0 | 0.045 | 0.126 | 0.097 | 0.128 | 0.107 | 0.115 | 0.069 | 0 | 0.038 | 0.045 | 0.195 | 0.159 | 0.065 | 0.139 | 0.131 | 0.075 | 0.045 | 0.08 | 0.099 |
| TCGA-C5-A901 | high | 0 | 0.077 | 0.048 | 0 | 0.017 | 0.044 | 0.124 | 0.066 | 0.116 | 0.131 | 0.158 | 0.067 | 0 | 0.031 | 0.057 | 0.191 | 0.15 | 0.042 | 0.132 | 0.176 | 0.133 | 0.023 | 0.083 | 0.089 |
| TCGA-C5-A8XK | high | 0 | 0.037 | 0.071 | 0 | 0 | 0.047 | 0.121 | 0.1 | 0.106 | 0.078 | 0.13 | 0.051 | 0 | 0.073 | 0.092 | 0.153 | 0.127 | 0.078 | 0.164 | 0.122 | 0.12 | 0.058 | 0.082 | 0.074 |
| TCGA-FU-A3HY | high | 0 | 0.099 | 0.026 | 0 | 0 | 0.069 | 0.117 | 0.111 | 0.146 | 0.125 | 0.001 | 0.123 | 0 | 0.017 | 0.061 | 0.184 | 0.072 | 0.077 | 0.077 | 0.177 | 0.173 | 0.005 | 0.075 | 0.091 |
| TCGA-VS-A8EH | high | 0 | 0.067 | 0 | 0 | 0 | 0.051 | 0.135 | 0.107 | 0.118 | 0.122 | 0.111 | 0.085 | 0.006 | 0.036 | 0.084 | 0.227 | 0.073 | 0.076 | 0.137 | 0.095 | 0.1 | 0.05 | 0.089 | 0.104 |
| TCGA-C5-A1MK | high | 0 | 0.077 | 0.038 | 0 | 0 | 0.055 | 0.126 | 0.095 | 0.086 | 0.123 | 0.155 | 0.088 | 0 | 0 | 0.08 | 0.185 | 0.039 | 0.057 | 0.177 | 0.12 | 0.079 | 0.041 | 0.098 | 0.097 |
| TCGA-C5-A1M9 | high | 0 | 0.087 | 0.026 | 0 | 0 | 0.074 | 0.115 | 0.128 | 0.124 | 0.12 | 0.072 | 0.094 | 0 | 0.074 | 0 | 0.144 | 0.062 | 0.114 | 0.177 | 0.106 | 0.151 | 0.062 | 0 | 0.07 |
| TCGA-C5-A3HE | high | 0 | 0.031 | 0.081 | 0 | 0.014 | 0.052 | 0.13 | 0.094 | 0.118 | 0.094 | 0.115 | 0.049 | 0 | 0.013 | 0.088 | 0.159 | 0.166 | 0.06 | 0.129 | 0.171 | 0.149 | 0.025 | 0.089 | 0.106 |
| TCGA-EX-A69M | high | 0 | 0.046 | 0.025 | 0 | 0.035 | 0.066 | 0.139 | 0.101 | 0.108 | 0.095 | 0.126 | 0.049 | 0 | 0.012 | 0.076 | 0.217 | 0.129 | 0.074 | 0.153 | 0.118 | 0.131 | 0.04 | 0.1 | 0.098 |
| TCGA-EA-A3QD | high | 0 | 0.064 | 0.073 | 0 | 0.064 | 0.055 | 0.136 | 0.081 | 0.125 | 0.062 | 0.063 | 0.045 | 0 | 0.004 | 0.093 | 0.247 | 0.233 | 0.018 | 0.176 | 0.132 | 0.049 | 0.023 | 0.109 | 0.107 |
| TCGA-C5-A7CL | high | 0 | 0.048 | 0.062 | 0 | 0 | 0.053 | 0.128 | 0.087 | 0.105 | 0.138 | 0.118 | 0.061 | 0 | 0.065 | 0.067 | 0.158 | 0.115 | 0.06 | 0.139 | 0.133 | 0.153 | 0.02 | 0.099 | 0.07 |
| TCGA-VS-A8EB | high | 0 | 0.069 | 0.082 | 0 | 0 | 0.049 | 0.126 | 0.123 | 0.111 | 0.139 | 0.063 | 0.072 | 0 | 0.029 | 0.095 | 0.162 | 0.066 | 0.066 | 0.14 | 0.157 | 0.038 | 0.073 | 0.067 | 0.132 |
| TCGA-EK-A2H1 | high | 0 | 0.063 | 0.066 | 0 | 0 | 0.052 | 0.13 | 0.112 | 0.145 | 0.03 | 0.114 | 0.063 | 0 | 0.033 | 0.092 | 0.242 | 0.129 | 0.072 | 0.19 | 0.181 | 0.11 | 0.027 | 0.085 | 0.042 |
| TCGA-VS-A8EC | high | 0 | 0.134 | 0.044 | 0 | 0 | 0.063 | 0.141 | 0.106 | 0.129 | 0.036 | 0.095 | 0.013 | 0 | 0.068 | 0.067 | 0.155 | 0.174 | 0.033 | 0.071 | 0.136 | 0.134 | 0.051 | 0.078 | 0.091 |
| TCGA-EA-A3HR | high | 0 | 0.077 | 0.069 | 0 | 0.001 | 0.053 | 0.121 | 0.071 | 0.123 | 0.082 | 0.087 | 0.067 | 0 | 0.023 | 0.089 | 0.202 | 0.126 | 0.024 | 0.109 | 0.155 | 0.129 | 0.057 | 0.086 | 0.109 |
| TCGA-EK-A2RE | high | 0 | 0.1 | 0.031 | 0 | 0 | 0.065 | 0.075 | 0.11 | 0 | 0.109 | 0.156 | 0.084 | 0 | 0.094 | 0.002 | 0.232 | 0.034 | 0.105 | 0.111 | 0.158 | 0.159 | 0.029 | 0.057 | 0.009 |
| TCGA-EK-A3GN | high | 0.006 | 0.091 | 0.057 | 0 | 0 | 0 | 0.145 | 0 | 0.136 | 0.073 | 0.098 | 0.085 | 0 | 0.062 | 0 | 0.193 | 0.133 | 0.086 | 0.119 | 0.211 | 0.115 | 0.067 | 0.105 | 0 |
| TCGA-C5-A7UC | high | 0 | 0.106 | 0.033 | 0 | 0 | 0.072 | 0.133 | 0.109 | 0.133 | 0.165 | 0.147 | 0.054 | 0 | 0.028 | 0 | 0.112 | 0 | 0.097 | 0.087 | 0.146 | 0.106 | 0.075 | 0.089 | 0.102 |
| TCGA-DS-A1OB | high | 0 | 0.066 | 0.047 | 0 | 0 | 0.05 | 0.145 | 0.095 | 0.112 | 0.04 | 0.128 | 0.051 | 0 | 0.042 | 0.089 | 0.202 | 0.134 | 0.055 | 0.193 | 0.062 | 0.102 | 0.04 | 0.091 | 0.103 |
| TCGA-C5-A0TN | high | 0 | 0.072 | 0.081 | 0 | 0 | 0.066 | 0.14 | 0.131 | 0.143 | 0.065 | 0.125 | 0.094 | 0 | 0 | 0 | 0.188 | 0.038 | 0.092 | 0.177 | 0.117 | 0.123 | 0.058 | 0.083 | 0.075 |
| TCGA-VS-A8QC | high | 0 | 0.081 | 0.044 | 0 | 0.002 | 0.046 | 0.127 | 0.076 | 0.117 | 0.124 | 0.108 | 0.093 | 0 | 0 | 0.077 | 0.225 | 0.116 | 0.06 | 0.134 | 0.148 | 0.073 | 0.049 | 0.089 | 0.126 |
| TCGA-EK-A3GK | high | 0 | 0.081 | 0.029 | 0 | 0 | 0.058 | 0.141 | 0.108 | 0.131 | 0.092 | 0.119 | 0.091 | 0 | 0.008 | 0.083 | 0.122 | 0.13 | 0.059 | 0.114 | 0.143 | 0.088 | 0.052 | 0.074 | 0.104 |
| TCGA-DS-A1OC | high | 0 | 0.047 | 0.052 | 0 | 0.024 | 0.059 | 0.144 | 0.079 | 0.126 | 0.098 | 0.157 | 0.06 | 0 | 0.003 | 0.065 | 0.232 | 0.156 | 0.052 | 0.161 | 0.179 | 0.071 | 0.039 | 0.076 | 0.106 |
| TCGA-VS-A8QA | high | 0 | 0.09 | 0.09 | 0 | 0 | 0.053 | 0.113 | 0.125 | 0.051 | 0.122 | 0.103 | 0.065 | 0 | 0.063 | 0.068 | 0.09 | 0.102 | 0.01 | 0.085 | 0.141 | 0.109 | 0.047 | 0.075 | 0.131 |
| TCGA-Q1-A73O | high | 0 | 0.068 | 0.043 | 0 | 0.02 | 0.057 | 0.136 | 0.106 | 0.118 | 0.128 | 0.076 | 0.074 | 0 | 0.005 | 0.082 | 0.181 | 0.128 | 0.07 | 0.135 | 0.176 | 0.091 | 0.046 | 0.068 | 0.133 |
| TCGA-C5-A2LV | high | 0 | 0.034 | 0.07 | 0 | 0.004 | 0.069 | 0.136 | 0.135 | 0.139 | 0.03 | 0.12 | 0.09 | 0 | 0.074 | 0.002 | 0.275 | 0.13 | 0.093 | 0.185 | 0.113 | 0.081 | 0.043 | 0.05 | 0.101 |
| TCGA-VS-A94Y | high | 0 | 0.074 | 0.06 | 0 | 0 | 0.06 | 0.132 | 0.101 | 0.118 | 0.146 | 0 | 0.075 | 0 | 0.076 | 0.055 | 0.203 | 0.038 | 0.102 | 0.138 | 0.157 | 0.118 | 0 | 0.09 | 0.102 |
| TCGA-EA-A4BA | high | 0 | 0.074 | 0.06 | 0 | 0 | 0.061 | 0.136 | 0 | 0.134 | 0.097 | 0 | 0 | 0 | 0.077 | 0.105 | 0.156 | 0.132 | 0.063 | 0.096 | 0.216 | 0.083 | 0.036 | 0.074 | 0.062 |
| TCGA-DR-A0ZM | high | 0 | 0.068 | 0.078 | 0.001 | 0 | 0.061 | 0.143 | 0.094 | 0.121 | 0.024 | 0.092 | 0.068 | 0 | 0.024 | 0.092 | 0.216 | 0.148 | 0.038 | 0.152 | 0.116 | 0.028 | 0.038 | 0.116 | 0.14 |
| TCGA-EK-A2R8 | high | 0 | 0.001 | 0.06 | 0 | 0 | 0.074 | 0.042 | 0.131 | 0.115 | 0.136 | 0.099 | 0.106 | 0.005 | 0.049 | 0.082 | 0.079 | 0.013 | 0.11 | 0.102 | 0.175 | 0.115 | 0.077 | 0.043 | 0.127 |
| TCGA-C5-A7CM | high | 0 | 0.055 | 0.025 | 0 | 0 | 0.041 | 0.139 | 0.081 | 0.112 | 0.148 | 0.134 | 0.092 | 0 | 0.011 | 0.082 | 0.082 | 0.17 | 0.067 | 0.056 | 0.18 | 0.144 | 0.038 | 0.111 | 0.076 |
| TCGA-C5-A1MN | high | 0 | 0.093 | 0.027 | 0 | 0 | 0.071 | 0.142 | 0.116 | 0.14 | 0.041 | 0.092 | 0.086 | 0 | 0.065 | 0.054 | 0.237 | 0.093 | 0.058 | 0.125 | 0.173 | 0.145 | 0 | 0.072 | 0.07 |
| TCGA-EA-A439 | high | 0 | 0.088 | 0.057 | 0 | 0 | 0.057 | 0.133 | 0.115 | 0.103 | 0.06 | 0.083 | 0.073 | 0 | 0.058 | 0.08 | 0.155 | 0.126 | 0.044 | 0.138 | 0.102 | 0.088 | 0.053 | 0.091 | 0.094 |
| TCGA-VS-A9V1 | high | 0 | 0.055 | 0.013 | 0 | 0.009 | 0 | 0.132 | 0.087 | 0.115 | 0.147 | 0.145 | 0.067 | 0 | 0.001 | 0.085 | 0 | 0.184 | 0.094 | 0.061 | 0.171 | 0.219 | 0.065 | 0.118 | 0 |
| TCGA-EX-A3L1 | high | 0 | 0.023 | 0.059 | 0 | 0.021 | 0.047 | 0.119 | 0.069 | 0.125 | 0.138 | 0.134 | 0.066 | 0 | 0.065 | 0.062 | 0.157 | 0.117 | 0.06 | 0.133 | 0.145 | 0.181 | 0.039 | 0.069 | 0.061 |
| TCGA-C5-A1BN | high | 0 | 0.043 | 0.046 | 0 | 0 | 0.064 | 0.131 | 0.098 | 0.111 | 0.176 | 0.111 | 0.101 | 0.009 | 0.083 | 0.073 | 0.181 | 0.089 | 0.061 | 0.012 | 0.13 | 0.128 | 0.037 | 0.042 | 0.1 |
| TCGA-UC-A7PD | high | 0 | 0.079 | 0.069 | 0 | 0 | 0.05 | 0.144 | 0.102 | 0.123 | 0.112 | 0.086 | 0.081 | 0 | 0.079 | 0.005 | 0.191 | 0.129 | 0.058 | 0.102 | 0.139 | 0.118 | 0.041 | 0.097 | 0.093 |
| TCGA-EK-A2PK | high | 0 | 0.047 | 0.063 | 0.005 | 0.028 | 0.059 | 0.138 | 0.081 | 0.121 | 0.104 | 0.111 | 0.065 | 0 | 0 | 0.078 | 0.17 | 0.173 | 0.036 | 0.164 | 0.16 | 0.105 | 0.064 | 0.074 | 0.086 |
| TCGA-ZJ-AB0I | high | 0 | 0.08 | 0.067 | 0 | 0 | 0.053 | 0.131 | 0.098 | 0.119 | 0.077 | 0.18 | 0.08 | 0 | 0.06 | 0.05 | 0.251 | 0.009 | 0.038 | 0.161 | 0.2 | 0.028 | 0.045 | 0.04 | 0.125 |
| TCGA-JW-A69B | high | 0 | 0.037 | 0.092 | 0 | 0.042 | 0.069 | 0.116 | 0.108 | 0.116 | 0.107 | 0 | 0.075 | 0 | 0.06 | 0.065 | 0.225 | 0.148 | 0.038 | 0.191 | 0.094 | 0.117 | 0 | 0.091 | 0.069 |
| TCGA-VS-A9UL | high | 0 | 0.035 | 0.054 | 0 | 0 | 0.057 | 0 | 0.108 | 0.115 | 0.124 | 0.117 | 0.061 | 0.024 | 0.096 | 0.11 | 0.065 | 0.105 | 0.038 | 0.076 | 0.106 | 0.154 | 0.052 | 0.107 | 0.094 |
| TCGA-DS-A7WI | high | 0 | 0.05 | 0.03 | 0 | 0 | 0.033 | 0.126 | 0.1 | 0.136 | 0.119 | 0.114 | 0.06 | 0 | 0.075 | 0.019 | 0.2 | 0.013 | 0.086 | 0.182 | 0.176 | 0.085 | 0.066 | 0.069 | 0.119 |
| TCGA-ZJ-AB0H | high | 0 | 0.049 | 0.04 | 0 | 0.074 | 0.06 | 0.132 | 0.085 | 0.089 | 0.067 | 0.145 | 0.062 | 0 | 0.022 | 0.022 | 0.25 | 0.191 | 0.041 | 0.168 | 0.13 | 0.118 | 0.026 | 0.105 | 0.097 |
| TCGA-EA-A3HT | high | 0 | 0.058 | 0.046 | 0 | 0.018 | 0.052 | 0.132 | 0.082 | 0.108 | 0.119 | 0.146 | 0.059 | 0 | 0.051 | 0.081 | 0.222 | 0.085 | 0.089 | 0.167 | 0.13 | 0.093 | 0.027 | 0.076 | 0.092 |
| TCGA-EA-A3QE | high | 0 | 0.062 | 0.061 | 0 | 0.05 | 0.059 | 0.137 | 0.072 | 0.121 | 0.079 | 0.135 | 0.052 | 0 | 0.015 | 0.061 | 0.218 | 0.189 | 0.014 | 0.124 | 0.157 | 0.097 | 0.036 | 0.094 | 0.111 |
| TCGA-EA-A3Y4 | high | 0 | 0 | 0.048 | 0.023 | 0 | 0.059 | 0.05 | 0.109 | 0.131 | 0.079 | 0.14 | 0.065 | 0 | 0.043 | 0.076 | 0.179 | 0.143 | 0.043 | 0.155 | 0.192 | 0.061 | 0.051 | 0.117 | 0.136 |
| TCGA-FU-A770 | high | 0 | 0.064 | 0.075 | 0 | 0 | 0.036 | 0.133 | 0.078 | 0.126 | 0.116 | 0.144 | 0.073 | 0 | 0.019 | 0.085 | 0.13 | 0.105 | 0.042 | 0.083 | 0.145 | 0.194 | 0.037 | 0.086 | 0.087 |
| TCGA-EK-A2RJ | high | 0 | 0.026 | 0.051 | 0 | 0.012 | 0.058 | 0.125 | 0.109 | 0.123 | 0.155 | 0.135 | 0.063 | 0 | 0.02 | 0.078 | 0.138 | 0.149 | 0.04 | 0.185 | 0.167 | 0.095 | 0.026 | 0.076 | 0.112 |
| TCGA-C5-A1BM | high | 0 | 0.03 | 0.056 | 0 | 0.039 | 0.06 | 0.129 | 0.072 | 0.116 | 0.045 | 0.172 | 0.057 | 0 | 0.025 | 0.069 | 0.267 | 0.161 | 0.06 | 0.197 | 0.121 | 0.118 | 0.019 | 0.07 | 0.101 |
| TCGA-BI-A0VS | high | 0 | 0.079 | 0.058 | 0 | 0.054 | 0.053 | 0.138 | 0.084 | 0.123 | 0.017 | 0.1 | 0.076 | 0 | 0.028 | 0.067 | 0.214 | 0.204 | 0.015 | 0.115 | 0.178 | 0.071 | 0.046 | 0.1 | 0.098 |
| TCGA-VS-A950 | high | 0 | 0.042 | 0.01 | 0 | 0.05 | 0.049 | 0.141 | 0.093 | 0.114 | 0.145 | 0.084 | 0.094 | 0 | 0.083 | 0 | 0.22 | 0.143 | 0.05 | 0.158 | 0.145 | 0.107 | 0.032 | 0.042 | 0.093 |
| TCGA-C5-A8XH | high | 0 | 0.068 | 0.053 | 0 | 0 | 0.069 | 0.128 | 0.123 | 0.122 | 0.102 | 0.101 | 0.064 | 0 | 0.019 | 0.075 | 0.258 | 0.099 | 0.061 | 0.156 | 0.135 | 0.087 | 0.035 | 0.092 | 0.087 |
| TCGA-DS-A5RQ | high | 0 | 0.053 | 0.041 | 0 | 0.015 | 0.054 | 0.121 | 0.108 | 0.132 | 0.084 | 0.144 | 0.071 | 0 | 0.041 | 0.007 | 0.228 | 0.157 | 0.067 | 0.17 | 0.16 | 0.098 | 0.034 | 0.071 | 0.116 |
| TCGA-C5-A8YQ | high | 0 | 0.072 | 0.038 | 0 | 0 | 0.043 | 0.126 | 0.096 | 0.116 | 0.08 | 0.117 | 0.062 | 0 | 0.043 | 0.076 | 0.153 | 0.147 | 0.052 | 0.163 | 0.057 | 0.072 | 0.066 | 0.112 | 0.111 |
| TCGA-C5-A1MH | high | 0 | 0.061 | 0.046 | 0 | 0.039 | 0.049 | 0.127 | 0.084 | 0.116 | 0.074 | 0.15 | 0.049 | 0 | 0.028 | 0.031 | 0.237 | 0.163 | 0.052 | 0.197 | 0.137 | 0.1 | 0.027 | 0.101 | 0.107 |
| TCGA-ZJ-AAXU | high | 0 | 0.059 | 0.093 | 0 | 0.062 | 0.042 | 0.125 | 0.081 | 0.121 | 0.06 | 0.155 | 0.056 | 0 | 0.018 | 0.072 | 0.196 | 0.231 | 0.058 | 0.101 | 0.182 | 0.053 | 0.029 | 0.104 | 0.097 |
| TCGA-IR-A3LB | high | 0 | 0.078 | 0.041 | 0 | 0 | 0.06 | 0.13 | 0.088 | 0.111 | 0.131 | 0.105 | 0.064 | 0 | 0.044 | 0.088 | 0.187 | 0.099 | 0.041 | 0.114 | 0.157 | 0.096 | 0.045 | 0.098 | 0.087 |
| TCGA-R2-A69V | high | 0 | 0.035 | 0.083 | 0 | 0.029 | 0.052 | 0.134 | 0.071 | 0.128 | 0.074 | 0.118 | 0.028 | 0 | 0 | 0.077 | 0.298 | 0.196 | 0.05 | 0.191 | 0.163 | 0.042 | 0.034 | 0.103 | 0.103 |
| TCGA-VS-A9UB | high | 0 | 0.042 | 0.072 | 0 | 0.027 | 0.055 | 0.138 | 0.088 | 0.116 | 0.068 | 0.106 | 0.06 | 0 | 0.017 | 0.081 | 0.234 | 0.118 | 0.067 | 0.19 | 0.122 | 0.142 | 0.035 | 0.093 | 0.066 |
| TCGA-EA-A411 | high | 0 | 0.072 | 0.04 | 0 | 0.032 | 0.055 | 0.115 | 0.073 | 0.099 | 0.092 | 0.145 | 0.071 | 0 | 0.038 | 0.068 | 0.181 | 0.15 | 0.04 | 0.131 | 0.146 | 0.116 | 0.042 | 0.105 | 0.08 |
| TCGA-MA-AA3X | high | 0 | 0.058 | 0.056 | 0 | 0 | 0.05 | 0.117 | 0.09 | 0.121 | 0.077 | 0.132 | 0.058 | 0 | 0.054 | 0.076 | 0.21 | 0.103 | 0.057 | 0.221 | 0.152 | 0.117 | 0.037 | 0.047 | 0.09 |
| TCGA-IR-A3LH | high | 0 | 0.056 | 0.046 | 0.003 | 0.053 | 0.061 | 0.132 | 0.084 | 0.126 | 0.089 | 0.099 | 0.051 | 0 | 0.031 | 0 | 0.263 | 0.214 | 0.056 | 0.201 | 0.111 | 0.074 | 0.035 | 0.095 | 0.093 |
| TCGA-C5-A1MJ | high | 0 | 0.072 | 0.054 | 0 | 0 | 0.058 | 0.123 | 0.097 | 0.109 | 0.095 | 0.11 | 0.064 | 0 | 0.047 | 0.089 | 0.272 | 0.111 | 0.069 | 0.17 | 0.104 | 0.093 | 0.025 | 0.096 | 0.066 |
| TCGA-EA-A1QS | high | 0 | 0.068 | 0.037 | 0 | 0.017 | 0.05 | 0.119 | 0.087 | 0.123 | 0.073 | 0.09 | 0.053 | 0 | 0.067 | 0.077 | 0.245 | 0.106 | 0.091 | 0.155 | 0.124 | 0.077 | 0.06 | 0.1 | 0.088 |
| TCGA-DG-A2KK | high | 0 | 0.069 | 0.049 | 0 | 0 | 0.045 | 0.134 | 0.102 | 0.123 | 0.116 | 0.149 | 0.051 | 0 | 0.035 | 0.081 | 0.176 | 0.097 | 0.053 | 0.145 | 0.132 | 0.124 | 0.033 | 0.078 | 0.106 |
| TCGA-VS-A94W | high | 0 | 0.058 | 0.064 | 0 | 0.053 | 0.058 | 0.135 | 0.075 | 0.111 | 0.129 | 0.085 | 0.038 | 0 | 0.028 | 0.085 | 0.231 | 0.169 | 0.055 | 0.143 | 0.13 | 0.097 | 0.006 | 0.114 | 0.115 |
| TCGA-C5-A1BF | high | 0 | 0.056 | 0.062 | 0 | 0 | 0.061 | 0.13 | 0.079 | 0.132 | 0.117 | 0.107 | 0.06 | 0 | 0.031 | 0.073 | 0.133 | 0.133 | 0.059 | 0.137 | 0.176 | 0.144 | 0.044 | 0.085 | 0.084 |
| TCGA-C5-A1BQ | high | 0 | 0.055 | 0.051 | 0 | 0.004 | 0.052 | 0.139 | 0.1 | 0.112 | 0.089 | 0.124 | 0.085 | 0 | 0.021 | 0.079 | 0.218 | 0.05 | 0.074 | 0.182 | 0.085 | 0.033 | 0.062 | 0.091 | 0.136 |
| TCGA-ZJ-AAX8 | high | 0 | 0.068 | 0.073 | 0 | 0.049 | 0.06 | 0.136 | 0.079 | 0.113 | 0.034 | 0.142 | 0.059 | 0 | 0.036 | 0.073 | 0.22 | 0.163 | 0.037 | 0.133 | 0.134 | 0.102 | 0.035 | 0.1 | 0.097 |
| TCGA-EA-A556 | high | 0 | 0.081 | 0.077 | 0 | 0 | 0.041 | 0.119 | 0.094 | 0.119 | 0.095 | 0.087 | 0.047 | 0 | 0.048 | 0.087 | 0.152 | 0.132 | 0.038 | 0.103 | 0.175 | 0.118 | 0.044 | 0.119 | 0.074 |
| TCGA-ZJ-AAXN | high | 0 | 0.069 | 0.016 | 0 | 0 | 0.063 | 0.13 | 0.092 | 0.119 | 0.129 | 0.16 | 0.075 | 0 | 0.026 | 0.061 | 0.161 | 0.074 | 0.038 | 0.122 | 0.182 | 0.121 | 0.054 | 0.081 | 0.112 |
| TCGA-DS-A0VK | high | 0 | 0.082 | 0.063 | 0 | 0 | 0.053 | 0.068 | 0.087 | 0.121 | 0.134 | 0.084 | 0.081 | 0 | 0.066 | 0.057 | 0.208 | 0.143 | 0.045 | 0.132 | 0.187 | 0.078 | 0.061 | 0.088 | 0.086 |
| TCGA-HM-A4S6 | high | 0 | 0.081 | 0.052 | 0 | 0.009 | 0.057 | 0.14 | 0.091 | 0.077 | 0.113 | 0.073 | 0.068 | 0 | 0.021 | 0.092 | 0.226 | 0.072 | 0.05 | 0.192 | 0.13 | 0.036 | 0.055 | 0.086 | 0.115 |
| TCGA-DS-A1O9 | high | 0 | 0.072 | 0.041 | 0 | 0 | 0.057 | 0.142 | 0.117 | 0.117 | 0.013 | 0.132 | 0.06 | 0 | 0.041 | 0.065 | 0.218 | 0.097 | 0.068 | 0.219 | 0.107 | 0.106 | 0.052 | 0.046 | 0.099 |
| TCGA-EK-A2RN | high | 0 | 0.047 | 0.089 | 0 | 0.011 | 0.08 | 0.157 | 0.111 | 0.125 | 0.126 | 0.021 | 0.062 | 0.008 | 0 | 0.011 | 0.164 | 0.053 | 0.081 | 0.224 | 0.087 | 0.062 | 0 | 0.114 | 0.131 |
| TCGA-C5-A7X5 | high | 0 | 0.074 | 0.039 | 0 | 0 | 0.054 | 0.127 | 0.108 | 0.115 | 0.151 | 0.091 | 0.012 | 0.01 | 0.054 | 0.055 | 0.193 | 0.054 | 0.102 | 0.168 | 0.014 | 0.125 | 0.06 | 0.102 | 0.042 |
| TCGA-C5-A1MF | high | 0 | 0 | 0.054 | 0 | 0 | 0.052 | 0.04 | 0.122 | 0.143 | 0.08 | 0.113 | 0.07 | 0 | 0.025 | 0.098 | 0.194 | 0.152 | 0.061 | 0.184 | 0.145 | 0.071 | 0.041 | 0.098 | 0.124 |
| TCGA-VS-AA62 | high | 0 | 0.045 | 0.054 | 0 | 0.051 | 0.055 | 0.134 | 0.09 | 0.119 | 0.12 | 0.07 | 0.056 | 0 | 0.024 | 0.088 | 0.219 | 0.122 | 0.079 | 0.172 | 0.168 | 0.04 | 0.039 | 0.102 | 0.103 |
| TCGA-VS-A9UH | high | 0 | 0.054 | 0.072 | 0.006 | 0.048 | 0.041 | 0.138 | 0.112 | 0.122 | 0.01 | 0.079 | 0.056 | 0 | 0.051 | 0.081 | 0.222 | 0.133 | 0.063 | 0.212 | 0.15 | 0.034 | 0.031 | 0.099 | 0.105 |
| TCGA-JW-A5VJ | high | 0 | 0.079 | 0.056 | 0 | 0.023 | 0.057 | 0.127 | 0.088 | 0.117 | 0.07 | 0.085 | 0.105 | 0 | 0.056 | 0.014 | 0.167 | 0.183 | 0.054 | 0.139 | 0.132 | 0.075 | 0.029 | 0.092 | 0.112 |
| TCGA-JW-A5VH | high | 0 | 0.116 | 0.109 | 0 | 0 | 0 | 0.054 | 0.102 | 0.121 | 0.117 | 0.049 | 0.05 | 0.005 | 0.063 | 0.098 | 0.12 | 0.062 | 0.055 | 0.096 | 0.183 | 0.162 | 0.071 | 0.057 | 0.045 |
| TCGA-C5-A7CJ | high | 0 | 0.074 | 0.062 | 0 | 0.057 | 0.057 | 0.132 | 0.058 | 0.121 | 0.06 | 0.095 | 0.07 | 0 | 0.003 | 0.072 | 0.222 | 0.173 | 0.045 | 0.141 | 0.17 | 0.14 | 0.026 | 0.1 | 0.092 |
| TCGA-EA-A6QX | high | 0 | 0.07 | 0.052 | 0 | 0.035 | 0.059 | 0.13 | 0.12 | 0.122 | 0.099 | 0 | 0.089 | 0 | 0.05 | 0.083 | 0.203 | 0.134 | 0.053 | 0.181 | 0.073 | 0.033 | 0 | 0.103 | 0.118 |
| TCGA-VS-A9UY | high | 0 | 0.081 | 0.033 | 0 | 0.01 | 0.064 | 0.143 | 0.103 | 0.127 | 0.099 | 0.124 | 0.066 | 0 | 0.014 | 0.013 | 0.223 | 0.091 | 0.067 | 0.186 | 0.123 | 0.153 | 0.047 | 0.022 | 0.11 |
| TCGA-EK-A2RL | high | 0 | 0.004 | 0.07 | 0 | 0 | 0.044 | 0.032 | 0.13 | 0.125 | 0.164 | 0.082 | 0.098 | 0.019 | 0.083 | 0.105 | 0.072 | 0.135 | 0.081 | 0.096 | 0.066 | 0.142 | 0.033 | 0.06 | 0.098 |
| TCGA-4J-AA1J | high | 0 | 0.086 | 0.055 | 0 | 0 | 0.045 | 0.133 | 0.074 | 0.12 | 0.125 | 0.124 | 0.06 | 0 | 0.031 | 0.073 | 0.195 | 0.052 | 0.049 | 0.102 | 0.133 | 0.16 | 0.059 | 0.092 | 0.076 |
| TCGA-LP-A4AU | high | 0 | 0.052 | 0.057 | 0 | 0 | 0.044 | 0.119 | 0.092 | 0.107 | 0.153 | 0.136 | 0.07 | 0 | 0.051 | 0.083 | 0.05 | 0.123 | 0.027 | 0.097 | 0.158 | 0.142 | 0.045 | 0.103 | 0.116 |
| TCGA-DS-A7WF | high | 0 | 0.082 | 0.024 | 0 | 0.008 | 0.062 | 0.102 | 0.13 | 0.087 | 0.116 | 0.076 | 0.077 | 0 | 0.077 | 0.076 | 0.145 | 0.084 | 0.027 | 0.133 | 0.143 | 0.056 | 0.054 | 0.11 | 0.071 |
| TCGA-EK-A2RM | high | 0 | 0.069 | 0.01 | 0 | 0 | 0.068 | 0.135 | 0.097 | 0.115 | 0.144 | 0.137 | 0.073 | 0 | 0 | 0.082 | 0.193 | 0.042 | 0.055 | 0.175 | 0.154 | 0.114 | 0.032 | 0.06 | 0.061 |
| TCGA-C5-A8YR | high | 0 | 0.057 | 0.069 | 0 | 0.013 | 0.068 | 0.125 | 0.081 | 0.107 | 0.141 | 0.11 | 0.058 | 0 | 0.053 | 0 | 0.185 | 0.057 | 0.05 | 0.208 | 0.185 | 0.04 | 0.056 | 0.087 | 0.112 |
| TCGA-FU-A5XV | high | 0 | 0.057 | 0.062 | 0 | 0.03 | 0.05 | 0.132 | 0.087 | 0.131 | 0.084 | 0.152 | 0.079 | 0 | 0.023 | 0.052 | 0.189 | 0.161 | 0.04 | 0.122 | 0.14 | 0.116 | 0.04 | 0.06 | 0.119 |
| TCGA-HG-A2PA | high | 0 | 0.049 | 0.064 | 0 | 0.035 | 0.05 | 0.135 | 0.075 | 0.126 | 0.099 | 0.147 | 0.061 | 0 | 0.002 | 0.07 | 0.19 | 0.139 | 0.043 | 0.09 | 0.167 | 0.152 | 0.04 | 0.099 | 0.092 |
| TCGA-LP-A5U3 | high | 0 | 0.045 | 0.035 | 0 | 0 | 0.055 | 0.134 | 0.121 | 0.112 | 0.144 | 0.135 | 0.074 | 0 | 0.006 | 0.047 | 0.246 | 0.083 | 0.052 | 0.186 | 0.138 | 0.089 | 0.042 | 0.061 | 0.122 |
| TCGA-Q1-A73Q | high | 0 | 0.077 | 0.052 | 0 | 0.053 | 0.05 | 0.068 | 0.095 | 0.113 | 0.094 | 0.133 | 0.068 | 0 | 0.041 | 0.076 | 0.174 | 0.183 | 0.051 | 0.057 | 0.152 | 0.124 | 0.041 | 0.106 | 0.105 |
| TCGA-VS-A957 | high | 0 | 0.056 | 0.045 | 0 | 0 | 0.053 | 0.114 | 0.101 | 0.104 | 0.162 | 0.138 | 0.077 | 0 | 0 | 0.019 | 0.205 | 0.109 | 0.095 | 0.133 | 0.064 | 0.138 | 0.06 | 0.095 | 0.11 |
| TCGA-VS-A8EJ | high | 0 | 0.1 | 0.063 | 0 | 0 | 0.057 | 0.12 | 0.129 | 0.132 | 0.08 | 0 | 0.048 | 0.007 | 0.044 | 0.105 | 0.168 | 0.134 | 0.051 | 0.143 | 0.153 | 0.053 | 0 | 0.083 | 0.114 |
| TCGA-VS-A9V0 | high | 0 | 0.076 | 0.054 | 0 | 0 | 0.054 | 0.023 | 0.113 | 0.106 | 0.183 | 0.099 | 0.072 | 0.014 | 0.073 | 0.11 | 0.148 | 0.013 | 0.092 | 0.076 | 0.09 | 0.067 | 0.062 | 0.108 | 0.114 |
| TCGA-IR-A3LK | high | 0 | 0.061 | 0.026 | 0 | 0 | 0.063 | 0.134 | 0.113 | 0.119 | 0.025 | 0.171 | 0.056 | 0 | 0.033 | 0.064 | 0.262 | 0.083 | 0.073 | 0.179 | 0.173 | 0.082 | 0.051 | 0.079 | 0.107 |
| TCGA-C5-A7XC | high | 0 | 0.048 | 0.061 | 0 | 0.038 | 0.047 | 0.138 | 0.051 | 0.114 | 0.113 | 0.121 | 0.063 | 0 | 0.013 | 0.082 | 0.202 | 0.167 | 0.042 | 0.118 | 0.156 | 0.12 | 0.041 | 0.104 | 0.103 |
| TCGA-Q1-A6DT | high | 0 | 0.062 | 0.061 | 0 | 0 | 0.051 | 0.131 | 0.089 | 0.125 | 0.109 | 0.128 | 0.067 | 0 | 0 | 0.059 | 0.217 | 0.113 | 0.043 | 0.146 | 0.171 | 0.161 | 0.036 | 0.07 | 0.069 |
| TCGA-GH-A9DA | high | 0 | 0.056 | 0.059 | 0 | 0 | 0.05 | 0.127 | 0.096 | 0.123 | 0.121 | 0.086 | 0.065 | 0 | 0.073 | 0.072 | 0.198 | 0.069 | 0.085 | 0.163 | 0.149 | 0.066 | 0.051 | 0.094 | 0.096 |
| TCGA-DS-A0VN | high | 0 | 0.062 | 0.067 | 0.002 | 0.033 | 0.062 | 0.135 | 0.092 | 0.128 | 0.037 | 0.082 | 0.054 | 0 | 0.028 | 0.075 | 0.266 | 0.151 | 0.042 | 0.175 | 0.196 | 0.072 | 0.033 | 0.075 | 0.119 |
| TCGA-VS-A94X | high | 0 | 0.054 | 0.078 | 0 | 0.007 | 0.057 | 0.095 | 0.102 | 0.107 | 0.134 | 0.103 | 0.073 | 0 | 0.067 | 0.059 | 0.17 | 0.095 | 0.075 | 0.14 | 0.14 | 0.097 | 0.027 | 0.108 | 0.111 |
| TCGA-VS-A8Q8 | high | 0 | 0.031 | 0.071 | 0 | 0.007 | 0.058 | 0.128 | 0.085 | 0.128 | 0.105 | 0.146 | 0.061 | 0 | 0.038 | 0.006 | 0.232 | 0.151 | 0.076 | 0.166 | 0.156 | 0.135 | 0.03 | 0.042 | 0.114 |
| TCGA-C5-A7X3 | high | 0 | 0.055 | 0.041 | 0 | 0.04 | 0.043 | 0.119 | 0.094 | 0.136 | 0.068 | 0.084 | 0.064 | 0 | 0.054 | 0.075 | 0.204 | 0.217 | 0.053 | 0.168 | 0.151 | 0.04 | 0.051 | 0.103 | 0.089 |
| TCGA-ZJ-AAXJ | high | 0 | 0.096 | 0.039 | 0 | 0.035 | 0.061 | 0.137 | 0.104 | 0.136 | 0.079 | 0.116 | 0.101 | 0 | 0.013 | 0.014 | 0.237 | 0.111 | 0.043 | 0.087 | 0.169 | 0.057 | 0.056 | 0.084 | 0.13 |
| TCGA-JW-A5VL | high | 0 | 0.074 | 0.031 | 0 | 0.004 | 0.061 | 0.133 | 0.1 | 0.138 | 0.069 | 0.113 | 0.065 | 0 | 0.047 | 0.073 | 0.22 | 0.029 | 0.062 | 0.201 | 0.136 | 0.027 | 0.044 | 0.087 | 0.138 |
| TCGA-UC-A7PG | high | 0 | 0.013 | 0.061 | 0 | 0.014 | 0.051 | 0.049 | 0.091 | 0.093 | 0.165 | 0.182 | 0.09 | 0 | 0.077 | 0.01 | 0.186 | 0.102 | 0.048 | 0.125 | 0.142 | 0.154 | 0.038 | 0.092 | 0.116 |
| TCGA-C5-A1M7 | high | 0 | 0.054 | 0.066 | 0 | 0.052 | 0.05 | 0.122 | 0.08 | 0.118 | 0.1 | 0.136 | 0.05 | 0 | 0.003 | 0.092 | 0.171 | 0.206 | 0.048 | 0.103 | 0.137 | 0.156 | 0.014 | 0.097 | 0.086 |
| TCGA-VS-A953 | high | 0 | 0.087 | 0.035 | 0 | 0 | 0.059 | 0.138 | 0.115 | 0.126 | 0.03 | 0.129 | 0.077 | 0 | 0.055 | 0.079 | 0.196 | 0.091 | 0.082 | 0.161 | 0.124 | 0.092 | 0.054 | 0.047 | 0.1 |
| TCGA-C5-A7CK | high | 0 | 0.088 | 0.016 | 0 | 0.017 | 0.05 | 0.068 | 0.093 | 0.133 | 0.09 | 0.111 | 0.087 | 0 | 0.067 | 0.061 | 0.178 | 0.142 | 0.063 | 0.12 | 0.137 | 0.103 | 0.054 | 0.089 | 0.102 |
| TCGA-EK-A3GM | high | 0 | 0.088 | 0.03 | 0 | 0 | 0 | 0.14 | 0.113 | 0.099 | 0.17 | 0.106 | 0.088 | 0.021 | 0.087 | 0.018 | 0.086 | 0.033 | 0.087 | 0.098 | 0.147 | 0.062 | 0.065 | 0.102 | 0.044 |
| TCGA-MA-AA3Y | high | 0 | 0.039 | 0.061 | 0 | 0.032 | 0.053 | 0.128 | 0.115 | 0.136 | 0.06 | 0.099 | 0.071 | 0 | 0.027 | 0.07 | 0.239 | 0.16 | 0.061 | 0.175 | 0.167 | 0.106 | 0.031 | 0.077 | 0.1 |
| TCGA-ZJ-AAXI | high | 0 | 0.092 | 0.02 | 0 | 0 | 0.065 | 0.141 | 0.102 | 0.122 | 0.069 | 0.084 | 0.094 | 0 | 0.015 | 0.104 | 0.231 | 0.079 | 0.082 | 0.108 | 0.135 | 0.02 | 0.062 | 0.095 | 0.127 |
| TCGA-VS-A9UM | high | 0 | 0.067 | 0.064 | 0 | 0.054 | 0.047 | 0.138 | 0.07 | 0.095 | 0.046 | 0.1 | 0.065 | 0 | 0.03 | 0.07 | 0.203 | 0.171 | 0.043 | 0.156 | 0.144 | 0.116 | 0.04 | 0.095 | 0.114 |
| TCGA-IR-A3LL | low | 0 | 0.052 | 0.065 | 0.006 | 0.01 | 0.054 | 0.135 | 0.09 | 0.132 | 0.034 | 0.072 | 0.035 | 0 | 0.018 | 0.096 | 0.254 | 0.18 | 0.064 | 0.191 | 0.117 | 0.1 | 0.033 | 0.069 | 0.125 |
| TCGA-VS-A9UP | low | 0.015 | 0.044 | 0.076 | 0 | 0.023 | 0.04 | 0 | 0 | 0.132 | 0.115 | 0.117 | 0.088 | 0 | 0.054 | 0.077 | 0.122 | 0.196 | 0.054 | 0.057 | 0.153 | 0.144 | 0.069 | 0.12 | 0.074 |
| TCGA-VS-A94Z | low | 0 | 0.062 | 0.063 | 0.008 | 0.057 | 0.063 | 0.146 | 0.091 | 0.12 | 0.016 | 0.097 | 0.072 | 0 | 0.007 | 0.088 | 0.178 | 0.126 | 0.061 | 0.167 | 0.186 | 0.038 | 0.05 | 0.108 | 0.117 |
| TCGA-MA-AA41 | low | 0 | 0.088 | 0.056 | 0 | 0 | 0.054 | 0.133 | 0.084 | 0.118 | 0.096 | 0.131 | 0.074 | 0 | 0 | 0.084 | 0.219 | 0.13 | 0.068 | 0.116 | 0.182 | 0.076 | 0.027 | 0.089 | 0.134 |
| TCGA-FU-A23K | low | 0 | 0.014 | 0.045 | 0 | 0.033 | 0.048 | 0.018 | 0.108 | 0.13 | 0.053 | 0.162 | 0.056 | 0 | 0.064 | 0.09 | 0.248 | 0.184 | 0.026 | 0.164 | 0.187 | 0.046 | 0.05 | 0.102 | 0.077 |
| TCGA-C5-A7UH | low | 0 | 0.075 | 0.037 | 0 | 0.036 | 0.049 | 0.148 | 0.095 | 0.112 | 0.078 | 0.092 | 0.066 | 0 | 0.054 | 0.074 | 0.195 | 0.114 | 0.059 | 0.168 | 0.164 | 0.131 | 0.041 | 0.044 | 0.091 |
| TCGA-DS-A0VM | low | 0 | 0.063 | 0.061 | 0 | 0 | 0.056 | 0.135 | 0.135 | 0.133 | 0.068 | 0.088 | 0.079 | 0 | 0 | 0.049 | 0.272 | 0.121 | 0.039 | 0.153 | 0.164 | 0.103 | 0.047 | 0.067 | 0.099 |
| TCGA-IR-A3LC | low | 0 | 0.079 | 0.048 | 0 | 0 | 0.057 | 0.131 | 0.099 | 0.113 | 0.106 | 0.146 | 0.076 | 0 | 0.018 | 0.054 | 0.185 | 0.069 | 0.068 | 0.155 | 0.178 | 0.043 | 0.059 | 0.101 | 0.102 |
| TCGA-EK-A2H0 | low | 0 | 0.074 | 0.079 | 0 | 0 | 0.048 | 0.13 | 0.121 | 0.118 | 0.098 | 0.144 | 0.079 | 0 | 0.032 | 0.021 | 0.212 | 0.013 | 0.075 | 0.151 | 0.12 | 0.076 | 0.051 | 0.076 | 0.124 |
| TCGA-EX-A449 | low | 0 | 0.076 | 0.044 | 0 | 0.005 | 0.034 | 0.13 | 0.101 | 0.098 | 0.14 | 0.082 | 0.091 | 0 | 0.068 | 0.073 | 0.132 | 0.139 | 0.069 | 0.092 | 0.175 | 0.086 | 0.075 | 0.065 | 0.087 |
| TCGA-VS-A9UI | low | 0 | 0.081 | 0.026 | 0 | 0.039 | 0.049 | 0.119 | 0.097 | 0.099 | 0.111 | 0.122 | 0.072 | 0 | 0 | 0.09 | 0.201 | 0.104 | 0.055 | 0.169 | 0.108 | 0.059 | 0.047 | 0.098 | 0.144 |
| TCGA-C5-A7CO | low | 0 | 0.073 | 0.035 | 0 | 0 | 0.069 | 0.061 | 0.096 | 0.122 | 0.08 | 0.114 | 0.078 | 0 | 0.069 | 0.084 | 0.195 | 0.143 | 0.06 | 0.122 | 0.089 | 0.117 | 0.054 | 0.092 | 0.123 |
| TCGA-C5-A2LS | low | 0 | 0 | 0.082 | 0 | 0 | 0.06 | 0.045 | 0.132 | 0.131 | 0.143 | 0.088 | 0.078 | 0.02 | 0.011 | 0.108 | 0.162 | 0.151 | 0.084 | 0.029 | 0.144 | 0.101 | 0.039 | 0.129 | 0.094 |
| TCGA-C5-A1BI | low | 0 | 0.066 | 0.072 | 0 | 0.06 | 0.061 | 0.131 | 0.086 | 0.1 | 0.069 | 0.087 | 0.064 | 0 | 0.015 | 0.077 | 0.228 | 0.172 | 0.032 | 0.138 | 0.189 | 0.102 | 0.023 | 0.09 | 0.1 |
| TCGA-VS-A9UQ | low | 0 | 0.074 | 0.066 | 0 | 0 | 0.041 | 0.128 | 0.09 | 0.116 | 0.139 | 0.069 | 0.065 | 0 | 0.027 | 0.093 | 0.173 | 0.156 | 0.058 | 0.067 | 0.166 | 0.112 | 0.03 | 0.122 | 0.086 |
| TCGA-EA-A5FO | low | 0.002 | 0.117 | 0.017 | 0 | 0 | 0.065 | 0.115 | 0.114 | 0.095 | 0.141 | 0.116 | 0.089 | 0 | 0.103 | 0 | 0.027 | 0.116 | 0.014 | 0.001 | 0.102 | 0.131 | 0.064 | 0.103 | 0.116 |
| TCGA-C5-A1BK | low | 0 | 0.061 | 0.059 | 0 | 0.028 | 0.06 | 0.137 | 0.073 | 0.086 | 0.094 | 0.13 | 0.057 | 0 | 0.009 | 0.074 | 0.218 | 0.211 | 0.003 | 0.14 | 0.144 | 0.097 | 0.024 | 0.101 | 0.112 |
| TCGA-HM-A3JK | low | 0 | 0.06 | 0.065 | 0 | 0.018 | 0.042 | 0.126 | 0.073 | 0.115 | 0.109 | 0.124 | 0.069 | 0 | 0.031 | 0.09 | 0.216 | 0.143 | 0.059 | 0.15 | 0.13 | 0.073 | 0.041 | 0.115 | 0.08 |
| TCGA-C5-A2LY | low | 0 | 0.048 | 0.081 | 0 | 0 | 0.04 | 0.129 | 0.092 | 0.126 | 0.105 | 0.074 | 0.057 | 0 | 0.012 | 0.078 | 0.261 | 0.195 | 0.067 | 0.179 | 0.126 | 0.096 | 0.03 | 0.056 | 0.126 |
| TCGA-C5-A2LZ | low | 0 | 0.063 | 0.03 | 0 | 0.02 | 0.048 | 0.145 | 0.076 | 0.099 | 0.128 | 0.135 | 0.074 | 0 | 0.028 | 0.065 | 0.209 | 0.163 | 0.054 | 0.116 | 0.139 | 0.102 | 0.044 | 0.085 | 0.112 |
| TCGA-ZJ-AAX4 | low | 0 | 0.065 | 0.04 | 0 | 0.047 | 0.054 | 0.133 | 0.067 | 0.121 | 0.018 | 0.104 | 0.071 | 0 | 0.022 | 0.087 | 0.245 | 0.177 | 0.056 | 0.19 | 0.103 | 0.129 | 0.027 | 0.084 | 0.109 |
| TCGA-DR-A0ZL | low | 0 | 0.072 | 0.049 | 0 | 0.001 | 0.058 | 0.125 | 0.085 | 0.115 | 0.108 | 0.135 | 0.088 | 0 | 0.051 | 0.068 | 0.134 | 0.201 | 0.029 | 0.078 | 0.107 | 0.109 | 0.034 | 0.101 | 0.084 |
| TCGA-ZJ-A8QO | low | 0 | 0.073 | 0.044 | 0 | 0.079 | 0.071 | 0.076 | 0.08 | 0.132 | 0.081 | 0.1 | 0.066 | 0 | 0.017 | 0.072 | 0.263 | 0.173 | 0.06 | 0.13 | 0.147 | 0.091 | 0.024 | 0.116 | 0.112 |
| TCGA-EA-A410 | low | 0 | 0.06 | 0.113 | 0 | 0 | 0.062 | 0.06 | 0.128 | 0.127 | 0.003 | 0.099 | 0.076 | 0 | 0.046 | 0.103 | 0.166 | 0.118 | 0.036 | 0.16 | 0.125 | 0.106 | 0.065 | 0.081 | 0.136 |
| TCGA-Q1-A5R3 | low | 0 | 0.005 | 0.055 | 0.002 | 0 | 0.065 | 0.038 | 0.126 | 0.135 | 0.144 | 0.128 | 0.1 | 0 | 0.051 | 0.003 | 0.219 | 0.118 | 0.067 | 0.151 | 0.17 | 0.04 | 0.021 | 0.145 | 0.028 |
| TCGA-MA-AA3W | low | 0 | 0.066 | 0.068 | 0 | 0.054 | 0.059 | 0.137 | 0.08 | 0.12 | 0.077 | 0.091 | 0.045 | 0 | 0.008 | 0.077 | 0.254 | 0.197 | 0.051 | 0.125 | 0.127 | 0.104 | 0.035 | 0.095 | 0.102 |
| TCGA-C5-A902 | low | 0 | 0.075 | 0.108 | 0 | 0.004 | 0.052 | 0.139 | 0.09 | 0.101 | 0.125 | 0.141 | 0.062 | 0 | 0.044 | 0 | 0.118 | 0.143 | 0.053 | 0.102 | 0.173 | 0.083 | 0.06 | 0.102 | 0.124 |
| TCGA-EA-A3HU | low | 0 | 0.044 | 0.065 | 0.005 | 0.048 | 0.059 | 0.137 | 0.11 | 0.13 | 0 | 0.114 | 0.071 | 0 | 0 | 0.077 | 0.191 | 0.176 | 0.058 | 0.162 | 0.141 | 0.068 | 0.066 | 0.103 | 0.119 |
| TCGA-MU-A8JM | low | 0 | 0.057 | 0.037 | 0 | 0.028 | 0.064 | 0.135 | 0.083 | 0.123 | 0.064 | 0.106 | 0.063 | 0 | 0.031 | 0.079 | 0.25 | 0.123 | 0.063 | 0.123 | 0.177 | 0.106 | 0.045 | 0.089 | 0.108 |
| TCGA-MA-AA42 | low | 0 | 0.027 | 0.027 | 0.022 | 0.067 | 0.063 | 0.14 | 0.092 | 0.133 | 0.03 | 0.067 | 0.041 | 0 | 0 | 0.08 | 0.3 | 0.166 | 0.083 | 0.207 | 0.126 | 0.024 | 0.044 | 0.09 | 0.132 |
| TCGA-C5-A1M5 | low | 0 | 0.068 | 0.051 | 0 | 0.039 | 0.05 | 0.125 | 0.082 | 0.117 | 0.104 | 0.111 | 0.064 | 0 | 0.02 | 0.092 | 0.223 | 0.191 | 0.038 | 0.102 | 0.126 | 0.093 | 0.024 | 0.099 | 0.109 |
| TCGA-ZJ-AAXA | low | 0 | 0.093 | 0 | 0 | 0 | 0.053 | 0.062 | 0.112 | 0.122 | 0.067 | 0.172 | 0.1 | 0.012 | 0.1 | 0.021 | 0.251 | 0.08 | 0.055 | 0.188 | 0.07 | 0.091 | 0.042 | 0.057 | 0.099 |
| TCGA-FU-A3YQ | low | 0 | 0.063 | 0.062 | 0 | 0.06 | 0.048 | 0.13 | 0.071 | 0.122 | 0.042 | 0.127 | 0.059 | 0 | 0.017 | 0.08 | 0.232 | 0.173 | 0.052 | 0.147 | 0.178 | 0.121 | 0.023 | 0.087 | 0.086 |
| TCGA-C5-A1M8 | low | 0 | 0.082 | 0.06 | 0 | 0 | 0.05 | 0.066 | 0.069 | 0.115 | 0.114 | 0.118 | 0.07 | 0 | 0.052 | 0.094 | 0.197 | 0.128 | 0.056 | 0.133 | 0.128 | 0.133 | 0.034 | 0.076 | 0.102 |
| TCGA-ZX-AA5X | low | 0 | 0.058 | 0.072 | 0 | 0.03 | 0.051 | 0.133 | 0.071 | 0.114 | 0.086 | 0.094 | 0.047 | 0 | 0.031 | 0.087 | 0.223 | 0.193 | 0.053 | 0.149 | 0.148 | 0.116 | 0.024 | 0.112 | 0.072 |
| TCGA-IR-A3LF | low | 0 | 0.079 | 0.035 | 0 | 0.015 | 0.049 | 0.128 | 0.079 | 0.112 | 0.121 | 0.098 | 0.059 | 0 | 0.051 | 0.083 | 0.197 | 0.196 | 0.061 | 0.09 | 0.08 | 0.112 | 0.031 | 0.114 | 0.076 |
| TCGA-IR-A3LA | low | 0 | 0.07 | 0.045 | 0 | 0.016 | 0.067 | 0.129 | 0.107 | 0.125 | 0.09 | 0.119 | 0.036 | 0 | 0.024 | 0.091 | 0.201 | 0.098 | 0.065 | 0.155 | 0.136 | 0.047 | 0.058 | 0.1 | 0.081 |
| TCGA-FU-A40J | low | 0 | 0.077 | 0.041 | 0 | 0 | 0.047 | 0.135 | 0.087 | 0.133 | 0.095 | 0.105 | 0.061 | 0 | 0.039 | 0.079 | 0.143 | 0.15 | 0.043 | 0.091 | 0.112 | 0.165 | 0.042 | 0.092 | 0.101 |
| TCGA-JX-A3Q0 | low | 0 | 0.053 | 0.038 | 0 | 0.025 | 0.054 | 0.138 | 0.104 | 0.116 | 0.089 | 0.154 | 0.064 | 0 | 0.042 | 0.072 | 0.236 | 0.062 | 0.031 | 0.187 | 0.081 | 0.03 | 0.053 | 0.091 | 0.121 |
| TCGA-C5-A1ME | low | 0 | 0.002 | 0.075 | 0 | 0 | 0.049 | 0.049 | 0.09 | 0.127 | 0.128 | 0.117 | 0.065 | 0 | 0.07 | 0.084 | 0.145 | 0.156 | 0.086 | 0.136 | 0.156 | 0.086 | 0.051 | 0.09 | 0.082 |
| TCGA-VS-A9UO | low | 0 | 0.017 | 0.084 | 0 | 0 | 0.059 | 0.053 | 0.127 | 0.102 | 0.16 | 0.115 | 0.093 | 0.016 | 0.033 | 0.089 | 0.043 | 0.093 | 0.105 | 0 | 0.17 | 0.083 | 0.073 | 0.086 | 0.1 |
| TCGA-BI-A20A | low | 0 | 0.078 | 0.059 | 0 | 0.044 | 0.054 | 0.132 | 0.097 | 0.094 | 0.089 | 0.131 | 0.067 | 0 | 0.032 | 0.025 | 0.23 | 0.164 | 0.072 | 0.145 | 0.104 | 0.039 | 0.038 | 0.113 | 0.094 |
| TCGA-VS-A9UC | low | 0 | 0.089 | 0.007 | 0 | 0.015 | 0.046 | 0.133 | 0.098 | 0.08 | 0.114 | 0.118 | 0.075 | 0 | 0 | 0.096 | 0.182 | 0.126 | 0.046 | 0.174 | 0.115 | 0.102 | 0.061 | 0.07 | 0.124 |
| TCGA-C5-A7UI | low | 0 | 0.046 | 0.085 | 0 | 0.031 | 0.051 | 0.139 | 0.108 | 0.092 | 0.038 | 0.092 | 0.048 | 0 | 0.02 | 0.093 | 0.19 | 0.151 | 0.031 | 0.151 | 0.202 | 0.071 | 0.043 | 0.1 | 0.11 |
| TCGA-C5-A1BL | low | 0 | 0.047 | 0.07 | 0 | 0.025 | 0.061 | 0.135 | 0.101 | 0.129 | 0.031 | 0.09 | 0.066 | 0 | 0.023 | 0.06 | 0.27 | 0.138 | 0.041 | 0.151 | 0.165 | 0.108 | 0.047 | 0.078 | 0.115 |
| TCGA-DG-A2KJ | low | 0 | 0 | 0.102 | 0 | 0.002 | 0.039 | 0.035 | 0.08 | 0.12 | 0.108 | 0.112 | 0.048 | 0 | 0.102 | 0.099 | 0.263 | 0.213 | 0.062 | 0.161 | 0.188 | 0.046 | 0.044 | 0.071 | 0.086 |
| TCGA-VS-A958 | low | 0 | 0.054 | 0.065 | 0 | 0.038 | 0.049 | 0.139 | 0.084 | 0.102 | 0.09 | 0.114 | 0.046 | 0 | 0.003 | 0.08 | 0.22 | 0.183 | 0.032 | 0.159 | 0.129 | 0.119 | 0.059 | 0.075 | 0.103 |
| TCGA-EA-A5ZF | low | 0 | 0.068 | 0.033 | 0 | 0 | 0.042 | 0.119 | 0.099 | 0.112 | 0.103 | 0.1 | 0.052 | 0 | 0.066 | 0.072 | 0.153 | 0.166 | 0.05 | 0.147 | 0.156 | 0.124 | 0.055 | 0.097 | 0.043 |
| TCGA-MU-A51Y | low | 0 | 0.06 | 0.071 | 0 | 0.045 | 0.047 | 0.137 | 0.07 | 0.108 | 0.079 | 0.09 | 0.06 | 0 | 0.02 | 0.079 | 0.216 | 0.189 | 0.038 | 0.154 | 0.08 | 0.099 | 0.05 | 0.101 | 0.099 |
| TCGA-UC-A7PI | low | 0 | 0.087 | 0.015 | 0 | 0 | 0.046 | 0.119 | 0.106 | 0.106 | 0.149 | 0.109 | 0.079 | 0 | 0.051 | 0.073 | 0.13 | 0.108 | 0.08 | 0.114 | 0.086 | 0.133 | 0.046 | 0.079 | 0.084 |
| TCGA-VS-A952 | low | 0 | 0.081 | 0.037 | 0 | 0.043 | 0.058 | 0.131 | 0.101 | 0.063 | 0.137 | 0.096 | 0.088 | 0.011 | 0.002 | 0.089 | 0.141 | 0.217 | 0.084 | 0.06 | 0.114 | 0.059 | 0.034 | 0.124 | 0.057 |
| TCGA-ZJ-A8QQ | low | 0 | 0.077 | 0.069 | 0 | 0 | 0.048 | 0.129 | 0.133 | 0.127 | 0.12 | 0.13 | 0.081 | 0 | 0 | 0.005 | 0.217 | 0.076 | 0.071 | 0.151 | 0.141 | 0.119 | 0.041 | 0.085 | 0.109 |
| TCGA-Q1-A6DV | low | 0 | 0.055 | 0.043 | 0 | 0 | 0.051 | 0.129 | 0.095 | 0.117 | 0.095 | 0.174 | 0.05 | 0 | 0.014 | 0.091 | 0.126 | 0.193 | 0.071 | 0.007 | 0.19 | 0.173 | 0.059 | 0.116 | 0.042 |
| TCGA-HM-A6W2 | low | 0 | 0.098 | 0.08 | 0 | 0 | 0.037 | 0.049 | 0.104 | 0.126 | 0.032 | 0.133 | 0.075 | 0 | 0.062 | 0.032 | 0.133 | 0.184 | 0.04 | 0.18 | 0.152 | 0.135 | 0.052 | 0.097 | 0.05 |
| TCGA-C5-A2M1 | low | 0 | 0.054 | 0.082 | 0 | 0.018 | 0.059 | 0.133 | 0.101 | 0.121 | 0.09 | 0.12 | 0.067 | 0 | 0.001 | 0.071 | 0.2 | 0.158 | 0.065 | 0.17 | 0.146 | 0.08 | 0.035 | 0.087 | 0.072 |
| TCGA-FU-A2QG | low | 0 | 0.078 | 0.075 | 0 | 0 | 0.055 | 0.107 | 0.101 | 0.13 | 0.119 | 0.131 | 0.08 | 0 | 0.022 | 0.069 | 0.159 | 0.12 | 0.049 | 0.062 | 0.141 | 0.118 | 0.056 | 0.068 | 0.129 |
| TCGA-EK-A2IR | low | 0 | 0.094 | 0.002 | 0 | 0 | 0.071 | 0.064 | 0.103 | 0.148 | 0.075 | 0.098 | 0.032 | 0.001 | 0.077 | 0.078 | 0.166 | 0.044 | 0.123 | 0.151 | 0.103 | 0.066 | 0.07 | 0.108 | 0.079 |
| TCGA-EK-A2R7 | low | 0 | 0.051 | 0.064 | 0 | 0.04 | 0.05 | 0.131 | 0.094 | 0.12 | 0.065 | 0.14 | 0.048 | 0 | 0.026 | 0.087 | 0.181 | 0.218 | 0.025 | 0.111 | 0.141 | 0.089 | 0.031 | 0.108 | 0.098 |
| TCGA-VS-A9UR | low | 0.006 | 0.01 | 0.1 | 0 | 0 | 0.052 | 0.046 | 0.131 | 0.116 | 0.167 | 0.103 | 0.098 | 0.025 | 0.079 | 0 | 0.097 | 0.136 | 0.061 | 0.053 | 0.087 | 0.122 | 0.013 | 0.111 | 0.122 |
| TCGA-VS-A959 | low | 0 | 0.058 | 0.059 | 0 | 0.041 | 0.045 | 0.125 | 0.078 | 0.101 | 0.142 | 0.085 | 0.063 | 0 | 0.043 | 0.083 | 0.161 | 0.21 | 0.037 | 0.129 | 0.072 | 0.115 | 0.024 | 0.114 | 0.102 |
| TCGA-LP-A4AV | low | 0 | 0 | 0.073 | 0.002 | 0 | 0.045 | 0.032 | 0.112 | 0.127 | 0.113 | 0.083 | 0.049 | 0 | 0.098 | 0.027 | 0.271 | 0.203 | 0.062 | 0.211 | 0.112 | 0.006 | 0.071 | 0.054 | 0.167 |
| TCGA-EA-A78R | low | 0 | 0.046 | 0.068 | 0 | 0 | 0.038 | 0.123 | 0.091 | 0.108 | 0.075 | 0.144 | 0.06 | 0 | 0.056 | 0.083 | 0.229 | 0.178 | 0.035 | 0.157 | 0.17 | 0.123 | 0.022 | 0.071 | 0.084 |
| TCGA-JX-A3Q8 | low | 0 | 0.078 | 0.05 | 0 | 0 | 0.07 | 0.131 | 0.128 | 0.134 | 0.065 | 0.04 | 0.078 | 0.009 | 0.03 | 0.085 | 0.216 | 0.113 | 0.058 | 0.166 | 0.239 | 0.034 | 0.015 | 0.059 | 0.111 |
| TCGA-PN-A8MA | low | 0 | 0.05 | 0.055 | 0 | 0.033 | 0.051 | 0.12 | 0.091 | 0.115 | 0.058 | 0.134 | 0.058 | 0 | 0.045 | 0.109 | 0.161 | 0.19 | 0.044 | 0.125 | 0.159 | 0.076 | 0.055 | 0.095 | 0.095 |
| TCGA-EA-A43B | low | 0 | 0.059 | 0.074 | 0 | 0 | 0.045 | 0.135 | 0.1 | 0.119 | 0.146 | 0.094 | 0.054 | 0 | 0 | 0.076 | 0.17 | 0.137 | 0.054 | 0.159 | 0.15 | 0.094 | 0.041 | 0.097 | 0.072 |
| TCGA-IR-A3L7 | low | 0 | 0.083 | 0.013 | 0 | 0 | 0.032 | 0.14 | 0.098 | 0.127 | 0.118 | 0.111 | 0.085 | 0.001 | 0 | 0.067 | 0.14 | 0.125 | 0.066 | 0.089 | 0.106 | 0.08 | 0.072 | 0.105 | 0.111 |
| TCGA-ZJ-AAXF | low | 0 | 0.072 | 0.048 | 0 | 0.063 | 0.046 | 0.128 | 0.07 | 0.114 | 0.081 | 0.138 | 0.059 | 0 | 0.045 | 0.07 | 0.219 | 0.166 | 0.05 | 0.094 | 0.112 | 0.094 | 0.041 | 0.117 | 0.107 |
| TCGA-EA-A5ZE | low | 0 | 0.059 | 0.058 | 0 | 0.041 | 0.049 | 0.135 | 0.099 | 0.111 | 0.107 | 0.135 | 0.062 | 0 | 0.042 | 0.021 | 0.203 | 0.191 | 0.064 | 0.175 | 0.118 | 0.088 | 0.018 | 0.101 | 0.068 |
| TCGA-C5-A1BJ | low | 0 | 0.075 | 0.073 | 0 | 0.044 | 0.052 | 0.126 | 0.075 | 0.127 | 0.098 | 0.097 | 0.074 | 0 | 0.014 | 0.082 | 0.206 | 0.161 | 0.036 | 0.1 | 0.143 | 0.133 | 0.019 | 0.11 | 0.096 |
| TCGA-VS-A9V3 | low | 0 | 0.061 | 0.034 | 0 | 0.039 | 0.051 | 0.136 | 0.089 | 0.108 | 0.091 | 0.115 | 0.078 | 0 | 0.02 | 0.09 | 0.171 | 0.159 | 0.035 | 0.157 | 0.079 | 0.063 | 0.048 | 0.117 | 0.107 |
| TCGA-DG-A2KH | low | 0 | 0.071 | 0.087 | 0 | 0 | 0.056 | 0.126 | 0.087 | 0.112 | 0.133 | 0.164 | 0.073 | 0.005 | 0.035 | 0.093 | 0.087 | 0.165 | 0.08 | 0.045 | 0.084 | 0.139 | 0.038 | 0.082 | 0.053 |
| TCGA-C5-A7X8 | low | 0 | 0.08 | 0.026 | 0 | 0.041 | 0.035 | 0.112 | 0.083 | 0.106 | 0.12 | 0.076 | 0.061 | 0 | 0.065 | 0.094 | 0.173 | 0.221 | 0.054 | 0.108 | 0.136 | 0.095 | 0.016 | 0.12 | 0.097 |
| TCGA-EX-A1H6 | low | 0 | 0.03 | 0.066 | 0 | 0 | 0.047 | 0.046 | 0.097 | 0.126 | 0.118 | 0.125 | 0.076 | 0.004 | 0.064 | 0.085 | 0.106 | 0.178 | 0.07 | 0.072 | 0.169 | 0.091 | 0.066 | 0.12 | 0.075 |
| TCGA-Q1-A73P | low | 0 | 0.055 | 0.072 | 0 | 0.01 | 0.057 | 0.124 | 0.104 | 0.086 | 0.106 | 0.133 | 0.062 | 0 | 0.034 | 0.078 | 0.186 | 0.179 | 0.074 | 0.131 | 0.141 | 0.098 | 0.035 | 0.086 | 0.066 |
| TCGA-EK-A2RB | low | 0 | 0.077 | 0.034 | 0 | 0 | 0.058 | 0.146 | 0.1 | 0.123 | 0.029 | 0.102 | 0.088 | 0.001 | 0.062 | 0.007 | 0.221 | 0.115 | 0.064 | 0.145 | 0.103 | 0.108 | 0.064 | 0.041 | 0.143 |
| TCGA-EK-A2RO | low | 0 | 0.094 | 0.02 | 0 | 0 | 0.049 | 0.128 | 0.102 | 0.128 | 0.12 | 0.143 | 0.077 | 0 | 0.015 | 0.018 | 0.254 | 0.087 | 0.077 | 0.163 | 0.141 | 0.091 | 0.067 | 0.046 | 0.105 |
| TCGA-EA-A44S | low | 0 | 0.084 | 0.027 | 0 | 0 | 0.061 | 0.139 | 0.085 | 0.114 | 0.072 | 0.033 | 0.072 | 0 | 0.022 | 0.094 | 0.261 | 0.175 | 0.051 | 0.171 | 0.144 | 0.103 | 0 | 0.064 | 0.126 |
| TCGA-JW-A5VK | low | 0 | 0 | 0.07 | 0 | 0 | 0.047 | 0 | 0.118 | 0.128 | 0.112 | 0.074 | 0.063 | 0 | 0.019 | 0.101 | 0.22 | 0.207 | 0.058 | 0.143 | 0.142 | 0.133 | 0.048 | 0.092 | 0.121 |
| TCGA-JW-A852 | low | 0 | 0.078 | 0.091 | 0 | 0 | 0.051 | 0.061 | 0.126 | 0.133 | 0.107 | 0.001 | 0.071 | 0 | 0.028 | 0.111 | 0.251 | 0.05 | 0.096 | 0.168 | 0.189 | 0.073 | 0 | 0.121 | 0.085 |
| TCGA-VS-A9U5 | low | 0 | 0.05 | 0.012 | 0 | 0.068 | 0.053 | 0.125 | 0.084 | 0.113 | 0.039 | 0.137 | 0.066 | 0 | 0.021 | 0.081 | 0.217 | 0.176 | 0.075 | 0.131 | 0.14 | 0.062 | 0.052 | 0.112 | 0.108 |
| TCGA-EA-A97N | low | 0 | 0.081 | 0.009 | 0 | 0.004 | 0 | 0.118 | 0.083 | 0.113 | 0.115 | 0.121 | 0.091 | 0 | 0.076 | 0.08 | 0.215 | 0.076 | 0.055 | 0.121 | 0.128 | 0.146 | 0.051 | 0.098 | 0.046 |
| TCGA-C5-A2LX | low | 0 | 0.057 | 0.066 | 0 | 0.049 | 0.059 | 0.138 | 0.072 | 0.131 | 0.057 | 0.148 | 0.032 | 0 | 0.015 | 0.095 | 0.23 | 0.205 | 0.049 | 0.134 | 0.205 | 0.044 | 0.029 | 0.11 | 0.11 |
| TCGA-VS-A8QM | low | 0 | 0.067 | 0.077 | 0 | 0.086 | 0.053 | 0.128 | 0.057 | 0.123 | 0.07 | 0.097 | 0.051 | 0 | 0.018 | 0.083 | 0.217 | 0.236 | 0.018 | 0.087 | 0.108 | 0.096 | 0.029 | 0.121 | 0.107 |
| TCGA-ZJ-AAXB | low | 0 | 0.098 | 0.071 | 0 | 0 | 0.058 | 0.05 | 0.104 | 0.078 | 0.112 | 0.093 | 0.088 | 0 | 0.082 | 0.098 | 0.179 | 0.083 | 0.101 | 0.146 | 0.16 | 0.086 | 0.056 | 0.053 | 0.032 |
| TCGA-XS-A8TJ | low | 0 | 0.065 | 0.072 | 0 | 0.008 | 0.045 | 0.135 | 0.086 | 0.116 | 0.087 | 0.142 | 0.061 | 0 | 0.026 | 0.06 | 0.238 | 0.116 | 0.044 | 0.131 | 0.1 | 0.116 | 0.052 | 0.097 | 0.101 |
| TCGA-FU-A3WB | low | 0 | 0.099 | 0.024 | 0 | 0 | 0.056 | 0.118 | 0.108 | 0.085 | 0.124 | 0.106 | 0.087 | 0 | 0.058 | 0.076 | 0.121 | 0.046 | 0.083 | 0.105 | 0.068 | 0.088 | 0.066 | 0.094 | 0.117 |
| TCGA-VS-A8EG | low | 0 | 0.076 | 0.042 | 0 | 0.034 | 0.054 | 0.062 | 0.093 | 0.119 | 0.102 | 0.12 | 0.095 | 0 | 0.02 | 0.084 | 0.153 | 0.154 | 0.069 | 0.102 | 0.12 | 0.152 | 0.059 | 0.079 | 0.097 |
| TCGA-VS-A9UV | low | 0 | 0.068 | 0.061 | 0 | 0.025 | 0.062 | 0.13 | 0.097 | 0.123 | 0.029 | 0.12 | 0.057 | 0 | 0.04 | 0.064 | 0.18 | 0.177 | 0.077 | 0.117 | 0.086 | 0.091 | 0.058 | 0.076 | 0.101 |
| TCGA-C5-A7UE | low | 0 | 0.074 | 0.068 | 0 | 0.009 | 0.047 | 0.124 | 0.085 | 0.134 | 0.057 | 0.101 | 0.065 | 0 | 0.048 | 0.075 | 0.156 | 0.168 | 0.033 | 0.1 | 0.146 | 0.128 | 0.031 | 0.108 | 0.118 |
| TCGA-C5-A8XI | low | 0 | 0.085 | 0.046 | 0 | 0.036 | 0.051 | 0.124 | 0.085 | 0.12 | 0.09 | 0.16 | 0.071 | 0 | 0.021 | 0.075 | 0.149 | 0.174 | 0.049 | 0.08 | 0.152 | 0.119 | 0.034 | 0.117 | 0.077 |
| TCGA-JW-A5VI | low | 0 | 0.079 | 0.069 | 0 | 0.043 | 0.058 | 0.133 | 0.094 | 0.12 | 0.024 | 0.11 | 0.071 | 0 | 0.061 | 0.058 | 0.224 | 0.134 | 0.048 | 0.137 | 0.144 | 0.106 | 0.034 | 0.081 | 0.115 |
| TCGA-UC-A7PF | low | 0 | 0.06 | 0.063 | 0 | 0.05 | 0.05 | 0.132 | 0.079 | 0.115 | 0.06 | 0.121 | 0.072 | 0 | 0.026 | 0.071 | 0.244 | 0.166 | 0.065 | 0.151 | 0.14 | 0.106 | 0.043 | 0.093 | 0.077 |
| TCGA-DG-A2KM | low | 0 | 0.056 | 0.064 | 0 | 0.098 | 0.059 | 0.143 | 0.056 | 0.121 | 0.043 | 0.094 | 0.035 | 0 | 0 | 0.065 | 0.249 | 0.23 | 0.019 | 0.177 | 0.154 | 0.066 | 0.05 | 0.113 | 0.102 |
| TCGA-VS-A954 | low | 0 | 0.071 | 0.03 | 0 | 0.01 | 0.058 | 0.135 | 0.085 | 0.116 | 0.097 | 0.12 | 0.077 | 0 | 0.056 | 0.064 | 0.184 | 0.173 | 0.067 | 0.157 | 0.104 | 0.089 | 0.037 | 0.064 | 0.101 |
| TCGA-EA-A5ZD | low | 0 | 0.065 | 0.009 | 0 | 0.036 | 0.054 | 0.134 | 0.102 | 0.114 | 0.089 | 0.119 | 0.051 | 0 | 0.028 | 0.076 | 0.255 | 0.112 | 0.083 | 0.18 | 0.137 | 0.113 | 0.018 | 0.102 | 0.091 |
| TCGA-EA-A1QT | low | 0 | 0.077 | 0.067 | 0 | 0 | 0.054 | 0.137 | 0.105 | 0.117 | 0.094 | 0.102 | 0.067 | 0 | 0.033 | 0.062 | 0.242 | 0.058 | 0.021 | 0.153 | 0.13 | 0.11 | 0.043 | 0.081 | 0.111 |
| TCGA-EK-A2PG | low | 0 | 0.081 | 0.058 | 0 | 0 | 0.049 | 0.108 | 0.096 | 0.123 | 0.103 | 0.123 | 0.064 | 0 | 0.012 | 0.077 | 0.227 | 0.123 | 0.051 | 0.134 | 0.092 | 0.089 | 0.061 | 0.056 | 0.11 |
| TCGA-EA-A3HS | low | 0 | 0.045 | 0.05 | 0 | 0.004 | 0.06 | 0.139 | 0.083 | 0.121 | 0.117 | 0.115 | 0.085 | 0 | 0.02 | 0.078 | 0.174 | 0.11 | 0.082 | 0.15 | 0.208 | 0.082 | 0.042 | 0.083 | 0.094 |
| TCGA-JX-A5QV | low | 0 | 0.082 | 0.083 | 0 | 0 | 0.06 | 0.136 | 0.116 | 0.113 | 0.096 | 0.114 | 0.061 | 0 | 0.02 | 0.004 | 0.288 | 0.16 | 0.078 | 0.158 | 0.12 | 0.091 | 0.041 | 0.053 | 0.116 |
| TCGA-Q1-A73S | low | 0 | 0.125 | 0.072 | 0 | 0 | 0.054 | 0.011 | 0.135 | 0 | 0.098 | 0.137 | 0.04 | 0 | 0.002 | 0.075 | 0.25 | 0.057 | 0.044 | 0.198 | 0.068 | 0.098 | 0.076 | 0.047 | 0.102 |
| TCGA-LP-A7HU | low | 0 | 0.065 | 0.045 | 0 | 0.017 | 0.054 | 0.116 | 0.097 | 0.07 | 0.07 | 0.106 | 0.062 | 0 | 0.064 | 0.089 | 0.167 | 0.225 | 0.049 | 0.162 | 0.108 | 0.072 | 0.043 | 0.072 | 0.113 |
| TCGA-MY-A5BF | low | 0 | 0.08 | 0.016 | 0.001 | 0.033 | 0.067 | 0.139 | 0.123 | 0.119 | 0.141 | 0 | 0.09 | 0 | 0.032 | 0.088 | 0.277 | 0.006 | 0.084 | 0.169 | 0.112 | 0.076 | 0.011 | 0.041 | 0.146 |
| TCGA-VS-A9U7 | low | 0 | 0.056 | 0.076 | 0 | 0.005 | 0.056 | 0.13 | 0.104 | 0.116 | 0.072 | 0.099 | 0.055 | 0 | 0.028 | 0.096 | 0.26 | 0.147 | 0.016 | 0.156 | 0.145 | 0.108 | 0.035 | 0.079 | 0.099 |
| TCGA-FU-A23L | low | 0 | 0.062 | 0.058 | 0 | 0.038 | 0.056 | 0.131 | 0.09 | 0.111 | 0.089 | 0.11 | 0.063 | 0 | 0.019 | 0.062 | 0.161 | 0.198 | 0.037 | 0.119 | 0.133 | 0.115 | 0.036 | 0.106 | 0.106 |
| TCGA-DS-A3LQ | low | 0 | 0.074 | 0.057 | 0 | 0.023 | 0.064 | 0.123 | 0.107 | 0.125 | 0.041 | 0.083 | 0.084 | 0 | 0.01 | 0.074 | 0.257 | 0.055 | 0.055 | 0.206 | 0.174 | 0.047 | 0.027 | 0.102 | 0.096 |
| TCGA-VS-A9UU | low | 0 | 0.084 | 0.044 | 0 | 0.045 | 0.04 | 0.131 | 0.099 | 0.128 | 0.105 | 0.125 | 0.081 | 0 | 0.044 | 0.051 | 0.129 | 0.202 | 0.044 | 0.039 | 0.166 | 0.143 | 0.034 | 0.068 | 0.096 |
| TCGA-EK-A2RA | low | 0 | 0.065 | 0.021 | 0 | 0 | 0.055 | 0.118 | 0.091 | 0.108 | 0.096 | 0.136 | 0.048 | 0 | 0.04 | 0.082 | 0.228 | 0.191 | 0.069 | 0.14 | 0.111 | 0.082 | 0.047 | 0.104 | 0.113 |
| TCGA-FU-A3TQ | low | 0 | 0.068 | 0.08 | 0 | 0.036 | 0.044 | 0.134 | 0.075 | 0.109 | 0.09 | 0.141 | 0.072 | 0 | 0.021 | 0.086 | 0.204 | 0.18 | 0.034 | 0.108 | 0.119 | 0.14 | 0.011 | 0.081 | 0.095 |
| TCGA-C5-A8ZZ | low | 0 | 0.092 | 0.042 | 0 | 0.021 | 0.048 | 0.129 | 0.1 | 0.124 | 0.069 | 0.125 | 0.084 | 0 | 0.088 | 0 | 0.173 | 0.029 | 0.068 | 0.153 | 0.132 | 0.073 | 0.036 | 0.102 | 0.113 |
| TCGA-Q1-A73R | low | 0 | 0.09 | 0.055 | 0 | 0 | 0.057 | 0.136 | 0.099 | 0.097 | 0.061 | 0.098 | 0.072 | 0 | 0.067 | 0.077 | 0.1 | 0.09 | 0.037 | 0.105 | 0.167 | 0.129 | 0.067 | 0.086 | 0.086 |
| TCGA-C5-A1BE | low | 0 | 0.077 | 0.027 | 0 | 0.011 | 0.056 | 0.127 | 0.112 | 0.12 | 0.076 | 0.163 | 0.08 | 0 | 0.063 | 0.008 | 0.245 | 0.087 | 0.039 | 0.148 | 0.097 | 0.09 | 0.031 | 0.117 | 0.111 |
| TCGA-VS-A8QF | low | 0 | 0.053 | 0.082 | 0 | 0.036 | 0.045 | 0.134 | 0.093 | 0.113 | 0.114 | 0.082 | 0.061 | 0 | 0 | 0.076 | 0.238 | 0.191 | 0.037 | 0.186 | 0.139 | 0.109 | 0.032 | 0.054 | 0.107 |
| TCGA-VS-A8EK | low | 0 | 0.058 | 0.091 | 0 | 0.019 | 0.057 | 0.127 | 0.075 | 0.107 | 0.104 | 0.138 | 0.056 | 0 | 0.037 | 0.048 | 0.165 | 0.164 | 0.078 | 0.124 | 0.144 | 0.144 | 0.036 | 0.068 | 0.098 |
| TCGA-EK-A2RK | low | 0 | 0.059 | 0.047 | 0 | 0 | 0.052 | 0.131 | 0.114 | 0.126 | 0.027 | 0.123 | 0.062 | 0 | 0.033 | 0.082 | 0.208 | 0.115 | 0.036 | 0.129 | 0.168 | 0.106 | 0.052 | 0.098 | 0.133 |
| TCGA-JW-AAVH | low | 0 | 0.084 | 0.063 | 0 | 0 | 0.052 | 0.133 | 0.101 | 0.126 | 0.124 | 0.141 | 0.088 | 0 | 0.017 | 0.005 | 0.178 | 0.104 | 0.045 | 0.058 | 0.138 | 0.054 | 0.072 | 0.115 | 0.124 |
| TCGA-MY-A5BD | low | 0 | 0.067 | 0.017 | 0 | 0 | 0.066 | 0.132 | 0.094 | 0.117 | 0.103 | 0.125 | 0.066 | 0 | 0.084 | 0.102 | 0.172 | 0.085 | 0.072 | 0.066 | 0.123 | 0.055 | 0.052 | 0.125 | 0.119 |
| TCGA-Q1-A5R1 | low | 0 | 0.076 | 0.033 | 0 | 0.011 | 0 | 0.128 | 0.087 | 0.119 | 0.082 | 0.177 | 0.069 | 0 | 0.059 | 0.092 | 0.152 | 0.175 | 0.055 | 0.113 | 0.159 | 0.062 | 0.05 | 0.106 | 0.001 |
| TCGA-C5-A3HL | low | 0 | 0.065 | 0.021 | 0 | 0.011 | 0.051 | 0.124 | 0.082 | 0.122 | 0.112 | 0.161 | 0.081 | 0 | 0.042 | 0.069 | 0.144 | 0.126 | 0.075 | 0.103 | 0.152 | 0.079 | 0.053 | 0.091 | 0.118 |
| TCGA-WL-A834 | low | 0 | 0.086 | 0.029 | 0 | 0 | 0.059 | 0.109 | 0.084 | 0.117 | 0.12 | 0.113 | 0.079 | 0 | 0.071 | 0.08 | 0.256 | 0.064 | 0.043 | 0.117 | 0.127 | 0.063 | 0.048 | 0.086 | 0.095 |
| TCGA-VS-A9V5 | low | 0 | 0.068 | 0.064 | 0 | 0 | 0 | 0.133 | 0.066 | 0.107 | 0.13 | 0.13 | 0.072 | 0 | 0.068 | 0.091 | 0.095 | 0.195 | 0.069 | 0.07 | 0.105 | 0.145 | 0.057 | 0.104 | 0.012 |
| TCGA-EK-A2RC | low | 0 | 0.072 | 0.059 | 0 | 0.015 | 0.051 | 0.122 | 0.081 | 0.117 | 0.073 | 0.105 | 0.076 | 0 | 0.031 | 0.067 | 0.202 | 0.158 | 0.049 | 0.153 | 0.161 | 0.088 | 0.028 | 0.101 | 0.112 |
| TCGA-EX-A1H5 | low | 0 | 0.068 | 0.074 | 0 | 0.011 | 0.05 | 0.125 | 0.103 | 0.121 | 0.09 | 0.08 | 0.087 | 0 | 0.065 | 0.078 | 0.129 | 0.183 | 0.039 | 0.066 | 0.113 | 0.077 | 0 | 0.104 | 0.137 |
| TCGA-VS-A8Q9 | low | 0 | 0.072 | 0.031 | 0 | 0.001 | 0.061 | 0.134 | 0.102 | 0.107 | 0.079 | 0.124 | 0.061 | 0 | 0.053 | 0.086 | 0.116 | 0.094 | 0.083 | 0.125 | 0.156 | 0.104 | 0.07 | 0.11 | 0.095 |
| TCGA-C5-A8YT | low | 0 | 0.084 | 0.076 | 0 | 0 | 0.038 | 0.131 | 0.072 | 0.115 | 0.093 | 0.116 | 0.059 | 0 | 0.012 | 0.082 | 0.099 | 0.16 | 0.033 | 0.075 | 0.181 | 0.138 | 0.062 | 0.115 | 0.098 |
| TCGA-MU-A5YI | low | 0 | 0.072 | 0.045 | 0 | 0.018 | 0.052 | 0.118 | 0.086 | 0.112 | 0.107 | 0.143 | 0.072 | 0 | 0.038 | 0.086 | 0.218 | 0.078 | 0.042 | 0.123 | 0.15 | 0.035 | 0.056 | 0.106 | 0.115 |
| TCGA-VS-A9UZ | low | 0 | 0.064 | 0.074 | 0 | 0.035 | 0.048 | 0.106 | 0.097 | 0.112 | 0.078 | 0.143 | 0.054 | 0 | 0.055 | 0.064 | 0.15 | 0.234 | 0.051 | 0.092 | 0.164 | 0.071 | 0.047 | 0.117 | 0.053 |
| TCGA-DS-A0VL | low | 0 | 0.067 | 0.052 | 0 | 0 | 0.055 | 0.14 | 0.088 | 0.121 | 0.106 | 0.151 | 0.079 | 0 | 0.023 | 0.053 | 0.192 | 0.078 | 0.047 | 0.136 | 0.136 | 0.056 | 0.062 | 0.093 | 0.113 |
| TCGA-VS-A8EI | low | 0 | 0.059 | 0.101 | 0 | 0.052 | 0.061 | 0.128 | 0.108 | 0.121 | 0.045 | 0.083 | 0.048 | 0 | 0.062 | 0.009 | 0.177 | 0.178 | 0.062 | 0.125 | 0.149 | 0.117 | 0.03 | 0.109 | 0.105 |
| TCGA-Q1-A6DW | low | 0 | 0.053 | 0.048 | 0 | 0.012 | 0.061 | 0.116 | 0.078 | 0.108 | 0.145 | 0.113 | 0.087 | 0 | 0 | 0.091 | 0.213 | 0.104 | 0.082 | 0.122 | 0.161 | 0.099 | 0.068 | 0.073 | 0.107 |
| TCGA-FU-A3EO | low | 0 | 0 | 0.063 | 0 | 0 | 0.046 | 0.02 | 0.109 | 0.147 | 0.128 | 0.172 | 0.071 | 0.003 | 0 | 0.06 | 0.289 | 0.105 | 0.096 | 0.195 | 0.167 | 0.045 | 0.063 | 0.063 | 0.079 |
| TCGA-FU-A3TX | low | 0 | 0.096 | 0.011 | 0 | 0.038 | 0.081 | 0.108 | 0.099 | 0.126 | 0.073 | 0.084 | 0.054 | 0 | 0 | 0.113 | 0.215 | 0.141 | 0.089 | 0.139 | 0.122 | 0.118 | 0.038 | 0.103 | 0.091 |
| TCGA-C5-A7CG | low | 0 | 0.062 | 0.062 | 0 | 0.054 | 0.057 | 0.136 | 0.069 | 0.107 | 0.087 | 0.085 | 0.046 | 0 | 0.011 | 0.069 | 0.199 | 0.218 | 0 | 0.137 | 0.151 | 0.106 | 0.035 | 0.109 | 0.106 |
| TCGA-C5-A905 | low | 0 | 0.056 | 0.098 | 0 | 0 | 0.037 | 0.137 | 0.086 | 0.123 | 0.11 | 0.159 | 0.076 | 0 | 0.06 | 0 | 0.18 | 0.09 | 0.011 | 0.126 | 0.167 | 0.1 | 0.039 | 0.097 | 0.117 |
| TCGA-RA-A741 | low | 0 | 0.055 | 0.088 | 0 | 0.037 | 0.04 | 0.133 | 0.087 | 0.126 | 0.092 | 0.103 | 0.038 | 0 | 0.034 | 0.073 | 0.224 | 0.186 | 0.073 | 0.149 | 0.142 | 0.114 | 0.023 | 0.098 | 0.058 |
| TCGA-FU-A3NI | low | 0 | 0.057 | 0.043 | 0 | 0.025 | 0.06 | 0.123 | 0.097 | 0.112 | 0.119 | 0.129 | 0.066 | 0 | 0.018 | 0.064 | 0.163 | 0.166 | 0.033 | 0.139 | 0.113 | 0.097 | 0.061 | 0.085 | 0.097 |
| TCGA-EK-A3GJ | low | 0 | 0.062 | 0.057 | 0 | 0 | 0.062 | 0.131 | 0.128 | 0.116 | 0.121 | 0.135 | 0.069 | 0 | 0.015 | 0.064 | 0.237 | 0.097 | 0.052 | 0.2 | 0.107 | 0.05 | 0.035 | 0.06 | 0.129 |
| TCGA-EK-A2R9 | low | 0 | 0.09 | 0.069 | 0 | 0 | 0.063 | 0.14 | 0.093 | 0.125 | 0.114 | 0.071 | 0.047 | 0 | 0.025 | 0.033 | 0.208 | 0.167 | 0.073 | 0.125 | 0.112 | 0.093 | 0.044 | 0.104 | 0.111 |
| TCGA-ZJ-AAXD | low | 0 | 0.089 | 0.007 | 0 | 0.019 | 0.042 | 0.121 | 0.096 | 0.127 | 0.082 | 0.15 | 0.093 | 0 | 0.018 | 0.076 | 0.136 | 0.132 | 0.059 | 0.097 | 0.112 | 0.128 | 0.058 | 0.105 | 0.12 |
| TCGA-DS-A1OD | low | 0 | 0.073 | 0.062 | 0 | 0.047 | 0.062 | 0.134 | 0.069 | 0.113 | 0.103 | 0.095 | 0.036 | 0 | 0.019 | 0.103 | 0.185 | 0.194 | 0.051 | 0.141 | 0.145 | 0.054 | 0.034 | 0.118 | 0.111 |
| TCGA-C5-A1MI | low | 0 | 0.086 | 0.046 | 0 | 0.052 | 0.049 | 0.13 | 0.097 | 0.109 | 0.051 | 0.147 | 0.069 | 0 | 0.03 | 0.09 | 0.181 | 0.107 | 0.008 | 0.126 | 0.124 | 0.069 | 0.05 | 0.104 | 0.113 |
| TCGA-VS-A8EL | low | 0 | 0.059 | 0.064 | 0 | 0.03 | 0.053 | 0.14 | 0.075 | 0.12 | 0.103 | 0.093 | 0.046 | 0 | 0.056 | 0.02 | 0.257 | 0.129 | 0.053 | 0.188 | 0.165 | 0.125 | 0.03 | 0.064 | 0.118 |
| TCGA-MY-A5BE | low | 0 | 0.048 | 0.07 | 0 | 0.071 | 0.063 | 0.138 | 0.078 | 0.111 | 0.038 | 0.107 | 0.036 | 0 | 0 | 0.07 | 0.206 | 0.265 | 0.021 | 0.157 | 0.154 | 0.063 | 0.047 | 0.129 | 0.079 |
| TCGA-Q1-A5R2 | low | 0 | 0.056 | 0.073 | 0 | 0.007 | 0.049 | 0.132 | 0.111 | 0.112 | 0.089 | 0.077 | 0.066 | 0 | 0.062 | 0.009 | 0.211 | 0.165 | 0.058 | 0.215 | 0.138 | 0.089 | 0.043 | 0.05 | 0.123 |
| TCGA-DS-A1OA | low | 0 | 0.06 | 0.037 | 0 | 0.036 | 0.058 | 0.137 | 0.088 | 0.108 | 0.094 | 0.085 | 0.073 | 0 | 0.053 | 0.085 | 0.266 | 0.167 | 0.043 | 0.153 | 0.091 | 0.046 | 0.033 | 0.104 | 0.102 |
| TCGA-C5-A8XJ | low | 0 | 0.088 | 0.066 | 0 | 0.024 | 0.056 | 0.129 | 0.089 | 0.106 | 0.062 | 0.093 | 0.068 | 0 | 0.041 | 0.095 | 0.183 | 0.145 | 0.072 | 0.126 | 0.098 | 0.082 | 0.052 | 0.102 | 0.103 |
| TCGA-LP-A5U2 | low | 0 | 0.084 | 0.033 | 0 | 0.002 | 0.089 | 0.107 | 0.13 | 0.138 | 0.031 | 0.12 | 0.095 | 0 | 0.014 | 0.002 | 0.196 | 0.149 | 0.08 | 0.153 | 0.068 | 0.113 | 0.058 | 0.084 | 0.128 |
| TCGA-VS-A9V2 | low | 0 | 0.061 | 0.036 | 0 | 0.046 | 0.048 | 0.125 | 0.084 | 0.104 | 0.088 | 0.108 | 0.059 | 0 | 0.046 | 0.08 | 0.257 | 0.122 | 0.055 | 0.126 | 0.138 | 0.119 | 0.04 | 0.089 | 0.112 |
| TCGA-C5-A3HF | low | 0 | 0.072 | 0.028 | 0 | 0.043 | 0.053 | 0.133 | 0.084 | 0.115 | 0.121 | 0.095 | 0.07 | 0 | 0.054 | 0.078 | 0.207 | 0.176 | 0.042 | 0.068 | 0.123 | 0.092 | 0.033 | 0.108 | 0.107 |
| TCGA-MA-AA3Z | low | 0 | 0.075 | 0.036 | 0 | 0.028 | 0.053 | 0.13 | 0.127 | 0.13 | 0.041 | 0.106 | 0.081 | 0 | 0 | 0.083 | 0.259 | 0.044 | 0.074 | 0.14 | 0.107 | 0.036 | 0.065 | 0.127 | 0.135 |
| TCGA-EK-A2PM | low | 0 | 0.097 | 0.038 | 0 | 0 | 0 | 0.044 | 0.117 | 0.143 | 0.098 | 0 | 0.047 | 0 | 0.086 | 0.09 | 0.167 | 0.05 | 0.111 | 0.14 | 0.011 | 0.131 | 0.02 | 0.042 | 0.104 |
| TCGA-VS-A9UD | low | 0 | 0.055 | 0.028 | 0.005 | 0.045 | 0.059 | 0.137 | 0.121 | 0.082 | 0.068 | 0.073 | 0.056 | 0 | 0.006 | 0.091 | 0.25 | 0.191 | 0.061 | 0.214 | 0.141 | 0 | 0.059 | 0.078 | 0.097 |
| TCGA-C5-A2M2 | low | 0 | 0.093 | 0.01 | 0 | 0 | 0.071 | 0.115 | 0.106 | 0.045 | 0.153 | 0.142 | 0.09 | 0.009 | 0.062 | 0.067 | 0.094 | 0.061 | 0.022 | 0.057 | 0.134 | 0.081 | 0.058 | 0.096 | 0.12 |
